# Solvent Precipitation SP3 (SP4) Enhances Recovery for Proteomics Sample Preparation without Magnetic Beads

**DOI:** 10.1021/acs.analchem.1c04200

**Published:** 2022-07-18

**Authors:** Harvey E. Johnston, Kranthikumar Yadav, Joanna M. Kirkpatrick, George S. Biggs, David Oxley, Holger B. Kramer, Rahul S. Samant

**Affiliations:** † Signalling Programme, The Babraham Institute, Cambridge CB22 3AT, United Kingdom; ‡ Mass Spectrometry Facility, The Babraham Institute, Cambridge CB22 3AT, United Kingdom; § Proteomics STP, The Francis Crick Institute, London NW1 1AT, United Kingdom; ∥ GlaxoSmithKline, Gunnels Wood Road, Stevenage SG1 2NY, Hertfordshire, United Kingdom; ⊥ Medical Research Council London Institute of Medical Sciences, Imperial College London, Hammersmith Hospital, London W12 0NN, United Kingdom

## Abstract

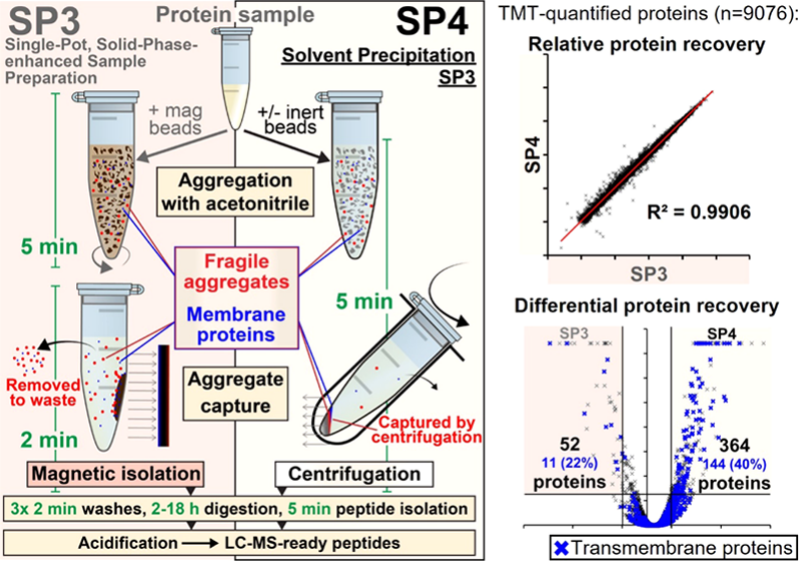

Complete, reproducible extraction of protein material is essential for comprehensive and unbiased proteome analyses. A current gold standard is single-pot, solid-phase-enhanced sample preparation (SP3), in which organic solvent and magnetic beads are used to denature and capture protein aggregates, with subsequent washes removing contaminants. However, SP3 is dependent on effective protein immobilization onto beads, risks losses during wash steps, and exhibits losses and greater costs at higher protein inputs. Here, we propose solvent precipitation SP3 (SP4) as an alternative to SP3 protein cleanup, capturing acetonitrile-induced protein aggregates by brief centrifugation rather than magnetism—with optional low-cost inert glass beads to simplify handling. SP4 recovered equivalent or greater protein yields for 1–5000 μg preparations and improved reproducibility (median protein *R*^2^ 0.99 (SP4) *vs* 0.97 (SP3)). Deep proteome profiling revealed that SP4 yielded a greater recovery of low-solubility and transmembrane proteins than SP3, benefits to aggregating protein using 80 *vs* 50% organic solvent, and equivalent recovery by SP4 and S-Trap. SP4 was verified in three other labs across eight sample types and five lysis buffers—all confirming equivalent or improved proteome characterization *vs* SP3. With near-identical recovery, this work further illustrates protein precipitation as the primary mechanism of SP3 protein cleanup and identifies that magnetic capture risks losses, especially at higher protein concentrations and among more hydrophobic proteins. SP4 offers a minimalistic approach to protein cleanup that provides cost-effective input scalability, the option to omit beads entirely, and suggests important considerations for SP3 applications—all while retaining the speed and compatibility of SP3.

## Introduction

Proteomics experiments typically aim to characterize comprehensively all proteins present in a given sample.^1^ Extraction of protein material from complex biological mixtures generally requires use of buffers containing components incompatible with several stages of proteomics analysis (*e.g*., detergents, salts).^2^ Although several cleanup methods exist,^3−6^ contaminant removal represents a major source of sample losses and experimental variability.^7^

An increasingly popular sample preparation method is SP3 (single-pot, solid-phase-enhanced sample preparation), employing a single reaction vessel, carboxylate-modified magnetic beads (CMMBs), and organic solvent-induced protein aggregation to wash away contaminants.^5,8−10^ SP3 is a fast, effective, high-throughput, and relatively streamlined protocol—compatible with automation and a range of protein inputs, with diverse proteomics applications.^5,11−16^ Improvements on the initially proposed protein cleanup method include neutral pH, solvent adjustments, and a more rapid workflow taking around 90 min from cells to peptides.^5,10,11^

However, SP3 has the potential for losses and variability, *e.g*., if protein aggregates do not completely adhere to magnetic beads, if aggregates are disrupted during wash steps, or if technical steps are not followed carefully.^9^ Larger protein inputs (*e.g*., for enrichment of post-translational modifications (PTMs)) are also disadvantaged by counter-intuitive losses and bead costs.^5,11^ Furthermore, CMMBs present a physical contamination risk and the potential to bind protease inhibitors.^14^

Although the mechanism of SP3 was originally proposed to involve hydrophilic interaction chromatography (HILIC)-like solid-phase interaction between CMMBs and proteins, Batth et al. recently demonstrated that protein recovery for SP3 is not dependent on bead surface chemistry.^13^ Their work suggests that HILIC-like interactions are not the primary form of solid-phase bead–protein interactions. Instead, the authors described the SP3 mechanism as protein aggregation capture (PAC), driven by organic solvent-induced denaturation. PAC, and therefore SP3, bear a striking mechanistic similarity to protein precipitation—a well-established purification approach that typically employs organic solvents to induce protein denaturation and precipitation into insoluble aggregates. However, protein precipitation has historically been associated with extended incubation steps, incomplete protein capture, and chemical modification of proteins and/or peptides.^17−21^ Nevertheless, several recent methods have demonstrated that combining protein precipitation with filter-based trapping provides a rapid means of protein capture and cleanup.^22−24^ The importance of ionic strength (>10 mM NaCl) was demonstrated to be essential for protein precipitation, allowing the reaction to complete in as little as 2 min.^25^

Building upon the SP3 developments of Batth et al.,^13^ here we omit magnetic beads entirely and instead employ acetonitrile (ACN)-induced protein precipitation and centrifugation for protein capture and isolation—either bead-free (BF), or with low-cost, inert glass beads (GB). We name this method SP4 or *S*olvent *P*recipitation SP3. Both SP4 variants matched or outperformed SP3 across a variety of applications and settings, with SP4-GB offering technical advantages and some higher recovery than SP4-BF. SP4 also yielded equivalent results to S-Trap. We provide further evidence that protein precipitation is the primary mechanism of SP3 protein enrichment. We therefore propose that CMMBs, while advantageous in specific settings (*e.g*., peptide fractionation and automation^14−16^), can be replaced with inert glass beads—or omitted altogether—without adversely affecting proteome recovery, provided protein input and concentration are sufficiently high (>1 μg and >0.25 μg/μL, respectively). Furthermore, magnetic capture in SP3 increased the risk of protein aggregate losses—especially of low-solubility (*e.g*., membrane) proteins and at higher protein concentrations. SP4 offers a minimalistic, low-cost protein cleanup approach (especially for high-input preparations, *e.g*., prior to PTM analyses), is easy to use for non-proteomics scientists, requires no specialized equipment or reagents, offers the option to omit beads entirely, and improves recovery of hydrophobic proteins—while retaining the speed and broad compatibility of SP3.

## Methods

### SP3/SP4 Preparations

Full methods and materials are provided in the Supporting Information, alongside a detailed step-by-step protocol. HEK293 cells were lysed using trituration in “SP3 lysis buffer” (50 mM HEPES pH 8.0, 1% SDS, 1% Triton X-100, 1% NP-40, 1% Tween 20, 1% sodium deoxycholate, 50 mM NaCl, 5 mM EDTA, 1% (v/v) glycerol) supplemented with 10 mM DTT, 1× cOmplete protease inhibitor, and 40 mM 2-chloroacetamide, followed by heating at 95 °C for 5 min and sonication on ice for 12 × 5 s bursts. Lysates were adjusted to 5 μg/μL. Silica beads/glass spheres (9–13 μm mean particle diameter; Sigma catalogue no. 440345) were suspended at an initial concentration of 100 mg/mL in Milli-Q water, washed sequentially with ACN, 100 mM ammonium bicarbonate (ABC), and 2× with water, pelleted at 16,000*g* for 1 min, and the supernatant was discarded (also removing any unpelleted beads). Glass beads were adjusted to a final concentration of 50 mg/mL in water or 12.5 mg/mL in ACN. A 10:1 bead/protein ratio for SP3 and SP4-GB, or an equivalent volume of water for SP4-BF experiments, was added to lysates and gently mixed at 400 rpm. Then, 4 volumes of 100% ACN was added, and tubes were mixed for 5 s at 400 rpm. Alternatively, glass beads were added to lysate presuspended in ACN. SP3 samples were incubated at 25 °C for 5 min at 800 rpm on a Thermomixer Comfort and placed on a magnetic rack for 2 min. SP4 samples were centrifuged for 5 min at 16,000*g*. Supernatants were aspirated and carefully washed 3× with 80% ethanol. Each wash used either a 2-min magnetic separation (SP3) or 2-min centrifugation at 16,000*g* (SP4, cSP3). Protein aggregates were digested with 1:100 trypsin:protein ratio in 100 mM ABC for 18 h at 37 °C at 1000 rpm on a Thermomixer Comfort. For TMT labeling, 100 μg of protein was processed, and 100 mM triethylammonium bicarbonate (TEAB) with 1:100 trypsin and Lys-C were added. Peptide solutions were isolated by removal of magnetic beads (MagRack and 16,000*g*, SP3) or beads and insoluble debris (16,000*g*, SP4) for 2 min. Peptide yields for optimization were assessed using the Pierce Quantitative Fluorometric Peptide Assay (Thermo Scientific) according to the manufacturer’s instructions. After digestion, peptides were acidified with 2% ACN and 0.1% trifluoroacetic acid and were sufficiently clean for LC-MS injection.

### S-Trap, Spin Filter, and SP4 Protein Cleanup

HEK293 lysate was prepared with 5% SDS and 50 mM TEAB as recommended by the S-Trap mini protocol. Briefly, 100 μg of the same lysate was processed for all samples (*n* = 4, label-free; *n* = 2, TMT). For S-Trap, the manufacturer’s recommended protocol was followed for mini columns. For spin filtration, a nylon 0.22 μm spin filter was used to capture the precipitate. For SP4-GB, the protein was precipitated with an ACN–bead suspension, and the described SP4 protocol was followed. Digests were performed with 5 μg of trypsin and 2 μg of Lys-C in 125 μL of 50 mM TEAB for 2 h. Peptide solutions were lyophilized and reconstituted in 100 μL of 100 mM TEAB.

### TMT Labeling and Peptide Fractionation

Briefly, 100 μg of peptides were labeled with 0.2 mg of TMT labeling reagent according to the manufacturer’s instructions. Labeled peptides were vacuum-concentrated, then reconstituted, pooled, and resolved using high-pH RP C18 chromatography over a 105-min gradient.

### LC-MS Acquisition and Analysis

Label-free analyses of peptides were acquired over 120 min by a Q-Exactive Plus Orbitrap MS (Thermo Scientific) from 100 ng of peptides (as a proportion of protein input). TMT-labeled peptide fractions were analyzed over 60 or 120 min by an Orbitrap Eclipse MS (Thermo Scientific) using SPS MS^3^ mode. Raw files were processed and analyzed with Proteome Discoverer 2.5, searching against UniProt Swiss-Prot (version 2021_01, canonical). Additional analysis was performed in Microsoft Excel. The MS proteomics data have been deposited to the ProteomeXchange Consortium (http://proteomecentral.proteomexchange.org) via the PRIDE partner repository^26^ with the data set identifier PXD032095 and, for validation work, PXD028736 and PXD028768. Proteomics data are detailed in Tables S1–S20. Annotation enrichment was performed with DAVID and PANTHER. Additional analyses were performed with CamSol,^27^ the PROMPT tool,^28^ and Proteome-pI.^29^

## Results

### Single-Pot Solvent Precipitation with Acetonitrile Provides Effective Protein Capture and Cleanup

**Figure 1:**
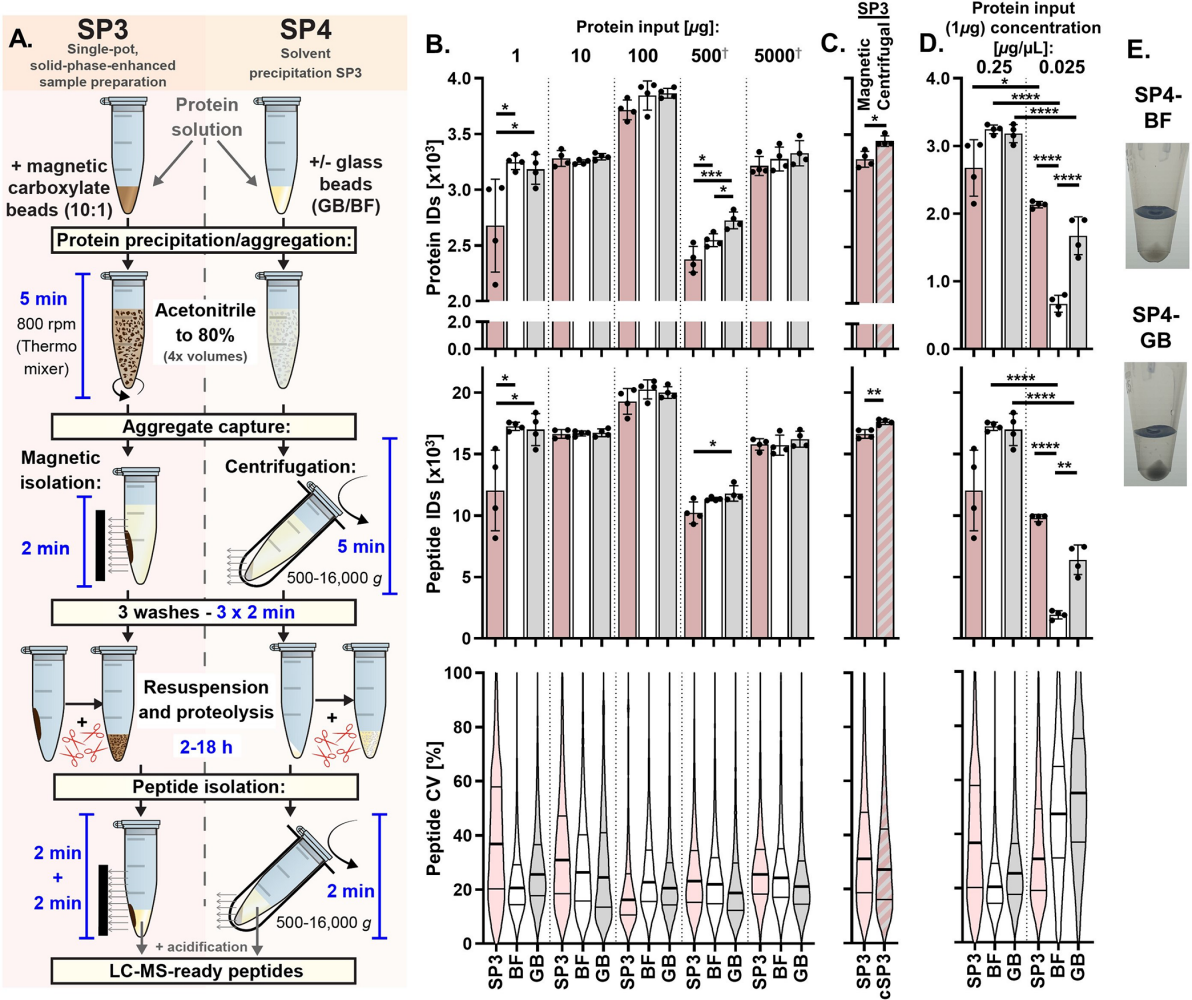
Comparison of SP3 with SP4. (A) Summary of the SP3 and SP4 workflows. For both approaches, protein in solution is aggregated with acetonitrile in the presence of carboxylate-modified magnetic beads (SP3), glass beads (SP4-GB), or bead-free (SP4-BF), captured by magnetism (SP3) or centrifugation (SP4), and contaminants removed with 3 washes prior to protein digestion—yielding peptides sufficiently clean for LC-MS injection. (B) Protein and peptide identifications and peptide coefficient of variance (CV) (as violin plots; thick line–median, thin lines–quartiles) for 1–5000 μg preparations of HEK293 cell lysate by SP3, SP4-BF, and SP4-GB (*n* = 4). Protein concentrations were 0.25 μg/μL for 1 and 10 μg (in 4 and 40 μL) and 2.5 μg/μL for 100, 500, and 5000 μg (in 40, 200 and 2000 μL). A 10:1 bead:protein ratio was used in all SP3 and SP4-GB experiments. ^**†**^500 and 5000 μg preparations were digested with TrypZean instead of MS-grade trypsin. (C) Aliquots of 10 μg of protein processed with carboxylate-modified magnetic beads and captured by either standard magnetic capture (SP3) or centrifugal (16,000*g*) SP3 (cSP3) (D) Aliquots of 1 μg of protein preparations processed at 0.025 and 0.25 μg/μL. (E) Aliquots of 50 μg of protein precipitated in the presence of 500 μg (10:1) of glass beads, offering increased pellet visibility and definition *vs* bead-free precipitation. Bar charts present median and standard deviation, with significance assessed by ANOVA (B, D) and *t*-test (C). Protein coefficients of variance distributions represented by violin plots (thick line–median, thin lines–quartiles). *p < 0.05, **p < 0.01, ***p < 0.001, ****p < 0.0001, and ns–not significant.

Building on previous mechanistic observations of SP3, we wanted to explore further the hypothesis that protein capture observed in SP3 is primarily a product of solvent-induced denaturation, aggregation, and subsequent precipitation, rather than being dependent on bead surface chemistry.^13^ We noticed that 80% ACN, similar to the conditions used to aggregate proteins during SP3, is also employed in the effective exclusion of proteins from peptidomics and metabolomics analyses through precipitation—termed a protein ‘crash.’^30−33^ As magnetic capture risks losses from incomplete, fragile, or disrupted aggregate adhesion, and 80% ACN effectively precipitates proteins, we hypothesized that centrifugation-based capture could be combined with aspects of the SP3 protocol to provide a more effective means of sample cleanup for proteomics (Figures 1A and S1). The protocol was also adapted to incorporate many of the recent optimizations to SP3, including neutral pH, higher ACN concentration for aggregation, and no reconstitution of the protein–bead aggregates.^5,10,11^

We named our optimized protocol SP4, or *S*olvent *P*recipitation *SP3*. Two variants were devised: one without any beads (bead-free, SP4-BF), thus relying on precipitation alone, and a second with inert, low-cost silica particles (hereafter termed glass beads, SP4-GB), allowing us to explore the role of surface area independently of bead chemistry. Initially, a broad range of SP4 parameters were evaluated by peptide yield, including 40–95% ACN, 0:1–160:1 glass bead:protein ratios, and 0.5–20 min centrifugation times (Figure S1). These experiments demonstrated that parameters equivalent to SP3, i.e., 80% ACN, a 10:1 glass bead:protein ratio, and 5- and 2-min protein capture steps were also the most effective for SP4—and provided peptides ready for LC-MS without any further cleanup required. Therefore, rapid protein aggregate capture by centrifugation-based SP4 provides a potential option for the preparation of samples for proteomics analysis.

### Centrifugation Outperforms Magnetic Capture of Solvent-Induced Protein Aggregates

To evaluate how the capture of protein aggregates by centrifugation compared with magnet- and CMMB-based SP3, 1–5000 μg of HEK293 cell lysate was processed by SP3, SP4-BF, and SP4-GB (Figures 1B and S2 and Tables S2–S6). Both variants of SP4 consistently either matched or exceeded the number of protein and peptide identifications of SP3 across the range of evaluated inputs. A mean of 3036, 3275, 3810, 2549, and 3272 proteins were identified for the 1, 10, 100, 500, and 5000 μg input experiments, respectively. On average, more proteins were observed for the 1, 100, 500, and 5000 μg inputs for SP4-BF (+569 (*p* < 0.05), +129, +172, (*p* < 0.05), and +63 proteins, respectively) and SP4-GB (+506 (*p* < 0.05), +149, +350 (*p* < 0.01), and +114) *vs* SP3, with the 10 μg experiment showing roughly equivalent protein numbers (SP3: 3281; SP4-BF: 3248; and SP4-GB: 3297, Figure 1B and Table S1). Peptide identifications (Figure 1B) and other measures of proteome quality (Figure S2) also consistently indicated greater or equivalent protein recovery by SP4. Quantitative reproducibility was also assessed, with coefficients of variation (CV, Figure 1B) indicating at least equivalent or greater reproducibility for SP4 in the 1, 10, 500, and 5000 μg comparisons. Median protein *R*^2^ values were 0.970, 0.980, and 0.993 for SP3, SP4-BF, and SP4-GB, respectively (Figure S3). For both SP4 methods, more proteins demonstrated significantly greater recovery (fold change (FC) > 2 and adjusted *p* < 0.05) *vs* SP3, with SP4-GB offering additional recovery for all inputs (Figure S4). A slight trend of greater recovery of transmembrane proteins was apparent in these data (Figure S4). The inclusion of glass beads also offered some marginal increases to mean protein identifications *vs* SP4-BF for the 10, 100, 500, and 5000 μg (49, 20, 179 (*p* < 0.01), and 52, respectively), alongside lower CVs in these samples. Missed cleavages were reduced in all but the lowest input (1 μg) for SP4-GB relative to SP3, and for all but the lowest and highest (1 and 5000 μg) inputs relative to SP4-BF (Figure S2).

Next, to evaluate the hypothesis that some proteins were not fully aggregating or captured by CMMBs in SP3, the SP3 protocol was performed with centrifugation in place of magnetic capture (“cSP3”) (Figures 1C and S5). cSP3 outperformed magnetic capture of protein–bead aggregates, with significantly increased protein (+215, *p* < 0.05) and peptide (+1492, *p* < 0.01) identifications.

The previously noted^13^ effects of protein concentration on SP3 and SP4 were also investigated. Recovery from 0.025 *vs* 0.25 μg/μL protein sample concentrations (Figure 1D) indicated that, although the 10-fold dilution caused significant losses in all three workflows, the losses were far greater for SP4-BF (3246 *vs* 669, *p* < 0.0001) and SP4-GB (3184 *vs* 1674, *p* < 0.0001) than for SP3 (2678 *vs* 2135 proteins, *p* < 0.05). Each 2-fold protein dilution indicated an approximate 15 and 20% loss of recovered peptides for SP3 and SP4, respectively (Figure S1C). Our results highlight an important limitation of SP4, with SP3 providing superior recovery for low-concentration samples.

While the advantages of SP4-GB over SP4-BF were generally marginal, the addition of glass beads offered several technical advantages, most notably increasing the visibility, definition, density, and ease of resuspension of the protein pellet (Figure 1E).

Finally, several additional aspects of SP4 were investigated, identifying similar yields using acetone instead of ACN for precipitation (Figure S5B), superior peptide yield at lower centrifugation speeds (Figure S5D), and broad compatibility with alternative, detergent-free lysis approaches such as trifluoroacetic acid in the “Sample Preparation by Easy Extraction and Digestion” (SPEED) protocol^23^ (Figure S5E) and urea (Figure S5F).

Together, these findings suggest that centrifugation-based protein aggregate capture by SP4 offers robust advantages over dependence on CMMB–aggregate interactions of SP3 (except in circumstances where protein concentration is very low) and confirm its compatibility across a broad range of cell lysis and aggregate-capture parameters.

### Deep Proteome Profiling Identifies Superior Recovery of Membrane and Low-Solubility Proteins by SP4

**Figure 2:**
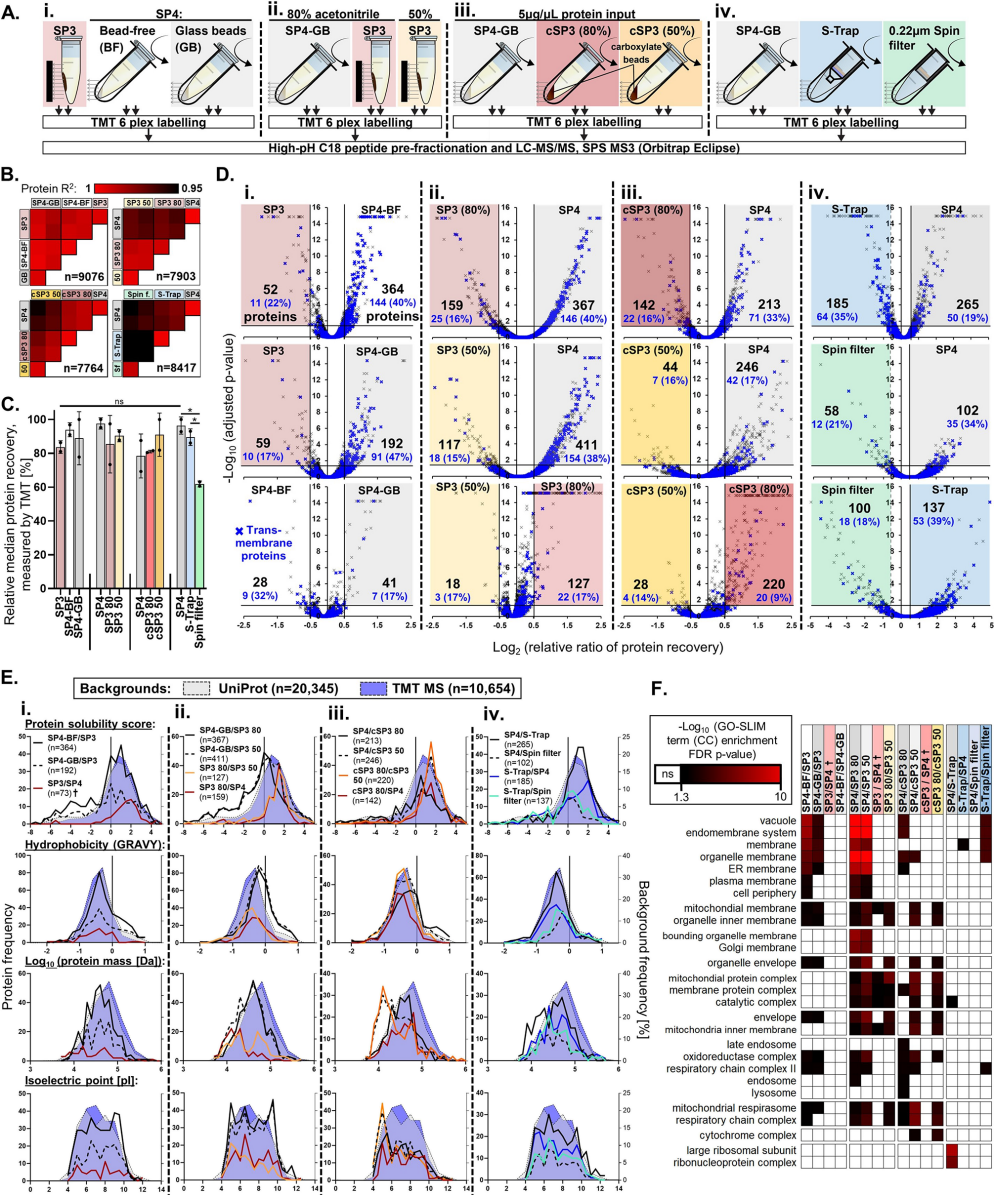
Deep proteome profiling comparing SP3, SP4, and other protein precipitation methods by isobaric labeling. (A) Experimental workflows applied to compare variants of SP3, SP4, and other protein precipitation capture methods to a high depth of proteome coverage. (B) Correlation between protein abundances for sample preparation method replicates. (C) Relative protein recovery percentages determined within each 6-plex across method replicates (*n* = 2) derived from TMT quantitation values. *p < 0.05. (D) Volcano plots indicating more effective protein recovery (adjusted *p* < 0.05 and log_2_ (fold change) > 0.5) by each of the preparation approaches. Blue crosses and numbers represent transmembrane proteins and their proportion of the differentially recovered proteins. (E) Frequency distributions of physical properties among proteins with significantly greater recovery (defined in (D)). Both the human UniProt Swiss-Prot (gray) and the MS-derived TMT (blue) proteomes are displayed as percentage frequency backgrounds. See also Figure S7. (F) Cellular component GO-SLIM term enrichment analysis of proteins more effectively isolated by each method (defined in (D)). ^**†**^Protein lists were combined for these analyses, *e.g*., SP3/SP4 = SP3/SP4-BF and SP3/SP4-GB.

To understand better the nature and mechanisms of proteins not captured by SP3, we next evaluated the proteins recovered by SP3 and SP4 to a higher depth by isobaric labeling and off-line peptide fractionation (Figure 2A-i). Briefly, 100 μg of peptides were prepared in duplicate by SP3, SP4-BF, and SP4-GB, labeled with TMT 6-plex and characterized by two-dimensional (2D) LC-MS/MS using synchronous precursor selection (SPS) and MS^3^ quantification. With this approach, we were able to evaluate quantitatively the recovery of peptides matching 9076 proteins.

Protein recovery had high inter- and intra-method correlations (*R*^2^ > 0.98 and 0.99, respectively) (Figures 2B and S6), with SP4 indicating a marginally higher median protein yield than SP3, as measured by TMT (Figure 2C). Compared with SP3, 364 and 192 proteins had significantly higher recovery (log_2_(FC) > 0.5, *p* < 0.05) for SP4-BF and SP4-GB, respectively (Figure 2D-i). Only 73 proteins had a greater recovery by SP3 *vs* SP4 (BF or GB). Very little differential recovery was observed between the BF and GB SP4 variants (28 and 41 proteins, respectively). The physicochemical properties of differentially recovered proteins highlighted a significant enrichment of hydrophobic and lower-solubility proteins (p < 0.0001) by both SP4 variants *vs* SP3 (Figures 2E-i and S7). Annotation enrichment additionally identified several terms descriptive of membrane proteins for SP4 (Figures 2F and S6), such as “membrane” (*n* = 221/364, *p* = 2.4 × 10^–5^) and “intrinsic component of membrane” (*n* = 153/364, *p* = 7.9 × 10^–14^) (Figure S6). For SP4-BF and SP4-GB, 40 and 47% (144/364 and 91/192) were annotated as transmembrane proteins, respectively (Figure 2D-i, blue crosses)—almost three times the background rate observed by LC-MS (16%).

Given previous suggestions that lower organic conditions be used for SP3-based aggregation,^9^ we compared 50 *vs* 80% ACN for SP3 and SP4-GB (Figure 2A-ii). This experiment reflected the findings of the first 6-plex, with SP4 offering greater differential recovery of proteins (411 and 367 proteins (log_2_(FC) > 0.5, *p* < 0.05), Figure 2D-ii), transmembrane proteins (146 and 154, Figure 2D-ii), hydrophobic proteins (*p* < 0.0001, Figures 2E-ii and S7), and “membrane”-annotated proteins (*p* < 0.0001, Figure 2F-ii) *vs* SP3 using 80 and 50% ACN, respectively. Importantly, these observations for SP4 held true *vs* SP3 at either ACN concentration and were more pronounced when compared to 50% ACN. Losses of low-molecular-weight and soluble proteins were apparent for the use of 50 *vs* 80% ACN for SP3 (*p* < 0.0001, Figures 2E-ii and S7), among those 127 proteins exhibiting significantly lower recovery (log_2_(FC) > 0.5, *p* < 0.05, Figure 2D-ii).

SP3 (80% ACN) demonstrated a greater recovery of lower-than-median molecular weight proteins (52 and 98, *p* < 0.0001) and higher-than-median solubility proteins (56 and 114, *p* < 0.0001) *vs* SP4 in both TMT experiments (Figure 2E-i,E-ii, respectively). However, generally, higher numbers of lower-than-median solubility proteins (208, 116, and 255, *p* < 0.0001) and transmembrane proteins (144, 91, and 146) had greater recovery for SP4-BF, SP4-GB (TMT-i) and SP4-GB (TMT-ii), respectively, *vs* SP3 (Figure 2D,E).

To determine whether lower membrane protein yields in SP3 resulted from fragile aggregates being lost during magnetic capture, SP4-GB was compared with centrifugal SP3 (cSP3) in a third TMT 6-plex, again using both 80 and 50% ACN for SP3. This experiment also offered insight into the impact of CMMB presence during precipitation, independent of the capture method (magnetic or centrifugal). As CMMBs appeared to offer an increased concentration of surrogate nucleation points (Figures 1D and S1C), we attempted to minimize this effect using a high concentration of protein (5 μg/μL)—theoretically providing ample nucleation points across all three conditions.

cSP3 with 80% ACN matched SP4 in most measures, with consistent median recovery (Figure 2C) and reproducibility (*R*^2^ = 0.9966 (cSP3-80%) *vs* 0.9941 (SP4-GB)) (Figure 2B) and balanced differential recovery (142 (cSP3-80%) *vs* 213 (SP4-GB) proteins) between the methods (Figure 2D-iii). Less than half the number of membrane proteins exhibited losses for cSP3-80% (*n* = 71) (Figure 2D-iii) *vs* magnetic SP3 (*n* = 146) (Figure 2D-ii), although some enrichment for SP4-GB remained *vs* cSP3.

The use of 50% ACN with cSP3 also presented greater losses of specific proteins *vs* 80% ACN (*n* = 220, log_2_(FC) > 0.5, *p* < 0.05, Figure 2D-iii), especially those with lower isoelectric points and molecular weights (*p* < 0.0001, Figures 2E-iii and S7). For all comparisons to SP3, cSP3, and SP4 (all using 80% ACN), the use of 50% ACN resulted in the less efficient capture of low-molecular-weight and high-solubility proteins (Figure 2D-ii,D-iii). It is worth noting that cSP3-50% indicated a marginally higher median total protein yield (based on summed intensities of all TMT quantitations) relative to SP3 and SP4 using 80% ACN (Figure 2C), but this did not translate to a greater recovery of many specific proteins (*n* = 28, log_2_(FC) > 0.5, *p* < 0.05, Figure 2D-iii).

Taken together, our analysis indicates that centrifugation offers a more effective means of aggregate capture than magnetism, especially among membrane and other low-solubility proteins. When protein input and concentration are sufficient and centrifugation is an option, CMMBs can be omitted during aggregate capture in many applications.

### SP4 Matches the Performance of S-Trap

To understand the performance of SP4 versus other protein cleanup methods, a further fractionated 6-plex (Figure 2A-iv) was employed—alongside a label-free analysis (Figure S9, *n* = 4)—to compare the deep proteome (*n* = 8417) recoveries of protein precipitate captured by SP4 *vs* two filtration-based aggregate-capture approaches: S-Trap, and 0.22 μm spin filters.^22,23^ SP4 matched S-Trap in most measures, with 265 *vs* 185 (50 *vs* 64 transmembrane) proteins, respectively, exhibiting significantly higher recovery (log_2_(FC) > 0.5, *p* < 0.05, Figure 2D-iv), consistent reproducibility (*R*^2^ = 0.9970 *vs* 0.9964, Figure 2B), and a marginally higher median recovery for SP4 (Figure 2C). For S-Trap *vs* SP4, protein property distributions were skewed toward trends of higher recovery for high-solubility proteins, lower recovery of low-molecular-weight proteins (Figure 2E-iv), and significantly lower recovery of “ribonucleoproteins” (*n* = 21/265, *p* = 2.0 × 10^–7^, Figures 2F and S8). For label-free, SP4 identified significantly more peptides than S-Trap (*p* < 0.05) but offered lower CVs% (Figure S9). Spin filters exhibited significantly lower recovery (<70% of SP4 or S-Trap, *p* < 0.05) and reproducibility across both the TMT and label-free experiments (Figures 2B,C and S9). Overall, SP4 and S-Trap appear to provide broadly similar results, whereas the use of spin filters risks losses.

### SP4 Matches or Outperforms SP3 Independent of User and Sample Type

**Figure 3:**
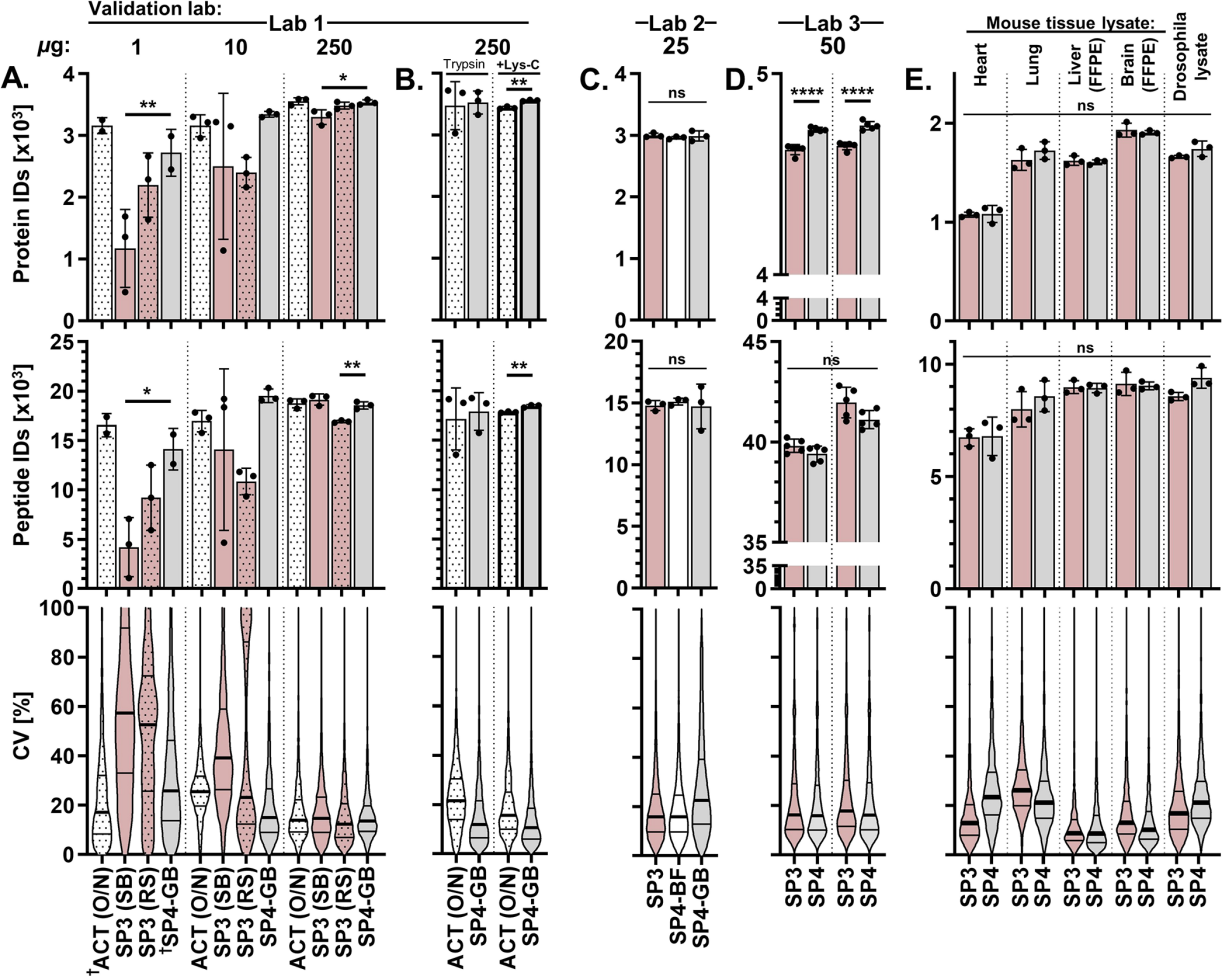
Independent method validations and complex applications of SP4 cleanup for proteomics. The SP4 protocol was provided to three collaborators and applied to several sample types to compare SP4 with SP3. (A) Lab 1 performed SP3 with either SpeedBeads carboxylate or ReSyn HILIC magnetic beads compared with overnight acetone (ACT(O/N)) precipitation and SP4-GB for 1, 10, and 250 μg preparations of Jurkat human immortalized T cell lysate (*n* = 3). †*n* = 2; see Supporting Methods. (B) Acetone precipitation and SP4 were compared for 250 μg HEK293 lysate digested with trypsin +/− Lys-C. (C) Lab 2 processed 25 μg of HEK293 lysate for SP3, SP4-BF, and SP4-GB protocols. (D) Lab 3 processed two independent *n* = 5 comparisons of SP3 and SP4-GB using 50 μg of E14 murine embryonic stem cell lysate. (E) SP3 and SP4 preparations of more complex lysates/homogenates derived from whole organs, organisms, and formalin-fixed paraffin-embedded (FFPE) tissue. Bar charts present median and standard deviation, with significance assessed by ANOVA (A, C) and *t*-test (B, D, E). Protein coefficients of variance distributions represented by violin plot (thick line–median, thin lines–quartiles). *p < 0.05, **p < 0.01, ***p < 0.001, ****p < 0.0001, and ns–not significant.

To confirm that SP4 was not dependent on any single user, setting, or sample complexity, the protocol was shared with three collaborators and applied to lysates from several sources (Figure 3). Lab 1 found that SP4-GB consistently performed effectively across a range of protein inputs, matching or outperforming SP3 with two magnetic particles (ReSyn (RS) HILIC or SpeedBeads (SB) carboxylate beads) and overnight acetone precipitation (Figure 3A), especially when additionally digesting with Lys-C (Figure 3B). Lab 2 prepared 25 μg of HEK293 lysate in triplicate and found SP3, SP4-BF, and SP4-GB roughly equivalent (Figure 3C). Lab 3 compared SP3 and SP4-GB with two independent (*n* = 5) comparisons of 50 μg of mouse E14 embryonic stem cell lysate. For both experiments, approximately 100 more proteins were identified by SP4-GB (*p* < 0.001), even though the number of peptides did not significantly differ between comparisons (*p* > 0.05, Figure 3D). We also performed SP4 *vs* SP3 on more complex samples, including lysates derived from whole mouse organs, formalin-fixed paraffin-embedded (FFPE) tissue preparations, and whole *Drosophila melanogaster*, to confirm the broad utility of SP4 (Figure 3E,F). Importantly, no significant differences were observed between the two methods (*p* > 0.05). These experiments further demonstrate that the SP4 protocol consistently either matches or outperforms SP3 independent of user, setting, or application.

## Discussion

SP3 is one of the most effective means of proteomics sample capture and cleanup currently available. However, its reliance on stable aggregation of proteins onto magnetic beads remains a potential source of variability and loss. By evaluating centrifugation of protein aggregates with SP4—with or without glass beads—we show that losses exhibited by SP3 can be reduced and that CMMBs are not required for effective protein aggregate capture for many applications. SP4 robustly offered greater or equivalent protein and peptide identifications *vs* SP3 across a broad range of conditions, including 1–5000 μg of protein input, eight sample types, five lysis buffers, and four lab settings that use a diverse range of downstream proteomics methods.

Generally, SP3 and SP4 provided highly comparable proteomics options, with both offering a rapid single-pot protein capture and cleanup protocol, broad compatibility, and the option to elute LC-MS-ready peptides. Each method, however, offered different advantages for protein cleanup. While SP3 performed better at very low protein concentrations (*e.g*., 0.025 μg/μL, Figure 1F) and for a subset of low-molecular-weight proteins (Figure 2E-ii.), SP4 matched or outperformed SP3 at the higher concentrations used in this study (0.25–5 μg/μL)—especially among proteins with low solubility, high hydrophobicity, and transmembrane domains (Figure 2D–F). Additionally, SP4 requires no specialized reagents or equipment, allows rapid preparations with or without beads, and offers low-cost, high-input scalability to preparations beyond the recommended 300 μg limit for SP3.^9^ SP4 therefore provides a more robust and effective means of protein cleanup for global proteomics studies compared to SP3, especially when a high protein concentration is available (>0.25 μg/μL) and marginal losses to some smaller, soluble proteins are tolerable.

In most instances, glass beads provided some (albeit limited) improvement to proteomics outputs *vs* SP4-BF; however, their most notable advantages were technical. Glass beads outcompeted tube walls as a precipitation surface, promoted a more defined, visible, and stable precipitation pellet (Figure 1E), facilitated pellet resuspension, and offered fewer missed cleavages (Figure S2). They also present greater chemical and freezing compatibility and substantially lower cost (∼1/1000th) than CMMBs. When tested, SP4-GB was also found to be compatible with 2 h digestions (Figures 2A-iv and 3D). Presuspending the glass beads in ACN prior to sample addition improved reproducibility and avoided dilution from aqueous bead slurries. Glass beads therefore offer clear advantages over SP4-BF and may offer benefits for other protein precipitation approaches.

Where SP4 outperformed SP3, the use of centrifugation appears to have mitigated losses arising from dependence on effective magnetic capture of protein–bead aggregation. Aggregation-resistant proteins and fragile aggregates prone to mechanical disruption would risk removal with the supernatant and washes. This likely explains, alongside improved reproducibility, the greater recovery by SP4 (and cSP3) of hydrophobic and lower-solubility proteins—which exhibit a reduced propensity for organic solvent-induced aggregation.^34^ Interestingly, some marginal losses to membrane proteins remained during cSP3 (Figure 2D-iii,E-iii), suggesting either superior glass bead binding of hydrophobic proteins or incomplete elution of hydrophobic peptides from CMMBs. The higher recovery of low-molecular-weight proteins by SP3 does suggest that carboxylate chemistry may facilitate the capture of some peptides which are less prone to precipitation.^30,32^

At high protein concentrations, SP4 and SP3 yielded consistent recovery across the majority of the proteome—adding to suggestions that protein precipitation is the primary mechanism of SP3.^8,13^ Protein–protein and protein–CMMB aggregation both likely derive from highly similar electrostatic interactions of protein elements exposed by dehydration and denaturation. This may explain the paradoxical losses observed at higher protein inputs and concentrations for SP3^5,11^ (Figures 1B and S1C) if protein–CMMB aggregation is outcompeted by protein–protein aggregation, resulting in particles that are not captured by magnetism. Conversely, at lower protein concentrations, where nucleation points are scarce, the rapid nature of denaturation-induced aggregation—often termed a protein “crash”—drives finer precipitate formation and tube-wall adhesion and perhaps explains the low yield observed for SP4-BF (Figure 1D). CMMBs therefore appear to alleviate the scarcity of protein–protein interaction sites at lower concentrations by providing additional electrostatic nucleation points, thereby expediting more stable precipitation. HILIC-type interactions may also play a role in this process. Although glass beads also ameliorated bead-free losses, their effect was less pronounced, perhaps due to the lack of additional electrostatic nucleation and reliance on hydrophobic interactions alone, which are weaker in nature and thus may proceed more slowly. Therefore, while protein precipitation appears to be the primary mechanism of protein capture for both SP3 and SP4, CMMB and GB physicochemical properties may offer some mechanistic divergence in the role they provide as nucleation points, driving initial aggregate capture more prevalently through electrostatic and hydrophobic interactions, respectively.

A precipitation mechanism also has implications for organic solvent concentration selection, where higher percentages offer greater denaturation. This was apparent among the consistently lower recovery of many proteins observed with the use of 50% ACN for both SP3 and cSP3 *vs* 80%, most notably for low-molecular-weight proteins. However, there was a marginal signature of higher global median protein yield (Figure 2C, also noted in Figure S1), likely arising from the lower and thus more concentrated aggregation reaction volume. This indicates a trade-off between the improved recovery of subsets of hydrophobic and low-molecular-weight proteins (80% ACN) and marginally higher global yields (50% ACN). The role of protein precipitation in SP3 also suggests that ionic strength, like with SP4, should be carefully considered during aggregation.^25^

SP4-GB broadly matched S-Trap, offering marginally higher yields (Figures 2C,D-iv and S9)—perhaps resulting from losses on the additional surfaces presented by the S-Trap protocol. S-Trap had lower variability for label-free samples (Figure S9) but not for the TMT samples (Figure 2-iv). Notably, SP4 eschews the specialist devices, multiple elution steps, peptide concentration steps, multiple vessels, and buffer restrictions of S-Trap. Importantly, our presentation of a common mechanistic bridge between SP3 and other protein precipitation-based methods such as S-Trap, ProTrap-XG, and filter-aided SPEED offers several potential avenues for further optimization and cross-adaptation of existing best practices.

Alongside limitations at low protein concentrations and the loss of some low-molecular-weight proteins,^30,32^ SP4 does not benefit from certain advantages offered by CMMBs, *e.g*., the options to enrich peptides or adapt for high throughput and automation^8,14−16^ (although we note that SP4 was compatible with lower centrifugation speeds more typically employed for 96-well plates (Figure S5D)).

SP4 undoubtedly has the potential for further optimization. For example, the precipitation step could be enhanced by cold temperatures, carefully titrated ACN concentrations, and longer centrifugation at slower speeds. The trade-off between a denser aggregate pellet and the ease of resuspension for trypsin accessibility may be worthy of further exploration (Figure S5D), although Lys-C, rapid digestion buffers, and higher digestions temperatures appear to be effective solutions (Figures 2 and 3D). The type of bead is also worthy of exploration, such as size, material, and surface chemistry. Cheaper, non-magnetic carboxylate-modified beads used alongside centrifugation and washes, like cSP3, might offer benefits of both approaches.

## Concluding Remarks

SP4 addresses key limitations of SP3 with the use of centrifugation and glass beads, providing a minimalistic, low-cost protein cleanup method that offers greater or equivalent protein yields when protein concentration and input are sufficient. SP4 is particularly applicable to the preparation of high-input samples (*e.g*., for PTM preparations) and for biology labs with limited proteomics experience and preparation equipment. We provide further evidence that precipitation is the primary mechanism of SP3 cleanup and that CMMBs can be omitted from high-concentration protein capture in many applications. We hope these findings will extend options, improve understanding, and encourage further development of proteomics sample cleanup methods.

## Supporting Information Available

Expanded results, analysis, optimization work, and additional exploratory experiments (Figures S1–S9); supporting methods; supporting methods for validation work; detailed step-by-step protocol for SP4-BF or SP4-GB sample preparation (SP4 protocol); detailed tables and summaries of the proteomics findings (Tables S1–S20) (PDF)
(XLSX)
