# Supplementary material for: Solvent Precipitation SP3 (SP4) Enhances Recovery for Proteomics Sample Preparation without Magnetic Beads
Source: Anal Chem. 2022 Jul 18;94(29):10320–8. doi: 10.1021/acs.analchem.1c04200 (PMC9330274; doi:10.1021/acs.analchem.1c04200)
Supplement: Supplementary file 1 — ac1c04200_si_001.pdf [file ac1c04200_si_001.pdf]

# Supporting Information

# Solvent Precipitation SP3 (SP4) enhances recovery for proteomics sample preparation without magnetic beads

AUTHORS

Harvey E. Johnston[1]*, Kranthikumar Yadav[2], Joanna M. Kirkpatrick[3], George S. Biggs[3,4], David Oxley[2], Holger B. Kramer[5], Rahul S. Samant[1]*

1. Signalling Programme, The Babraham Institute, Cambridge, CB22 3AT, United Kingdom
2. Mass Spectrometry Facility, The Babraham Institute, Cambridge, CB22 3AT, United Kingdom
3. Proteomics STP, The Francis Crick Institute, London, NW1 1AT, United Kingdom
4. GlaxoSmithKline, Gunnels Wood Road, Stevenage, Hertfordshire, SG1 2NY, United Kingdom
5. Medical Research Council London Institute of Medical Sciences, Imperial College London, Hammersmith Hospital, London, W12 0NN, United Kingdom

*Corresponding authors: harvey.johnston@babraham.ac.uk; rahul.samant@babraham.ac.uk

**Supplementary Figures S1 – S9………………….…………………………Pages S3 – S15**

**Figure S1.** Evaluation of a range of SP4-related variables by peptide quantitation assay (*n* = 4) and proteomics analysis.

**Figure S2.** Additional measures of proteome quality and protein recovery for the comparison of SP3 to SP4 with (GB) and without glass beads (bead-free, BF) across a range of protein inputs (Fig 1B).

**Figure S3.** Protein and peptide LFQ $R^2$ values of recovery for SP3 to SP4, with (GB) and without glass beads (bead-free, BF), across the range of evaluated protein inputs (Fig 1B).

**Figure S4.** Protein recovery observed to be more effective by SP4 variants *vs* SP3, summarized in Fig 1B.

**Figure S5.** Additional experiments exploring the mechanism and potential of bead-free (BF) and glass bead (GB) SP4.

**Figure S6.** Additional measures of quantitative proteome quality for the comparison of SP3 to SP4 with and without glass beads using TMT 6-plex and SPS $MS^3$, summarized in Fig 2.

**Figure S7** Frequency distributions of physicochemical properties among proteins with significantly greater recovery observed by the TMT experiments (defined in Fig 2D).

**Figure S8.** DAVID-derived term enrichment and clustering for those proteins observed more significantly recovered by SP3 and SP4 by TMT quantitation.

**Figure S9.** A label-free comparison of proteomics preparations by SP4, S-Trap, and precipitate capture by 0.22 $\mu$m nylon spin filters.

**Supplementary Methods……………………………………………………..Pages S16 – S19**

**Supplementary Methods for SP4 validation………………….………….Pages S20 – S23**

**Table S21.** Summary of key methods used by the validation labs.

**SP4 protocol…………………………………………………………………Pages S24 – S26**

Additional information:

**Table S1 – S20 (.xlsx spreadsheet)** Detailed tables and summaries of the proteomics findings. All values are unnormalized to demonstrate technical effects on recovery.

**Table S1.** Key measures of proteome quality outputs from label-free comparisons of SP3 with bead-free (BF) and glass bead (GB) SP4 (Summarizing **Tables S2 – S8** and illustrated in **Fig 1B and S2**)

**Table S2.** 1 $\mu$g (of protein) SP3 *vs* SP4-BF *vs* SP4-GB preparation proteomics (**Fig 1B**)

**Table S3.** 10 $\mu$g (of protein) SP3 *vs* SP4-BF *vs* SP4-GB preparation proteomics (**Fig 1B**)

**Table S4.** 100 $\mu$g (of protein) SP3 *vs* SP4-BF *vs* SP4-GB preparation proteomics (**Fig 1B**)

**Table S5.** 500 $\mu$g (of protein) SP3 *vs* SP4-BF *vs* SP4-GB preparation proteomics (**Fig 1B**)

**Table S6.** 5000 $\mu$g (of protein) SP3 *vs* SP4-BF *vs* SP4-GB preparation proteomics (**Fig 1B**)

**Table S7.** 10 $\mu$g optimization experiments, exploring centrifugation speed, bead:protein ratio, ACN concentration, use of centrifugation with SP3 beads (cSP3), and the application of the SPEED method, summarized in **Fig S1** and **S5**

**Table S8.** 500 $\mu$g prepared using 8 M urea as the lysis buffer, requiring the dilution of samples to 2 M urea prior to SP3/SP4, summarized in **Fig S5**

**Table S9.** TMT 6-plex of 100 $\mu$g processed by SP3 ($n$ = 2), SP4-BF ($n$ = 2), and SP4-GB ($n$ = 2)

**Table S10.** TMT 6-plex of 100 $\mu$g processed by SP4-GB ($n$ = 2), SP3 with 80% ACN ($n$ = 2), and SP3 with 50% ACN ($n$ = 2)

**Table S11.** TMT 6-plex of 100 $\mu$g processed by SP4-GB ($n$ = 2), centrifugal SP3 (cSP3) with 80% ACN ($n$ = 2), and cSP3 with 50% ACN ($n$ = 2)

**Table S12.** TMT 6-plex of 100 $\mu$g processed by SP4-GB ($n$ = 2), S-Trap ($n$ = 2), and 0.2 $\mu$m spin filters ($n$ = 2)

**Table S13.** 100 $\mu$g processed by SP4-GB ($n$ = 4), S-Trap ($n$ = 4), and 0.2 $\mu$m spin filters ($n$ = 4) by LFQ

**Table S14.** 1 $\mu$g processed at a protein concentration of 0.025 $\mu$g/$\mu$L by SP3 *vs* SP4-BF *vs* SP4-GB preparation proteomics (**Fig 1D**)

**Table S15.** 500 $\mu$g SP3, SP4-BF, and SP4-GB using acetone (ACT) and acetonitrile (ACN), summarized in **Fig S5**

**Table S16.** 20 $\mu$g mouse heart lysate processed by SP3 and SP4-GB ($n$ = 3)

**Table S17.** 20 $\mu$g mouse lung lysate processed by SP3 and SP4-GB ($n$ = 3)

**Table S18.** 20 $\mu$g mouse liver FFPE tissue lysate processed by SP3 and SP4-GB ($n$ = 3)

**Table S19.** 20 $\mu$g mouse brain FFPE tissue lysate processed by SP3 and SP4-GB ($n$ = 3)

**Table S20.** 20 $\mu$g whole *Drosophila* homogenate processed by SP3 and SP4-GB ($n$ = 3)

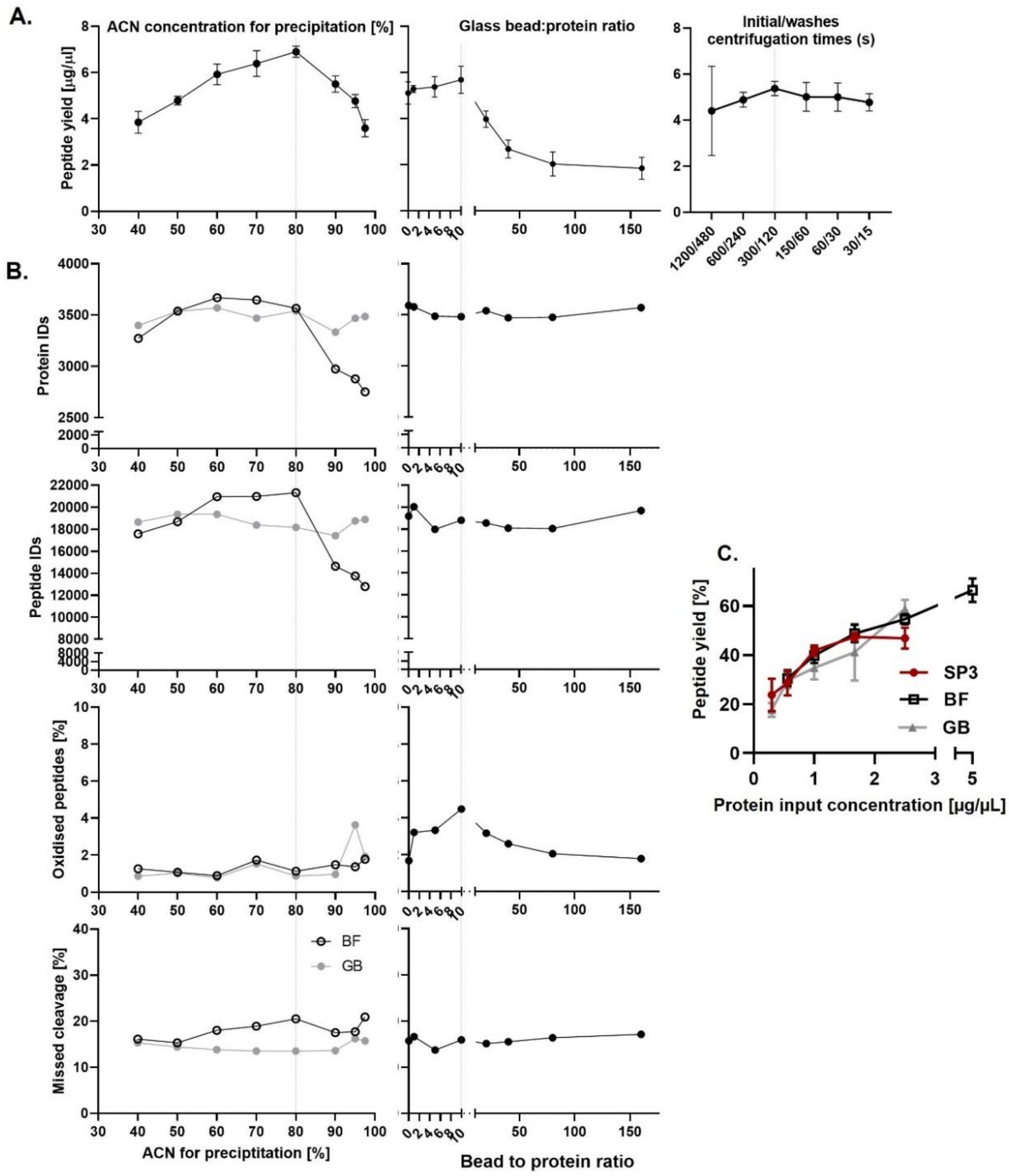


**Figure S1**. **Evaluation of a range of SP4-related variables by peptide quantitation assay (*n* = 4) and proteomics analysis. A.** 10 µg of protein was processed by SP4 varying the initial and post-wash precipitate capture centrifugation times, the glass bead to protein ratio, and the total final percentage of ACN in the precipitation step. The digests were measured by peptide quantitation assay. **B.** For proteomics analyses, 10 µg SP4 sample preparations were evaluated varying bead input and ACN concentration with 100 ng equivalent of peptides analysed by LC-MS. Other variables were kept at either 300/120 s capture/wash centrifugation steps, 10:1 glass bead to protein ratio and 80% ACN. **C.** 50 µg of protein was processed by SP3, SP4-BF and SP4-GB methods across a range of protein concentrations representative of that of the final volume, including the volume from the bead suspension for SP3 and SP4-GB. It was therefore possible to evaluate SP4-BF at twice the protein concentration, with no bead addition required. The samples were subjected to SP3 and SP4 protocols, digested with trypsin in 20 mM ammonium bicarbonate (ABC) and the resulting peptides were measured by peptide assay.

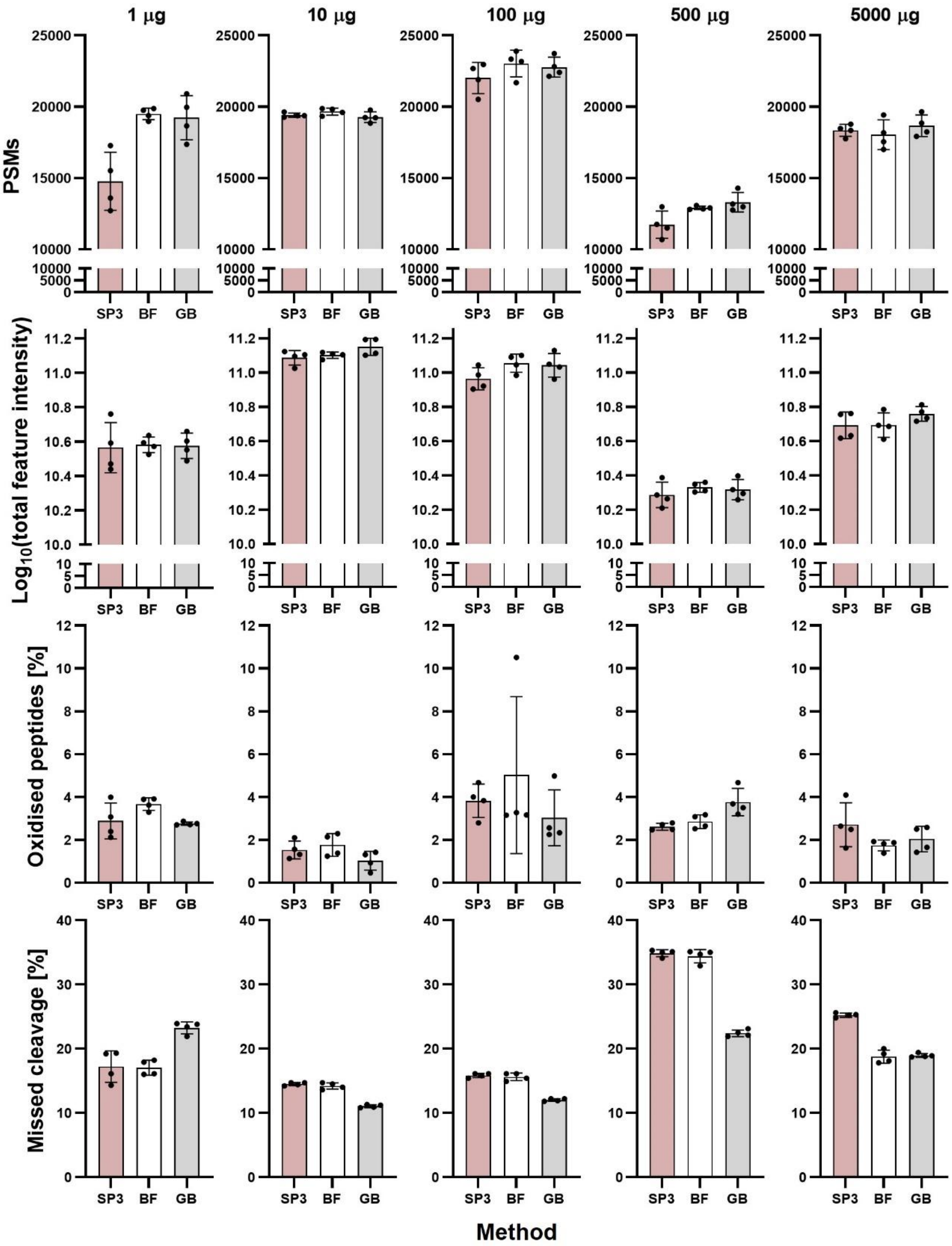


**Figure S2. Additional measure of proteome quality and protein recovery for the comparison of SP3 to SP4 with (GB) and without glass beads (bead-free, BF) across the range of evaluated protein inputs (see also Fig 1B).** PSM = peptide spectrum match.

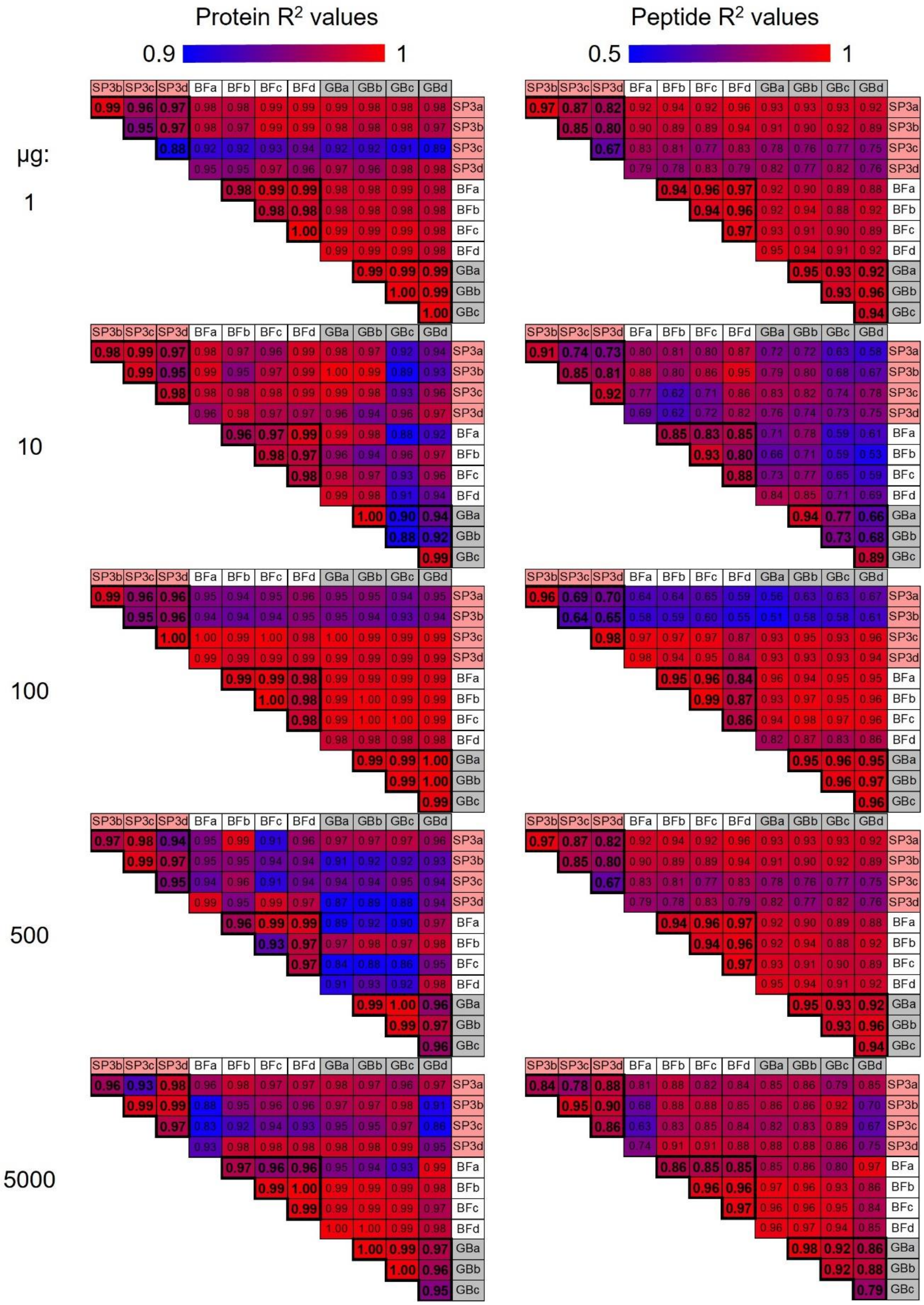


**Figure S3. Protein and peptide LFQ $R^2$ values of recovery for SP3 to SP4, with (GB) and without glass beads (bead-free, BF), across the range of evaluated protein inputs (Fig 1B).**

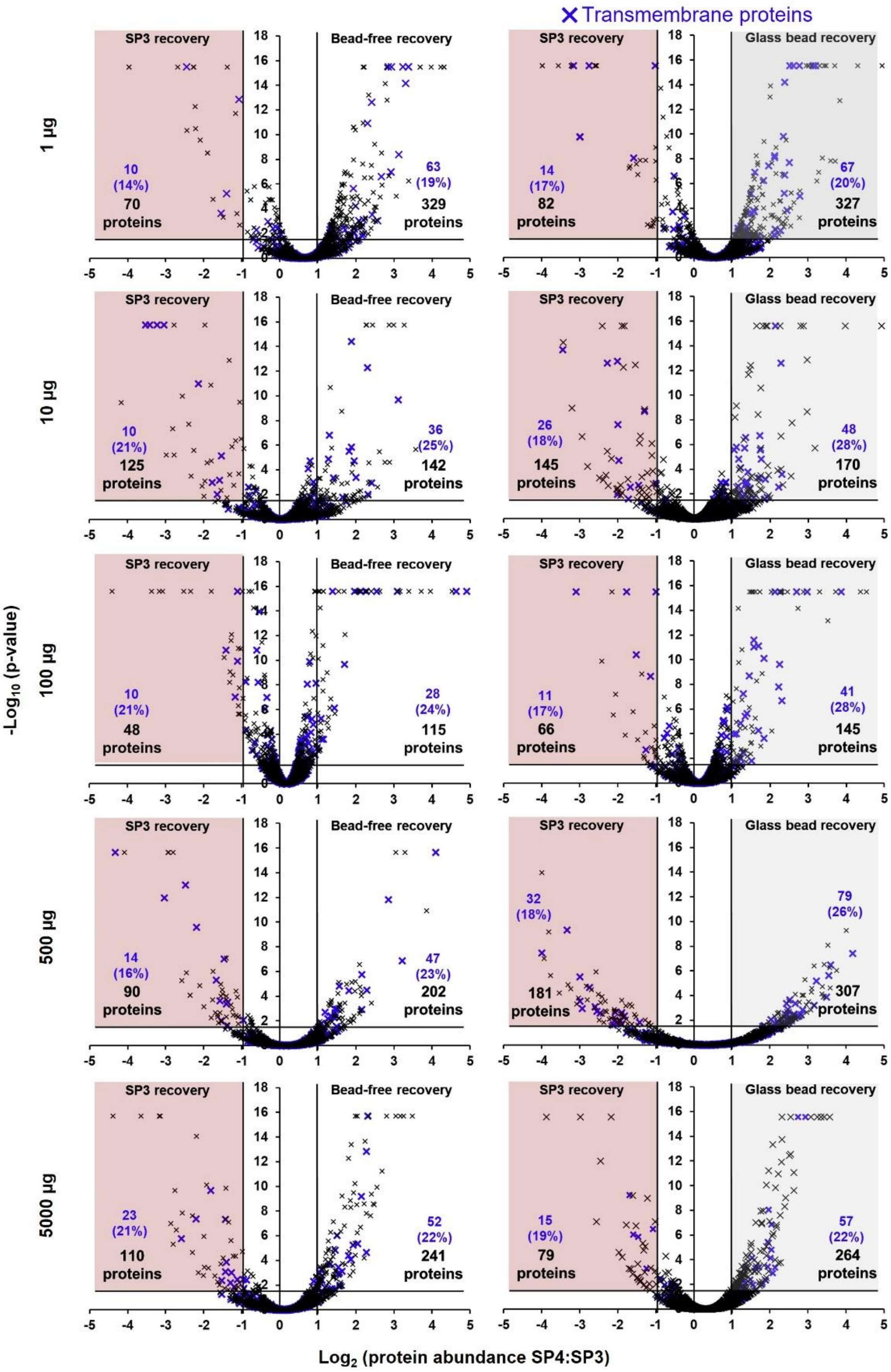


**Figure S4**. **Protein recovery observed to be significantly more effective by SP4 variants *vs* SP3, summarized in Fig 1B.** *p*-values determined by Proteome Discoverer with multiple-test adjusted *t*-test. Blue crosses and numbers denote transmembrane proteins, with the proportion of the significantly differentially recovery proteins ($\log_2$(FC) > 1, $p < 0.05$) annotated as transmembrane proteins in brackets.

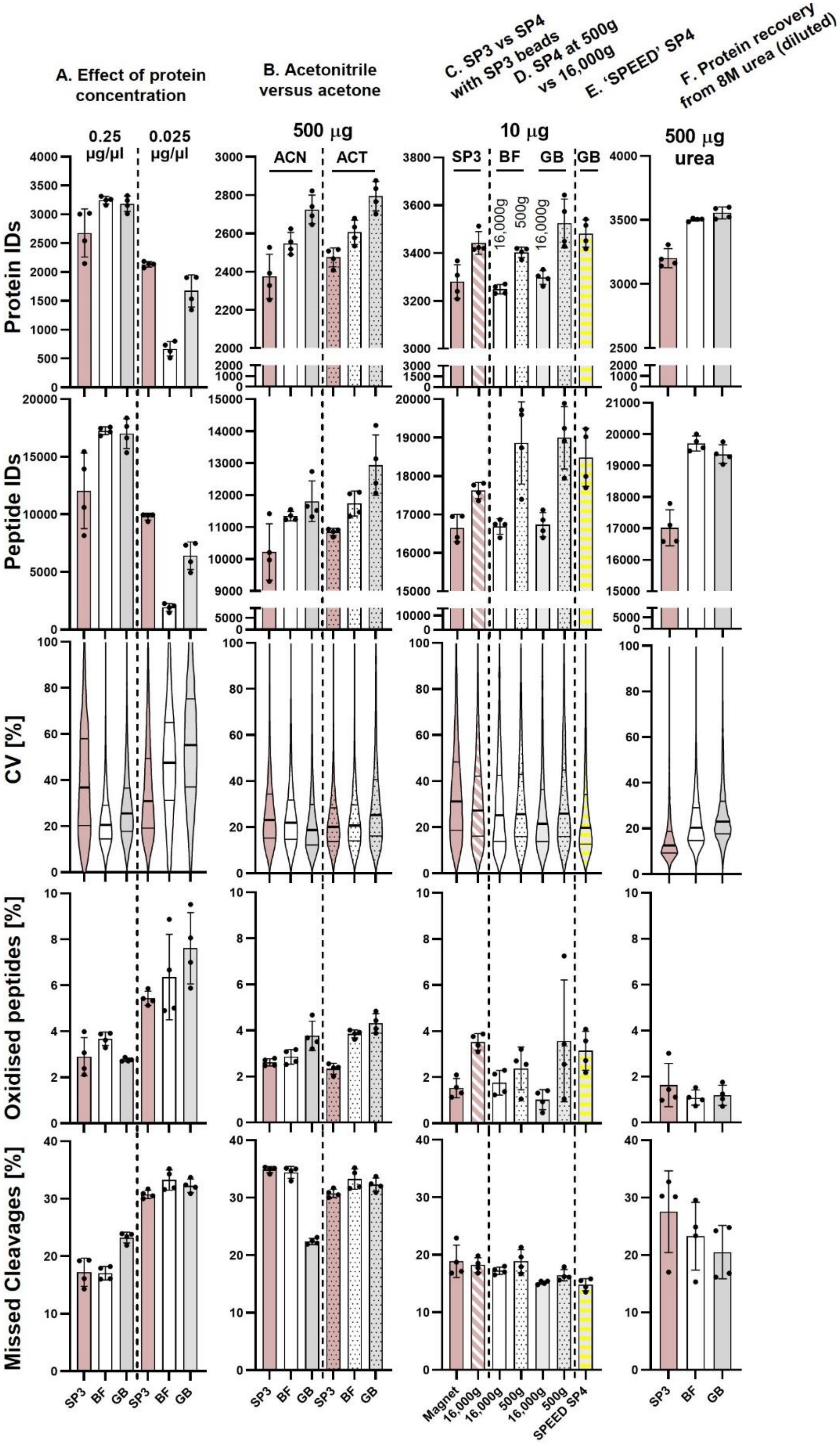

A. Effect of protein concentration
B. Acetonitrile versus acetone
C. SP3 vs SP4 with SP3 beads
D. SP4 at 500g vs 16,000g
E. 'SPEED' SP4
F. Protein recovery from 8M urea (diluted)
0.25 µg/µl
0.025 µg/µl
500 µg
ACN
ACT
10 µg
SP3
BF
GB
GB
16,000g
500g
16,000g
500 µg urea
Protein IDs
Peptide IDs
CV [%]
Oxidised peptides [%]
Missed Cleavages [%]
SP3
BF
GB
SP3
BF
GB
Magnet
16,000g
16,000g
500g
16,000g
500g
SPEED SP4

**Figure S5**. **Additional experiments exploring the mechanism and potential of bead-free (BF) and glass bead (GB) SP4.** Protein numbers, peptide numbers, peptide CVs, oxidised peptides, and missed cleavage rates among peptide spectrum matches are detailed for the 4 procedural replicates. **A.** 1 *μ*g SP3 and SP4 preparations were conducted using two initial protein concentrations: 250 ng/*μ*L (1 *μ*g in 4 *μ*L volume, including beads) and 25 ng/*μ*L (1 *μ*g in 20 *μ*L volume, including beads). **B.** 500 *μ*g SP3 or SP4 preparations were conducted using acetonitrile (ACN) or acetone (ACT) as the denaturing solvent. **C.** SP3 was compared with SP4 using SP3 carboxylate magnetic beads to confirm that centrifugation recovered more protein than the use of a magnet. **D.** BF and GB SP4 variants were tested at 500*g* (vs. 16,000*g* adopted in all other experiments) for the potential to expand their compatibility with larger volume and plate-based preparations. **E.** Cells were lysed by 'SPEED' (Sample Preparation by Easy Extraction and Digestion) method using 100% TFA and neutralised with Tris base before being subjected to SP4 with the inclusion of glass beads. **F.** 500 *μ*g of protein was processed by SP3 to SP4 using 8 M urea as the lysis buffer, across a range of measures of protein recovery and proteome quality. Lysate was diluted to 2 M urea prior to addition of beads and ACN.

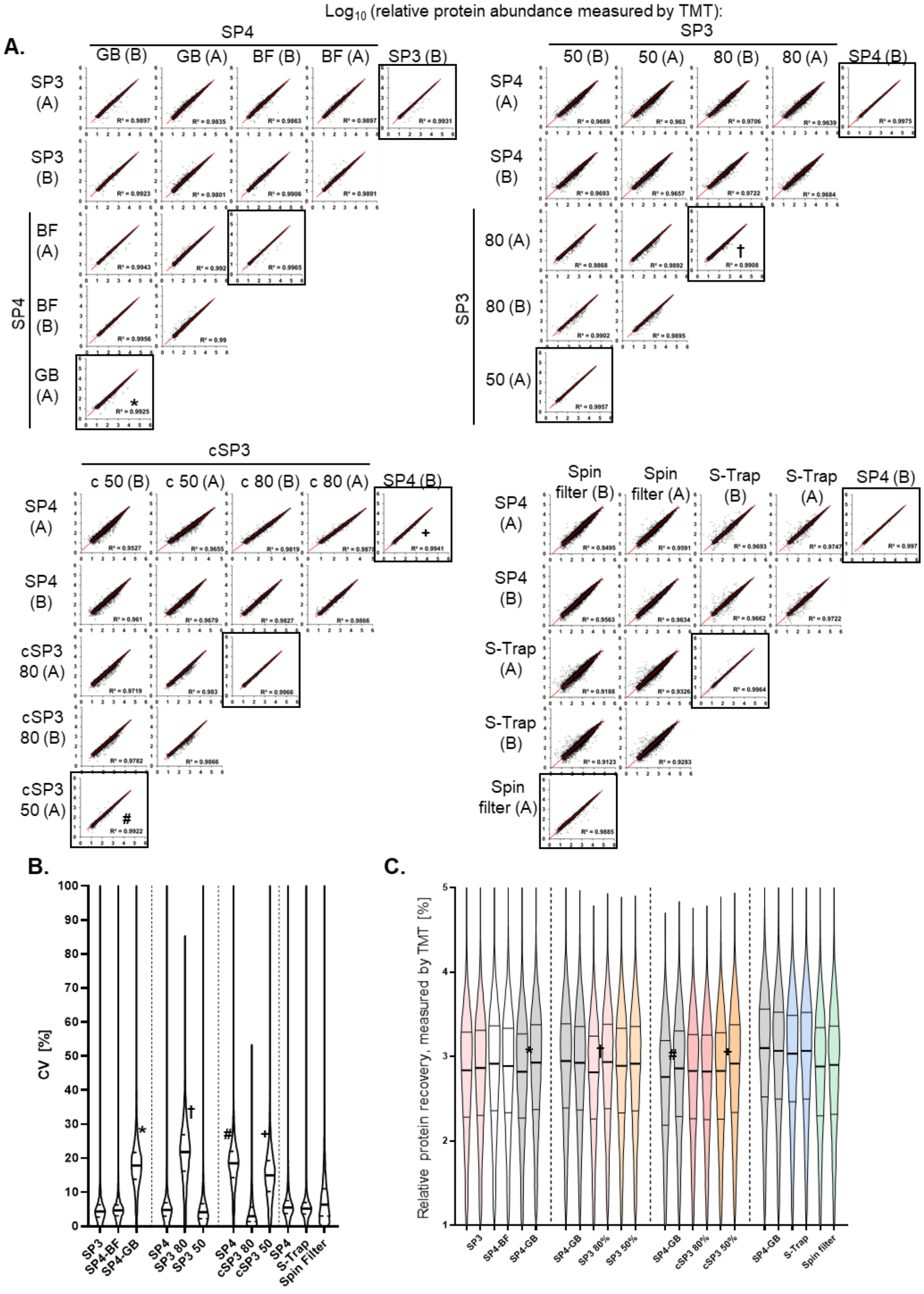


**Figure S6. Measures of quantitative proteome reproducibility for comparison of SP3 and SP4 using TMT 6-plex, summarized in Figure 2.** Correlations between TMT-measured protein abundances for sample preparations and method replicates (**A.**) and coefficients of variation (CV) % (**B.**) and total protein abundance value distributions (**C.**). Data are not normalized to enable full assessment of technical variations (correlation is therefore a better measure of consistency in this instance). Boxes denote the direct correlations between methodological replicates. Those replicates with higher CV% are footnoted *, †, #, and + to their corresponding $R^2$ values, demonstrating that, despite differential total recovery, this was consistent across the whole proteome and did not indicate differential protein loss, with no reduction of relative correlation.

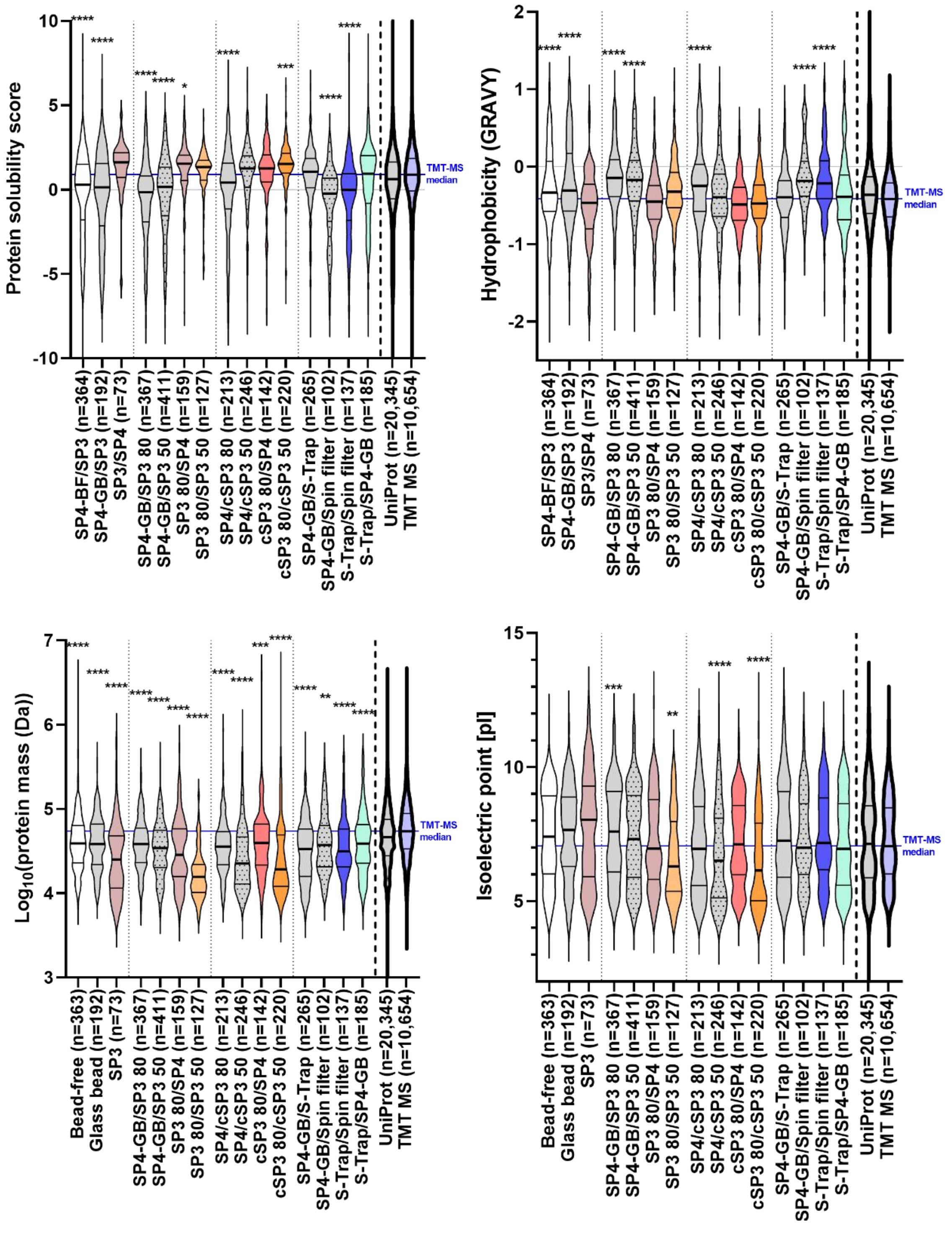


**Figure S7. Frequency distributions of physical properties among proteins with significantly greater recovery (defined in Fig 2D).** Both the human UniProt Swissprot (grey) and the MS-derived TMT (blue) proteomes are displayed as percentage frequency backgrounds. ANOVA followed by Dunnett's multiple comparisons test, compared to the TMT proteome-identified proteins, was used to assess significant deviation from an expected background distribution. Protein coefficients of variance distributions represented by violin plot (thick line–median, thin lines–quartiles). **p* < 0.05, ***p* < 0.01, ****p* < 0.001, *****p* < 0.0001, and ns–not significant.

# Figure S8 – TMT i.

### Functional annotation clustering:

### Functional annotation terms:

‘Bead-free’ proteins (n=364)

| Annotation Cluster 1 | Enrichment Score: 7.92 | | Count | P_Value | Fold Change | Benjamini |
|---|---|---|---|---|---|---|
| UP_SEQ_FEATURE | transmembrane region | RT | 143 | 1.4E-9 | 1.6E0 | 1.9E-6 |
| UP_KEYWORDS | Transmembrane helix | RT | 150 | 7.9E-9 | 1.5E0 | 8.2E-7 |
| UP_KEYWORDS | Transmembrane | RT | 150 | 9.9E-9 | 1.5E0 | 8.2E-7 |
| GOTERM_CC_DIRECT | integral component of membrane | RT | 147 | 3.4E-8 | 1.5E0 | 3.9E-6 |
| UP_KEYWORDS | Membrane | RT | 182 | 6.9E-8 | 1.4E0 | 3.3E-6 |
| **Annotation Cluster 2** | **Enrichment Score: 6.36** | | **Count** | **P_Value** | **Fold Change** | **Benjamini** |
| KEGG_PATHWAY | Oxidative phosphorylation | RT | 18 | 8.8E-10 | 6.7E0 | 1.7E-7 |
| GOTERM_CC_DIRECT | mitochondrial inner membrane | RT | 31 | 2.1E-9 | 3.7E0 | 5.0E-7 |
| GOTERM_CC_DIRECT | mitochondrion | RT | 59 | 2.8E-9 | 2.3E0 | 5.0E-7 |
| UP_KEYWORDS | Mitochondrion inner membrane | RT | 23 | 2.9E-9 | 4.8E0 | 8.2E-7 |
| UP_KEYWORDS | Leber hereditary optic neuropathy | RT | 7 | 5.6E-9 | 4.0E1 | 8.2E-7 |
| UP_KEYWORDS | Mitochondrion | RT | 48 | 2.8E-8 | 2.4E0 | 1.6E-6 |
| UP_KEYWORDS | Electron transport | RT | 13 | 4.5E-7 | 6.8E0 | 1.9E-5 |
| UP_KEYWORDS | Respiratory chain | RT | 10 | 1.5E-6 | 9.0E0 | 5.0E-5 |
| UP_KEYWORDS | Ubiquinone | RT | 8 | 2.2E-6 | 1.3E1 | 6.5E-5 |
| KEGG_PATHWAY | Parkinson's disease | RT | 14 | 4.6E-6 | 4.9E0 | 4.5E-4 |
| GOTERM_BP_DIRECT | mitochondrial respiratory chain complex I assembly | RT | 9 | 2.7E-5 | 7.4E0 | 2.2E-2 |
| GOTERM_MF_DIRECT | NADH dehydrogenase (ubiquinone) activity | RT | 8 | 2.8E-5 | 8.9E0 | 1.4E-2 |
| GOTERM_CC_DIRECT | respiratory chain | RT | 6 | 3.1E-5 | 1.6E1 | 2.1E-3 |
| GOTERM_BP_DIRECT | mitochondrial electron transport, NADH to ubiquinone | RT | 8 | 3.9E-5 | 8.5E0 | 2.2E-2 |
| GOTERM_CC_DIRECT | mitochondrial respiratory chain complex I | RT | 7 | 3.3E-4 | 7.4E0 | 1.6E-2 |
| **Annotation Cluster 3** | **Enrichment Score: 5.86** | | **Count** | **P_Value** | **Fold Change** | **Benjamini** |
| UP_KEYWORDS | Endoplasmic reticulum | RT | 47 | 1.9E-8 | 2.5E0 | 1.2E-6 |
| GOTERM_CC_DIRECT | endoplasmic reticulum membrane | RT | 41 | 2.2E-7 | 2.5E0 | 1.9E-5 |
| GOTERM_CC_DIRECT | endoplasmic reticulum | RT | 31 | 6.4E-4 | 1.9E0 | 2.8E-2 |
| **Annotation Cluster 4** | **Enrichment Score: 4.4** | | **Count** | **P_Value** | **Fold Change** | **Benjamini** |
| GOTERM_BP_DIRECT | respiratory chain complex IV assembly | RT | 6 | 5.9E-7 | 3.1E1 | 9.9E-4 |
| GOTERM_MF_DIRECT | cytochrome-c oxidase activity | RT | 6 | 2.1E-4 | 1.1E1 | 5.3E-2 |
| GOTERM_BP_DIRECT | mitochondrial electron transport, cytochrome c to oxygen | RT | 5 | 5.1E-4 | 1.3E1 | 1.7E-1 |

| Category | Term | RT | Genes | Count | LT | PH | PT | % | P-Value | Fold Enrichment | Benjamini |
|---|---|---|---|---|---|---|---|---|---|---|---|
| UP_KEYWORDS | Membrane | RT | | 182 | 363 | 7494 | 20581 | 50.0 | 6.9E-8 | 1.4 | 3.3E-6 |
| UP_KEYWORDS | Transmembrane helix | RT | | 150 | 363 | 5634 | 20581 | 41.2 | 7.9E-9 | 1.5 | 8.2E-7 |
| UP_KEYWORDS | Transmembrane | RT | | 150 | 363 | 5651 | 20581 | 41.2 | 9.9E-9 | 1.5 | 8.2E-7 |
| GOTERM_CC_DIRECT | integral component of membrane | RT | | 147 | 350 | 5163 | 18224 | 40.4 | 3.4E-8 | 1.5 | 3.9E-6 |
| UP_SEQ_FEATURE | transmembrane region | RT | | 143 | 361 | 5056 | 20063 | 39.3 | 1.4E-9 | 1.6 | 1.9E-6 |
| UP_KEYWORDS | Disease mutation | RT | | 71 | 363 | 2550 | 20581 | 19.5 | 9.3E-5 | 1.6 | 2.4E-3 |
| UP_KEYWORDS | Transport | RT | | 65 | 363 | 1978 | 20581 | 17.9 | 1.2E-6 | 1.9 | 4.4E-5 |
| GOTERM_CC_DIRECT | mitochondrion | RT | | 59 | 350 | 1331 | 18224 | 16.2 | 2.8E-9 | 2.3 | 5.0E-7 |
| UP_KEYWORDS | Mitochondrion | RT | | 48 | 363 | 1119 | 20581 | 13.2 | 2.8E-8 | 2.4 | 1.6E-6 |
| UP_KEYWORDS | Endoplasmic reticulum | RT | | 47 | 363 | 1067 | 20581 | 12.9 | 1.9E-8 | 2.5 | 1.2E-6 |
| GOTERM_CC_DIRECT | endoplasmic reticulum membrane | RT | | 41 | 350 | 862 | 18224 | 11.3 | 2.2E-7 | 2.5 | 1.9E-5 |
| GOTERM_CC_DIRECT | mitochondrial inner membrane | RT | | 31 | 350 | 441 | 18224 | 8.5 | 2.1E-9 | 3.7 | 5.0E-7 |
| GOTERM_CC_DIRECT | endoplasmic reticulum | RT | | 31 | 350 | 828 | 18224 | 8.5 | 6.4E-4 | 1.9 | 2.8E-2 |
| UP_KEYWORDS | Mitochondrion inner membrane | RT | | 23 | 363 | 270 | 20581 | 6.3 | 2.9E-9 | 4.8 | 8.2E-7 |
| KEGG_PATHWAY | Oxidative phosphorylation | RT | | 18 | 139 | 133 | 6879 | 4.9 | 8.8E-10 | 6.7 | 1.7E-7 |
| KEGG_PATHWAY | Parkinson's disease | RT | | 14 | 139 | 142 | 6879 | 3.8 | 4.6E-6 | 4.9 | 4.5E-4 |
| KEGG_PATHWAY | Non-alcoholic fatty liver disease (NAFLD) | RT | | 14 | 139 | 151 | 6879 | 3.8 | 9.1E-6 | 4.6 | 5.9E-4 |
| UP_KEYWORDS | Electron transport | RT | | 13 | 363 | 108 | 20581 | 3.6 | 4.5E-7 | 6.8 | 1.9E-5 |
| UP_KEYWORDS | Lipid biosynthesis | RT | | 11 | 363 | 156 | 20581 | 3.0 | 4.5E-4 | 4.0 | 1.0E-2 |
| UP_KEYWORDS | Respiratory chain | RT | | 10 | 363 | 63 | 20581 | 2.7 | 1.5E-6 | 9.0 | 5.0E-5 |
| GOTERM_CC_DIRECT | mitochondrial membrane | RT | | 10 | 350 | 94 | 18224 | 2.7 | 7.9E-5 | 5.5 | 4.6E-3 |

‘Glass bead’ proteins (n=193)

| Annotation Cluster 1 | Enrichment Score: 8.19 | | Count | P_Value | Fold Change | Benjamini |
|---|---|---|---|---|---|---|
| UP_SEQ_FEATURE | transmembrane region | RT | 89 | 1.5E-10 | 1.9E0 | 8.4E-8 |
| UP_KEYWORDS | Transmembrane helix | RT | 92 | 1.2E-9 | 1.8E0 | 1.8E-7 |
| UP_KEYWORDS | Transmembrane | RT | 92 | 1.4E-9 | 1.8E0 | 1.8E-7 |
| GOTERM_CC_DIRECT | integral component of membrane | RT | 89 | 1.4E-8 | 1.7E0 | 1.7E-6 |
| UP_KEYWORDS | Membrane | RT | 101 | 3.4E-6 | 1.5E0 | 9.7E-5 |
| **Annotation Cluster 2** | **Enrichment Score: 6.15** | | **Count** | **P_Value** | **Fold Change** | **Benjamini** |
| KEGG_PATHWAY | Oxidative phosphorylation | RT | 14 | 5.4E-10 | 1.0E1 | 8.0E-8 |
| GOTERM_CC_DIRECT | mitochondrial inner membrane | RT | 22 | 4.1E-9 | 4.9E0 | 9.6E-7 |
| UP_KEYWORDS | Leber hereditary optic neuropathy | RT | 6 | 1.5E-8 | 6.5E1 | 1.0E-6 |
| UP_KEYWORDS | Mitochondrion inner membrane | RT | 16 | 3.4E-8 | 6.4E0 | 1.7E-6 |
| UP_KEYWORDS | Mitochondrion | RT | 32 | 3.9E-8 | 3.1E0 | 1.7E-6 |
| GOTERM_CC_DIRECT | mitochondrion | RT | 37 | 4.5E-8 | 2.7E0 | 3.5E-6 |
| KEGG_PATHWAY | Parkinson's disease | RT | 12 | 1.5E-7 | 8.2E0 | 1.1E-5 |
| UP_KEYWORDS | Electron transport | RT | 10 | 7.2E-7 | 1.0E1 | 2.7E-5 |
| GOTERM_CC_DIRECT | respiratory chain | RT | 6 | 1.4E-6 | 3.0E1 | 7.9E-5 |
| UP_KEYWORDS | Respiratory chain | RT | 8 | 1.8E-6 | 1.4E1 | 5.9E-5 |
| UP_KEYWORDS | Ubiquinone | RT | 6 | 1.6E-5 | 1.8E1 | 4.3E-4 |
| GOTERM_BP_DIRECT | mitochondrial respiratory chain complex I assembly | RT | 7 | 4.3E-5 | 1.1E1 | 4.2E-2 |
| GOTERM_MF_DIRECT | NADH dehydrogenase (ubiquinone) activity | RT | 6 | 1.1E-4 | 1.3E1 | 3.3E-2 |
| GOTERM_BP_DIRECT | mitochondrial electron transport, NADH to ubiquinone | RT | 6 | 1.4E-4 | 1.2E1 | 6.7E-2 |
| UP_KEYWORDS | MELAS syndrome | RT | 3 | 8.3E-4 | 6.5E1 | 1.5E-2 |

| Category | Term | RT | Genes | Count | LT | PH | PT | % | P-Value | Fold Enrichment | Benjamini |
|---|---|---|---|---|---|---|---|---|---|---|---|
| UP_KEYWORDS | Membrane | RT | | 101 | 191 | 7494 | 20581 | 52.9 | 3.4E-6 | 1.5 | 9.7E-5 |
| UP_KEYWORDS | Transmembrane helix | RT | | 92 | 191 | 5634 | 20581 | 48.2 | 1.2E-9 | 1.8 | 1.8E-7 |
| UP_KEYWORDS | Transmembrane | RT | | 92 | 191 | 5651 | 20581 | 48.2 | 1.4E-9 | 1.8 | 1.8E-7 |
| UP_SEQ_FEATURE | transmembrane region | RT | | 89 | 190 | 5056 | 20063 | 46.6 | 1.5E-10 | 1.9 | 8.4E-8 |
| GOTERM_CC_DIRECT | integral component of membrane | RT | | 89 | 185 | 5163 | 18224 | 46.6 | 1.4E-8 | 1.7 | 1.7E-6 |
| GOTERM_CC_DIRECT | mitochondrion | RT | | 37 | 185 | 1331 | 18224 | 19.4 | 4.5E-8 | 2.7 | 3.5E-6 |
| UP_KEYWORDS | Transport | RT | | 36 | 191 | 1978 | 20581 | 18.8 | 1.3E-4 | 2.0 | 3.0E-3 |
| UP_KEYWORDS | Endoplasmic reticulum | RT | | 33 | 191 | 1067 | 20581 | 17.3 | 3.5E-9 | 3.3 | 3.0E-7 |
| UP_KEYWORDS | Mitochondrion | RT | | 32 | 191 | 1119 | 20581 | 16.8 | 3.9E-8 | 3.1 | 1.7E-6 |
| GOTERM_CC_DIRECT | endoplasmic reticulum membrane | RT | | 25 | 185 | 862 | 18224 | 13.1 | 6.5E-6 | 2.9 | 3.0E-4 |
| GOTERM_CC_DIRECT | mitochondrial inner membrane | RT | | 22 | 185 | 441 | 18224 | 11.5 | 4.1E-9 | 4.9 | 9.6E-7 |
| UP_KEYWORDS | Mitochondrion inner membrane | RT | | 16 | 191 | 270 | 20581 | 8.4 | 3.4E-8 | 6.4 | 1.7E-6 |
| KEGG_PATHWAY | Oxidative phosphorylation | RT | | 14 | 71 | 133 | 6879 | 7.3 | 5.4E-10 | 10.2 | 8.0E-8 |
| KEGG_PATHWAY | Parkinson's disease | RT | | 12 | 71 | 142 | 6879 | 6.3 | 1.5E-7 | 8.2 | 1.1E-5 |
| UP_KEYWORDS | Electron transport | RT | | 10 | 191 | 108 | 20581 | 5.2 | 7.2E-7 | 10.0 | 2.7E-5 |

**All SP4 proteins combined (n=400)**

| Annotation Cluster 1 | Enrichment Score: 7.14 | | Count | P_Value | Fold Change | Benjamini |
|---|---|---|---|---|---|---|
| UP_SEQ_FEATURE | transmembrane region | RT | 151 | 8.5E-9 | 1.5E0 | 1.2E-5 |
| UP_KEYWORDS | Transmembrane helix | RT | 159 | 4.3E-8 | 1.5E0 | 4.7E-6 |
| UP_KEYWORDS | Transmembrane | RT | 159 | 5.3E-8 | 1.5E0 | 4.7E-6 |
| GOTERM_CC_DIRECT | integral component of membrane | RT | 156 | 1.1E-7 | 1.4E0 | 1.4E-5 |
| UP_KEYWORDS | Membrane | RT | 192 | 9.6E-7 | 1.3E0 | 4.2E-5 |
| **Annotation Cluster 2** | **Enrichment Score: 6.06** | | **Count** | **P_Value** | **Fold Change** | **Benjamini** |
| KEGG_PATHWAY | Oxidative phosphorylation | RT | 19 | 5.2E-10 | 6.4E0 | 1.0E-7 |
| UP_KEYWORDS | Mitochondrion inner membrane | RT | 24 | 3.1E-9 | 4.6E0 | 1.1E-6 |
| GOTERM_CC_DIRECT | mitochondrial inner membrane | RT | 32 | 4.1E-9 | 3.5E0 | 1.5E-6 |
| UP_KEYWORDS | Leber hereditary optic neuropathy | RT | 7 | 9.8E-9 | 3.6E1 | 1.7E-6 |
| GOTERM_CC_DIRECT | mitochondrion | RT | 61 | 1.2E-8 | 2.2E0 | 2.2E-6 |
| UP_KEYWORDS | Mitochondrion | RT | 49 | 1.9E-7 | 2.3E0 | 1.1E-5 |
| UP_KEYWORDS | Electron transport | RT | 13 | 1.2E-6 | 6.2E0 | 4.6E-5 |
| KEGG_PATHWAY | Parkinson's disease | RT | 15 | 2.5E-6 | 4.7E0 | 2.6E-4 |
| UP_KEYWORDS | Respiratory chain | RT | 10 | 3.2E-6 | 8.2E0 | 1.1E-4 |
| UP_KEYWORDS | Ubiquinone | RT | 8 | 4.0E-6 | 1.2E1 | 1.3E-4 |
| KEGG_PATHWAY | Non-alcoholic fatty liver disease (NAFLD) | RT | 15 | 5.3E-6 | 4.5E0 | 3.5E-4 |
| GOTERM_CC_DIRECT | respiratory chain | RT | 6 | 4.7E-5 | 1.4E1 | 3.4E-3 |
| GOTERM_BP_DIRECT | mitochondrial respiratory chain complex I assembly | RT | 9 | 5.0E-5 | 6.8E0 | 4.0E-2 |
| GOTERM_MF_DIRECT | NADH dehydrogenase (ubiquinone) activity | RT | 8 | 5.4E-5 | 8.0E0 | 1.4E-2 |
| GOTERM_BP_DIRECT | mitochondrial electron transport, NADH to ubiquinone | RT | 8 | 6.9E-5 | 7.7E0 | 4.0E-2 |
| GOTERM_CC_DIRECT | mitochondrial respiratory chain complex I | RT | 7 | 5.3E-4 | 6.8E0 | 2.7E-2 |
| **Annotation Cluster 3** | **Enrichment Score: 4.57** | | **Count** | **P_Value** | **Fold Change** | **Benjamini** |
| GOTERM_BP_DIRECT | respiratory chain complex IV assembly | RT | 6 | 9.2E-7 | 2.8E1 | 1.6E-3 |
| GOTERM_MF_DIRECT | cytochrome-c oxidase activity | RT | 7 | 2.9E-5 | 1.1E1 | 1.4E-2 |
| GOTERM_BP_DIRECT | mitochondrial electron transport, cytochrome c to oxygen | RT | 5 | 7.1E-4 | 1.2E1 | 2.5E-1 |

| Category | Term | RT | Genes | Count | LT | PH | PT | % | P-Value | Fold Enrichment | Benjamini |
|---|---|---|---|---|---|---|---|---|---|---|---|
| UP_KEYWORDS | Membrane | RT | | 192 | 398 | 7494 | 20581 | 48.1 | 9.6E-7 | 1.3 | 4.2E-5 |
| UP_KEYWORDS | Transmembrane helix | RT | | 159 | 398 | 5634 | 20581 | 39.8 | 4.3E-8 | 1.5 | 4.7E-6 |
| UP_KEYWORDS | Transmembrane | RT | | 159 | 398 | 5651 | 20581 | 39.8 | 5.3E-8 | 1.5 | 4.7E-6 |
| GOTERM_CC_DIRECT | integral component of membrane | RT | | 156 | 382 | 5163 | 18224 | 39.1 | 1.1E-7 | 1.4 | 1.4E-5 |
| UP_SEQ_FEATURE | transmembrane region | RT | | 151 | 395 | 5056 | 20063 | 37.8 | 8.5E-9 | 1.5 | 1.2E-5 |
| UP_KEYWORDS | Disease mutation | RT | | 77 | 398 | 2550 | 20581 | 19.3 | 6.8E-5 | 1.6 | 1.9E-3 |
| UP_KEYWORDS | Transport | RT | | 70 | 398 | 1978 | 20581 | 17.5 | 8.8E-7 | 1.8 | 4.2E-5 |
| GOTERM_CC_DIRECT | mitochondrion | RT | | 61 | 382 | 1331 | 18224 | 15.3 | 1.2E-8 | 2.2 | 2.2E-6 |
| UP_KEYWORDS | Mitochondrion | RT | | 49 | 398 | 1119 | 20581 | 12.3 | 1.9E-7 | 2.3 | 1.1E-5 |
| UP_KEYWORDS | Endoplasmic reticulum | RT | | 48 | 398 | 1067 | 20581 | 12.0 | 1.2E-7 | 2.3 | 8.2E-6 |
| GOTERM_CC_DIRECT | endoplasmic reticulum membrane | RT | | 41 | 382 | 862 | 18224 | 10.3 | 2.1E-6 | 2.3 | 1.9E-4 |
| GOTERM_CC_DIRECT | mitochondrial inner membrane | RT | | 32 | 382 | 441 | 18224 | 8.0 | 4.1E-9 | 3.5 | 1.5E-6 |
| UP_KEYWORDS | Mitochondrion inner membrane | RT | | 24 | 398 | 270 | 20581 | 6.0 | 3.1E-9 | 4.6 | 1.1E-6 |
| KEGG_PATHWAY | Oxidative phosphorylation | RT | | 19 | 153 | 133 | 6879 | 4.8 | 5.2E-10 | 6.4 | 1.0E-7 |
| KEGG_PATHWAY | Parkinson's disease | RT | | 15 | 153 | 142 | 6879 | 3.8 | 2.5E-6 | 4.7 | 2.6E-4 |
| KEGG_PATHWAY | Non-alcoholic fatty liver disease (NAFLD) | RT | | 15 | 153 | 151 | 6879 | 3.8 | 5.3E-6 | 4.5 | 3.5E-4 |
| UP_KEYWORDS | Electron transport | RT | | 13 | 398 | 108 | 20581 | 3.3 | 1.2E-6 | 6.2 | 4.6E-5 |
| UP_KEYWORDS | Lipid biosynthesis | RT | | 11 | 398 | 156 | 20581 | 2.8 | 9.2E-4 | 3.6 | 2.0E-2 |
| UP_KEYWORDS | Respiratory chain | RT | | 10 | 398 | 63 | 20581 | 2.5 | 3.2E-6 | 8.2 | 1.1E-4 |
| GOTERM_CC_DIRECT | mitochondrial membrane | RT | | 10 | 382 | 94 | 18224 | 2.5 | 1.5E-4 | 5.1 | 9.3E-3 |

‘SP3’ proteins combined vs. BF+GB (n=73)

No significant clustered terms

| Category | Term | RT | Genes | Count | LT | PH | PT | % | P-Value | Fold Enrichment | Benjamini |
|---|---|---|---|---|---|---|---|---|---|---|---|
| UP_KEYWORDS | Acetylation | RT | | 25 | 72 | 3424 | 20581 | 34.7 | 3.2E-4 | 2.1 | 2.1E-2 |
| UP_KEYWORDS | Palmoplantar keratoderma | RT | | 4 | 72 | 32 | 20581 | 5.6 | 1.8E-4 | 35.7 | 2.1E-2 |

# Figure S8 – TMT ii.

**Functional annotation clustering:** / **Functional annotation terms:**

## SP4-GB / SP3 80% ACN proteins (n=367):

Functional annotation clustering:

| Category | Term | RT | Count | P_Value | Fold Change | Benjamini |
|---|---|---|---|---|---|---|
| **Annotation Cluster 1** | **Enrichment Score: 9.96** | | **Count** | **P_Value** | **Fold Change** | **Benjamini** |
| GOTERM_CC_DIRECT | endoplasmic reticulum membrane | RT | 56 | 1.0E-14 | 3.3E0 | 3.7E-12 |
| UP_KEYWORDS | Endoplasmic reticulum | RT | 57 | 2.7E-13 | 3.0E0 | 4.1E-11 |
| GOTERM_CC_DIRECT | endoplasmic reticulum | RT | 32 | 4.8E-4 | 2.0E0 | 1.6E-2 |
| **Annotation Cluster 2** | **Enrichment Score: 9.21** | | **Count** | **P_Value** | **Fold Change** | **Benjamini** |
| UP_KEYWORDS | Transmembrane | RT | 159 | 4.2E-11 | 1.6E0 | 3.2E-9 |
| UP_KEYWORDS | Transmembrane helix | RT | 158 | 6.8E-11 | 1.6E0 | 4.1E-9 |
| UP_SEQ_FEATURE | transmembrane region | RT | 143 | 1.2E-9 | 1.6E0 | 9.7E-7 |
| UP_KEYWORDS | Membrane | RT | 189 | 2.1E-9 | 1.4E0 | 8.9E-8 |
| GOTERM_CC_DIRECT | integral component of membrane | RT | 152 | 1.3E-8 | 1.5E0 | 1.3E-6 |
| **Annotation Cluster 3** | **Enrichment Score: 8.48** | | **Count** | **P_Value** | **Fold Change** | **Benjamini** |
| GOTERM_CC_DIRECT | mitochondrial inner membrane | RT | 33 | 2.3E-10 | 3.8E0 | 4.3E-8 |
| UP_KEYWORDS | Mitochondrion inner membrane | RT | 24 | 6.1E-10 | 5.0E0 | 3.0E-8 |
| UP_KEYWORDS | Mitochondrion | RT | 46 | 2.5E-7 | 2.3E0 | 6.9E-6 |
| **Annotation Cluster 4** | **Enrichment Score: 5.86** | | **Count** | **P_Value** | **Fold Change** | **Benjamini** |
| KEGG_PATHWAY | N-Glycan biosynthesis | RT | 12 | 6.9E-9 | 1.1E1 | 1.2E-6 |
| UP_KEYWORDS | Congenital disorder of glycosylation | RT | 10 | 4.2E-8 | 1.3E1 | 1.6E-6 |
| GOTERM_BP_DIRECT | dolichol-linked oligosaccharide biosynthetic process | RT | 6 | 8.0E-6 | 2.0E1 | 2.7E-3 |

Functional annotation terms:

| Category | Term | RT | Genes | Count | LT | PH | PT | % | P-Value | Fold Enrichment | Benjamini |
|---|---|---|---|---|---|---|---|---|---|---|---|
| GOTERM_CC_DIRECT | endoplasmic reticulum membrane | RT | | 56 | 360 | 862 | 18224 | 15.3 | 1.0E-14 | 3.3 | 3.7E-12 |
| UP_KEYWORDS | Transport | RT | | 83 | 366 | 1978 | 20581 | 22.6 | 1.7E-13 | 2.4 | 4.1E-11 |
| UP_KEYWORDS | Endoplasmic reticulum | RT | | 57 | 366 | 1067 | 20581 | 15.5 | 2.7E-13 | 3.0 | 4.1E-11 |
| UP_KEYWORDS | Acetylation | RT | | 116 | 366 | 3424 | 20581 | 31.6 | 1.3E-12 | 1.9 | 1.3E-10 |
| UP_KEYWORDS | Transmembrane | RT | | 159 | 366 | 5651 | 20581 | 43.3 | 4.2E-11 | 1.6 | 3.2E-9 |
| UP_KEYWORDS | Transmembrane helix | RT | | 158 | 366 | 5634 | 20581 | 43.1 | 6.8E-11 | 1.6 | 4.1E-9 |
| GOTERM_CC_DIRECT | mitochondrial inner membrane | RT | | 33 | 360 | 441 | 18224 | 9.0 | 2.3E-10 | 3.8 | 4.3E-8 |
| UP_KEYWORDS | Mitochondrion inner membrane | RT | | 24 | 366 | 270 | 20581 | 6.5 | 6.1E-10 | 5.0 | 3.0E-8 |
| UP_SEQ_FEATURE | transmembrane region | RT | | 143 | 360 | 5056 | 20063 | 39.0 | 1.2E-9 | 1.6 | 9.7E-7 |
| UP_KEYWORDS | Membrane | RT | | 189 | 366 | 7494 | 20581 | 51.5 | 2.1E-9 | 1.4 | 8.9E-8 |
| KEGG_PATHWAY | N-Glycan biosynthesis | RT | | 12 | 155 | 49 | 6879 | 3.3 | 6.9E-9 | 10.9 | 1.2E-6 |
| GOTERM_CC_DIRECT | integral component of membrane | RT | | 152 | 360 | 5163 | 18224 | 41.4 | 1.3E-8 | 1.5 | 1.3E-6 |
| GOTERM_CC_DIRECT | membrane | RT | | 82 | 360 | 2200 | 18224 | 22.3 | 1.4E-8 | 1.9 | 1.3E-6 |
| UP_KEYWORDS | Congenital disorder of glycosylation | RT | | 10 | 366 | 42 | 20581 | 2.7 | 4.2E-8 | 13.4 | 1.6E-6 |
| GOTERM_BP_DIRECT | rRNA processing | RT | | 20 | 338 | 214 | 16792 | 5.4 | 6.7E-8 | 4.6 | 8.9E-5 |
| UP_KEYWORDS | ER-Golgi transport | RT | | 13 | 366 | 94 | 20581 | 3.5 | 1.0E-7 | 7.8 | 3.5E-6 |
| INTERPRO | Small GTPase superfamily, ARF type | RT | | 9 | 354 | 32 | 18559 | 2.5 | 1.1E-7 | 14.7 | 5.7E-5 |
| UP_KEYWORDS | Protein transport | RT | | 32 | 366 | 610 | 20581 | 8.7 | 1.7E-7 | 2.9 | 5.0E-6 |
| INTERPRO | Small GTPase superfamily, ARF/SAR type | RT | | 9 | 354 | 34 | 18559 | 2.5 | 1.9E-7 | 13.9 | 5.7E-5 |
| UP_KEYWORDS | Mitochondrion | RT | | 46 | 366 | 1119 | 20581 | 12.5 | 2.5E-7 | 2.3 | 6.9E-6 |

## SP4-GB / SP3 50% ACN proteins (n=411):

Functional annotation clustering:

| Category | Term | RT | Count | P_Value | Fold Change | Benjamini |
|---|---|---|---|---|---|---|
| **Annotation Cluster 1** | **Enrichment Score: 11.74** | | **Count** | **P_Value** | **Fold Change** | **Benjamini** |
| UP_KEYWORDS | Mitochondrion inner membrane | RT | 30 | 1.5E-13 | 5.6E0 | 1.6E-11 |
| GOTERM_CC_DIRECT | mitochondrial inner membrane | RT | 38 | 2.1E-12 | 4.0E0 | 4.1E-10 |
| UP_KEYWORDS | Mitochondrion | RT | 59 | 1.8E-11 | 2.7E0 | 1.1E-9 |
| **Annotation Cluster 2** | **Enrichment Score: 8.06** | | **Count** | **P_Value** | **Fold Change** | **Benjamini** |
| UP_KEYWORDS | Transmembrane | RT | 170 | 5.8E-10 | 1.5E0 | 3.0E-8 |
| UP_KEYWORDS | Transmembrane helix | RT | 169 | 8.7E-10 | 1.5E0 | 3.8E-8 |
| UP_KEYWORDS | Membrane | RT | 205 | 1.0E-8 | 1.4E0 | 4.0E-7 |
| UP_SEQ_FEATURE | transmembrane region | RT | 151 | 4.7E-8 | 1.5E0 | 4.4E-5 |
| GOTERM_CC_DIRECT | integral component of membrane | RT | 160 | 2.1E-7 | 1.4E0 | 1.6E-5 |
| **Annotation Cluster 3** | **Enrichment Score: 3.49** | | **Count** | **P_Value** | **Fold Change** | **Benjamini** |
| KEGG_PATHWAY | N-Glycan biosynthesis | RT | 11 | 1.2E-7 | 9.7E0 | 7.7E-6 |
| GOTERM_CC_DIRECT | oligosaccharyltransferase complex | RT | 6 | 1.1E-6 | 2.8E1 | 5.9E-5 |
| UP_KEYWORDS | Congenital disorder of glycosylation | RT | 9 | 1.5E-6 | 1.1E1 | 4.1E-5 |
| GOTERM_MF_DIRECT | dolichyl-diphosphooligosaccharide-protein glycotransferase activity | RT | 5 | 2.3E-5 | 2.6E1 | 1.2E-2 |
| GOTERM_BP_DIRECT | protein N-linked glycosylation via asparagine | RT | 8 | 2.6E-5 | 8.9E0 | 3.5E-2 |

Functional annotation terms:

| Category | Term | RT | Genes | Count | LT | PH | PT | % | P-Value | Fold Enrichment | Benjamini |
|---|---|---|---|---|---|---|---|---|---|---|---|
| UP_KEYWORDS | Transport | RT | | 99 | 409 | 1978 | 20581 | 24.1 | 4.3E-18 | 2.5 | 9.6E-16 |
| UP_KEYWORDS | Acetylation | RT | | 139 | 409 | 3424 | 20581 | 33.8 | 6.2E-18 | 2.0 | 9.6E-16 |
| GOTERM_CC_DIRECT | endoplasmic reticulum membrane | RT | | 60 | 397 | 862 | 18224 | 14.6 | 3.8E-15 | 3.2 | 1.4E-12 |
| UP_KEYWORDS | Mitochondrion inner membrane | RT | | 30 | 409 | 270 | 20581 | 7.3 | 1.5E-13 | 5.6 | 1.6E-11 |
| UP_KEYWORDS | Endoplasmic reticulum | RT | | 60 | 409 | 1067 | 20581 | 14.6 | 8.4E-13 | 2.8 | 6.5E-11 |
| GOTERM_CC_DIRECT | mitochondrial inner membrane | RT | | 38 | 397 | 441 | 18224 | 9.2 | 2.1E-12 | 4.0 | 4.1E-10 |
| UP_KEYWORDS | Mitochondrion | RT | | 59 | 409 | 1119 | 20581 | 14.4 | 1.8E-11 | 2.7 | 1.1E-9 |
| UP_KEYWORDS | Transmembrane | RT | | 170 | 409 | 5651 | 20581 | 41.4 | 5.8E-10 | 1.5 | 3.0E-8 |
| UP_KEYWORDS | Transmembrane helix | RT | | 169 | 409 | 5634 | 20581 | 41.1 | 8.7E-10 | 1.5 | 3.8E-8 |
| GOTERM_CC_DIRECT | mitochondrion | RT | | 65 | 397 | 1331 | 18224 | 15.8 | 1.4E-9 | 2.2 | 1.7E-7 |
| KEGG_PATHWAY | Metabolic pathways | RT | | 59 | 160 | 1219 | 6879 | 14.4 | 9.5E-9 | 2.1 | 1.8E-6 |
| UP_KEYWORDS | Membrane | RT | | 205 | 409 | 7494 | 20581 | 49.9 | 1.0E-8 | 1.4 | 4.0E-7 |
| UP_KEYWORDS | Protein transport | RT | | 36 | 409 | 610 | 20581 | 8.8 | 2.1E-8 | 3.0 | 7.1E-7 |
| GOTERM_CC_DIRECT | membrane | RT | | 87 | 397 | 2200 | 18224 | 21.2 | 3.3E-8 | 1.8 | 3.1E-6 |
| UP_SEQ_FEATURE | transmembrane region | RT | | 151 | 404 | 5056 | 20063 | 36.7 | 4.7E-8 | 1.5 | 4.4E-5 |
| KEGG_PATHWAY | Oxidative phosphorylation | RT | | 17 | 160 | 133 | 6879 | 4.1 | 5.4E-8 | 5.5 | 5.0E-6 |
| KEGG_PATHWAY | N-Glycan biosynthesis | RT | | 11 | 160 | 49 | 6879 | 2.7 | 1.2E-7 | 9.7 | 7.7E-6 |
| GOTERM_CC_DIRECT | integral component of membrane | RT | | 160 | 397 | 5163 | 18224 | 38.9 | 2.1E-7 | 1.4 | 1.6E-5 |
| UP_KEYWORDS | ER-Golgi transport | RT | | 13 | 409 | 94 | 20581 | 3.2 | 3.5E-7 | 7.0 | 1.1E-5 |
| GOTERM_CC_DIRECT | extracellular exosome | RT | | 99 | 397 | 2811 | 18224 | 24.1 | 7.4E-7 | 1.6 | 4.7E-5 |

## SP3 (80+50 % ACN) / SP4-GB proteins (n=185, combined):

Functional annotation clustering:

| Category | Term | RT | Count | P_Value | Fold Change | Benjamini |
|---|---|---|---|---|---|---|
| **Annotation Cluster 1** | **Enrichment Score: 2.78** | | **Count** | **P_Value** | **Fold Change** | **Benjamini** |
| UP_SEQ_FEATURE | short sequence motif:Twin CX3C motif | RT | 4 | 1.4E-5 | 7.3E1 | 7.5E-3 |
| INTERPRO | Tim10/DDP family zinc finger | RT | 4 | 1.5E-5 | 7.2E1 | 5.2E-3 |
| GOTERM_CC_DIRECT | mitochondrial intermembrane space | RT | 7 | 5.4E-5 | 1.0E1 | 6.3E-3 |
| GOTERM_CC_DIRECT | mitochondrial intermembrane space protein transporter complex | RT | 3 | 7.9E-4 | 6.6E1 | 2.6E-2 |
| **Annotation Cluster 2** | **Enrichment Score: 2.22** | | **Count** | **P_Value** | **Fold Change** | **Benjamini** |
| GOTERM_CC_DIRECT | mitochondrial inner membrane | RT | 16 | 1.1E-5 | 4.0E0 | 2.6E-3 |
| KEGG_PATHWAY | Oxidative phosphorylation | RT | 8 | 2.1E-4 | 6.4E0 | 3.3E-2 |
| UP_KEYWORDS | Mitochondrion inner membrane | RT | 10 | 6.9E-4 | 4.2E0 | 2.6E-2 |

Functional annotation terms:

| Category | Term | RT | Genes | Count | LT | PH | PT | % | P-Value | Fold Enrichment | Benjamini |
|---|---|---|---|---|---|---|---|---|---|---|---|
| UP_KEYWORDS | Acetylation | RT | | 69 | 183 | 3424 | 20581 | 37.3 | 1.3E-11 | 2.3 | 2.9E-9 |
| GOTERM_MF_DIRECT | protein binding | RT | | 114 | 159 | 8785 | 16881 | 61.6 | 4.1E-7 | 1.4 | 1.2E-4 |
| UP_KEYWORDS | Mitochondrion | RT | | 29 | 183 | 1119 | 20581 | 15.7 | 6.1E-7 | 2.9 | 6.8E-5 |
| GOTERM_CC_DIRECT | mitochondrial inner membrane | RT | | 16 | 165 | 441 | 18224 | 8.6 | 1.1E-5 | 4.0 | 2.6E-3 |
| UP_SEQ_FEATURE | short sequence motif:Twin CX3C motif | RT | | 4 | 182 | 6 | 20063 | 2.2 | 1.4E-5 | 73.5 | 7.5E-3 |
| INTERPRO | Tim10/DDP family zinc finger | RT | | 4 | 171 | 6 | 18559 | 2.2 | 1.5E-5 | 72.4 | 5.2E-3 |
| GOTERM_CC_DIRECT | mitochondrial intermembrane space | RT | | 7 | 165 | 74 | 18224 | 3.8 | 5.4E-5 | 10.4 | 6.3E-3 |
| UP_KEYWORDS | Cytoplasm | RT | | 66 | 183 | 4816 | 20581 | 35.7 | 1.1E-4 | 1.5 | 8.5E-3 |
| GOTERM_CC_DIRECT | mitochondrion | RT | | 27 | 165 | 1331 | 18224 | 14.6 | 1.4E-4 | 2.2 | 8.9E-3 |
| GOTERM_CC_DIRECT | cytoplasm | RT | | 70 | 165 | 5222 | 18224 | 37.8 | 1.5E-4 | 1.5 | 8.9E-3 |
| KEGG_PATHWAY | Oxidative phosphorylation | RT | | 8 | 65 | 133 | 6879 | 4.3 | 2.1E-4 | 6.4 | 3.3E-2 |
| GOTERM_CC_DIRECT | mediator complex | RT | | 5 | 165 | 35 | 18224 | 2.7 | 2.7E-4 | 15.8 | 1.2E-2 |
| UP_KEYWORDS | Activator | RT | | 17 | 183 | 661 | 20581 | 9.2 | 2.7E-4 | 2.9 | 1.5E-2 |
| UP_KEYWORDS | Nucleus | RT | | 68 | 183 | 5244 | 20581 | 36.8 | 4.5E-4 | 1.5 | 2.0E-2 |
| UP_KEYWORDS | Mitochondrion inner membrane | RT | | 10 | 183 | 270 | 20581 | 5.4 | 6.9E-4 | 4.2 | 2.6E-2 |
| GOTERM_CC_DIRECT | nucleoplasm | RT | | 42 | 165 | 2784 | 18224 | 22.7 | 7.7E-4 | 1.7 | 2.6E-2 |
| GOTERM_CC_DIRECT | mitochondrial intermembrane space protein transporter complex | RT | | 3 | 165 | 5 | 18224 | 1.6 | 7.9E-4 | 66.3 | 2.6E-2 |

## SP3 80% ACN / SP3 50% ACN proteins (n=127)

Functional annotation clustering:

| Category | Term | RT | Count | P_Value | Fold Change | Benjamini |
|---|---|---|---|---|---|---|
| **Annotation Cluster 1** | **Enrichment Score: 6.37** | | **Count** | **P_Value** | **Fold Change** | **Benjamini** |
| UP_KEYWORDS | Mitochondrion | RT | 31 | 3.7E-12 | 4.5E0 | 3.2E-10 |
| UP_KEYWORDS | Mitochondrion inner membrane | RT | 16 | 9.9E-11 | 9.7E0 | 5.7E-9 |
| GOTERM_CC_DIRECT | mitochondrion | RT | 31 | 8.3E-10 | 3.6E0 | 8.5E-8 |
| GOTERM_CC_DIRECT | mitochondrial inner membrane | RT | 18 | 3.2E-9 | 6.3E0 | 1.7E-7 |
| KEGG_PATHWAY | Oxidative phosphorylation | RT | 11 | 3.5E-9 | 1.4E1 | 2.6E-7 |
| KEGG_PATHWAY | Huntington's disease | RT | 10 | 1.4E-6 | 8.5E0 | 5.2E-5 |
| KEGG_PATHWAY | Parkinson's disease | RT | 8 | 1.7E-5 | 9.2E0 | 4.3E-4 |
| UP_KEYWORDS | Transit peptide | RT | 14 | 2.3E-5 | 4.3E0 | 6.8E-4 |
| KEGG_PATHWAY | Alzheimer's disease | RT | 8 | 5.1E-5 | 7.8E0 | 9.6E-4 |
| UP_SEQ_FEATURE | transit peptide:Mitochondrion | RT | 12 | 1.9E-4 | 4.0E0 | 2.7E-2 |
| KEGG_PATHWAY | Metabolic pathways | RT | 17 | 1.0E-3 | 2.3E0 | 1.5E-2 |
| KEGG_PATHWAY | Non-alcoholic fatty liver disease (NAFLD) | RT | 6 | 1.9E-3 | 6.5E0 | 2.4E-2 |
| **Annotation Cluster 2** | **Enrichment Score: 4.13** | | **Count** | **P_Value** | **Fold Change** | **Benjamini** |
| INTERPRO | Tim10/DDP family zinc finger | RT | 6 | 4.4E-11 | 1.6E2 | 8.5E-9 |
| UP_SEQ_FEATURE | short sequence motif:Twin CX3C motif | RT | 6 | 4.8E-11 | 1.6E2 | 1.3E-8 |
| GOTERM_CC_DIRECT | mitochondrial intermembrane space protein transporter complex | RT | 5 | 8.0E-9 | 1.5E2 | 3.2E-7 |
| GOTERM_BP_DIRECT | chaperone-mediated protein transport | RT | 5 | 1.2E-7 | 9.5E1 | 6.0E-5 |
| UP_KEYWORDS | Chaperone | RT | 11 | 4.2E-7 | 8.9E0 | 1.8E-5 |
| GOTERM_BP_DIRECT | protein targeting to mitochondrion | RT | 6 | 2.5E-6 | 2.7E1 | 6.5E-4 |
| UP_KEYWORDS | Translocation | RT | 7 | 1.2E-5 | 1.4E1 | 4.2E-4 |
| GOTERM_BP_DIRECT | protein import into mitochondrial inner membrane | RT | 3 | 4.1E-4 | 9.2E1 | 7.1E-2 |

Functional annotation terms:

| Category | Term | RT | Genes | Count | LT | PH | PT | % | P-Value | Fold Enrichment | Benjamini |
|---|---|---|---|---|---|---|---|---|---|---|---|
| UP_KEYWORDS | Acetylation | RT | | 60 | 126 | 3424 | 20581 | 47.2 | 1.6E-15 | 2.9 | 2.8E-13 |
| UP_KEYWORDS | Mitochondrion | RT | | 31 | 126 | 1119 | 20581 | 24.4 | 3.7E-12 | 4.5 | 3.2E-10 |
| INTERPRO | Tim10/DDP family zinc finger | RT | | 6 | 113 | 6 | 18559 | 4.7 | 4.4E-11 | 164.2 | 8.5E-9 |
| UP_SEQ_FEATURE | short sequence motif:Twin CX3C motif | RT | | 6 | 124 | 6 | 20063 | 4.7 | 4.8E-11 | 161.8 | 1.3E-8 |
| UP_KEYWORDS | Mitochondrion inner membrane | RT | | 16 | 126 | 270 | 20581 | 12.6 | 9.9E-11 | 9.7 | 5.7E-9 |
| GOTERM_CC_DIRECT | mitochondrion | RT | | 31 | 118 | 1331 | 18224 | 24.4 | 8.3E-10 | 3.6 | 8.5E-8 |
| GOTERM_CC_DIRECT | mitochondrial intermembrane space | RT | | 10 | 118 | 74 | 18224 | 7.9 | 1.1E-9 | 20.9 | 8.5E-8 |
| GOTERM_CC_DIRECT | mitochondrial inner membrane | RT | | 18 | 118 | 441 | 18224 | 14.2 | 3.2E-9 | 6.3 | 1.7E-7 |
| KEGG_PATHWAY | Oxidative phosphorylation | RT | | 11 | 42 | 133 | 6879 | 8.7 | 3.5E-9 | 13.5 | 2.6E-7 |
| GOTERM_CC_DIRECT | mitochondrial intermembrane space protein transporter complex | RT | | 5 | 118 | 5 | 18224 | 3.9 | 8.0E-9 | 154.4 | 3.2E-7 |
| GOTERM_BP_DIRECT | chaperone-mediated protein transport | RT | | 5 | 110 | 8 | 16792 | 3.9 | 1.2E-7 | 95.4 | 6.0E-5 |
| UP_KEYWORDS | Chaperone | RT | | 11 | 126 | 201 | 20581 | 8.7 | 4.2E-7 | 8.9 | 1.8E-5 |
| KEGG_PATHWAY | Huntington's disease | RT | | 10 | 42 | 192 | 6879 | 7.9 | 1.4E-6 | 8.5 | 5.2E-5 |
| GOTERM_BP_DIRECT | protein targeting to mitochondrion | RT | | 6 | 110 | 34 | 16792 | 4.7 | 2.5E-6 | 26.9 | 6.5E-4 |
| GOTERM_CC_DIRECT | mediator complex | RT | | 6 | 118 | 35 | 18224 | 4.7 | 2.8E-6 | 26.5 | 8.9E-5 |
| UP_KEYWORDS | Translocation | RT | | 7 | 126 | 84 | 20581 | 5.5 | 1.2E-5 | 13.6 | 4.2E-4 |
| KEGG_PATHWAY | Parkinson's disease | RT | | 8 | 42 | 142 | 6879 | 6.3 | 1.7E-5 | 9.2 | 4.3E-4 |
| UP_KEYWORDS | Transit peptide | RT | | 14 | 126 | 536 | 20581 | 11.0 | 2.3E-5 | 4.3 | 6.8E-4 |
| UP_KEYWORDS | Transport | RT | | 28 | 126 | 1978 | 20581 | 22.0 | 4.6E-5 | 2.3 | 1.1E-3 |
| KEGG_PATHWAY | Alzheimer's disease | RT | | 8 | 42 | 168 | 6879 | 6.3 | 5.1E-5 | 7.8 | 9.6E-4 |

# Figure S8 – TMT iii.

### Functional annotation clustering:

### Functional annotation terms:

## SP4-GB / cSP3 80% ACN proteins (n=213):

| Category | Term | RT | Count | P_Value | Benjamini |
|---|---|---|---|---|---|
| **Annotation Cluster 1** | **Enrichment Score: 6.48** | | **Count** | **P_Value** | **Benjamini** |
| KEGG_PATHWAY | Oxidative phosphorylation | RT | 16 | 7.2E-11 | 1.1E-8 |
| UP_KEYWORDS | Mitochondrion inner membrane | RT | 19 | 4.3E-10 | 1.1E-7 |
| KEGG_PATHWAY | Parkinson's disease | RT | 15 | 2.0E-9 | 1.5E-7 |
| UP_KEYWORDS | Mitochondrion | RT | 33 | 1.5E-7 | 1.3E-5 |
| GOTERM_CC_DIRECT | mitochondrial respiratory chain complex I | RT | 8 | 1.0E-6 | 2.3E-4 |
| UP_KEYWORDS | Ubiquinone | RT | 7 | 1.4E-6 | 8.8E-5 |
| UP_KEYWORDS | Electron transport | RT | 10 | 1.8E-6 | 9.0E-5 |
| GOTERM_CC_DIRECT | mitochondrial inner membrane | RT | 19 | 1.9E-6 | 2.3E-4 |
| UP_KEYWORDS | Respiratory chain | RT | 8 | 3.8E-6 | 1.6E-4 |
| GOTERM_MF_DIRECT | NADH dehydrogenase (ubiquinone) activity | RT | 7 | 1.3E-5 | 4.1E-3 |
| GOTERM_BP_DIRECT | mitochondrial electron transport, NADH to ubiquinone | RT | 7 | 1.4E-5 | 1.3E-2 |
| GOTERM_BP_DIRECT | mitochondrial respiratory chain complex I assembly | RT | 7 | 5.9E-5 | 2.7E-2 |
| **Annotation Cluster 2** | **Enrichment Score: 4.04** | | **Count** | **P_Value** | **Benjamini** |
| KEGG_PATHWAY | Huntington's disease | RT | 12 | 2.6E-5 | 1.0E-3 |
| KEGG_PATHWAY | Non-alcoholic fatty liver disease (NAFLD) | RT | 10 | 1.1E-4 | 3.4E-3 |
| KEGG_PATHWAY | Alzheimer's disease | RT | 10 | 2.5E-4 | 6.4E-3 |
| **Annotation Cluster 3** | **Enrichment Score: 2.31** | | **Count** | **P_Value** | **Benjamini** |
| GOTERM_CC_DIRECT | oligosaccharyltransferase complex | RT | 4 | 1.5E-4 | 9.2E-3 |
| KEGG_PATHWAY | N-Glycan biosynthesis | RT | 5 | 3.3E-3 | 7.2E-2 |

| Category | Term | RT | Count | LT | PH | PT | % | P-Value | Fold Enrichment | Benjamini |
|---|---|---|---|---|---|---|---|---|---|---|
| KEGG_PATHWAY | Oxidative phosphorylation | RT | 16 | 88 | 133 | 6879 | 7.5 | 7.2E-11 | 9.4 | 1.1E-8 |
| UP_KEYWORDS | Mitochondrion inner membrane | RT | 19 | 213 | 270 | 20581 | 8.9 | 4.3E-10 | 6.8 | 1.1E-7 |
| KEGG_PATHWAY | Parkinson's disease | RT | 15 | 88 | 142 | 6879 | 7.0 | 2.0E-9 | 8.3 | 1.5E-7 |
| UP_KEYWORDS | Leber hereditary optic neuropathy | RT | 6 | 213 | 10 | 20581 | 2.8 | 2.7E-8 | 58.0 | 3.4E-6 |
| UP_KEYWORDS | Mitochondrion | RT | 33 | 213 | 1119 | 20581 | 15.5 | 1.5E-7 | 2.8 | 1.3E-5 |
| KEGG_PATHWAY | Metabolic pathways | RT | 36 | 88 | 1219 | 6879 | 16.9 | 5.9E-7 | 2.3 | 3.0E-5 |
| GOTERM_CC_DIRECT | mitochondrial respiratory chain complex I | RT | 8 | 201 | 49 | 18224 | 3.8 | 1.0E-6 | 14.8 | 2.3E-4 |
| UP_KEYWORDS | Ubiquinone | RT | 7 | 213 | 35 | 20581 | 3.3 | 1.4E-6 | 19.3 | 8.8E-5 |
| UP_KEYWORDS | Electron transport | RT | 10 | 213 | 108 | 20581 | 4.7 | 1.8E-6 | 8.9 | 9.0E-5 |
| GOTERM_CC_DIRECT | mitochondrial inner membrane | RT | 19 | 201 | 441 | 18224 | 8.9 | 1.9E-6 | 3.9 | 2.3E-4 |
| UP_KEYWORDS | Respiratory chain | RT | 8 | 213 | 63 | 20581 | 3.8 | 3.8E-6 | 12.3 | 1.6E-4 |
| UP_KEYWORDS | Transport | RT | 43 | 213 | 1978 | 20581 | 20.2 | 4.5E-6 | 2.1 | 1.6E-4 |
| GOTERM_MF_DIRECT | NADH dehydrogenase (ubiquinone) activity | RT | 7 | 186 | 48 | 16881 | 3.3 | 1.3E-5 | 13.2 | 4.1E-3 |
| GOTERM_BP_DIRECT | mitochondrial electron transport, NADH to ubiquinone | RT | 7 | 182 | 49 | 16792 | 3.3 | 1.4E-5 | 13.2 | 1.3E-2 |
| KEGG_PATHWAY | Huntington's disease | RT | 12 | 88 | 192 | 6879 | 5.6 | 2.6E-5 | 4.9 | 1.0E-3 |
| GOTERM_CC_DIRECT | endoplasmic reticulum membrane | RT | 25 | 201 | 862 | 18224 | 11.7 | 2.7E-5 | 2.6 | 2.2E-3 |
| UP_KEYWORDS | Endoplasmic reticulum | RT | 27 | 213 | 1067 | 20581 | 12.7 | 4.2E-5 | 2.4 | 1.3E-3 |
| GOTERM_BP_DIRECT | mitochondrial respiratory chain complex I assembly | RT | 7 | 182 | 63 | 16792 | 3.3 | 5.9E-5 | 10.3 | 2.7E-2 |
| KEGG_PATHWAY | Non-alcoholic fatty liver disease (NAFLD) | RT | 10 | 88 | 151 | 6879 | 4.7 | 1.1E-4 | 5.2 | 3.4E-3 |
| GOTERM_CC_DIRECT | oligosaccharyltransferase complex | RT | 4 | 201 | 10 | 18224 | 1.9 | 1.5E-4 | 36.3 | 9.2E-3 |
| GOTERM_MF_DIRECT | protein binding | RT | 122 | 186 | 8785 | 16881 | 57.3 | 1.5E-4 | 1.3 | 2.3E-2 |
| KEGG_PATHWAY | Alzheimer's disease | RT | 10 | 88 | 168 | 6879 | 4.7 | 2.5E-4 | 4.7 | 6.4E-3 |
| GOTERM_CC_DIRECT | mitochondrion | RT | 30 | 201 | 1331 | 18224 | 14.1 | 3.0E-4 | 2.0 | 1.5E-2 |

## SP4-GB / cSP3 50% ACN proteins (n=246):

| Category | Term | RT | Count | P_Value | Benjamini |
|---|---|---|---|---|---|
| **Annotation Cluster 1** | **Enrichment Score: 11.83** | | **Count** | **P_Value** | **Benjamini** |
| KEGG_PATHWAY | Oxidative phosphorylation | RT | 23 | 1.6E-17 | 2.2E-15 |
| KEGG_PATHWAY | Huntington's disease | RT | 25 | 3.7E-16 | 2.4E-14 |
| KEGG_PATHWAY | Parkinson's disease | RT | 21 | 1.5E-14 | 6.6E-13 |
| UP_KEYWORDS | Mitochondrion inner membrane | RT | 23 | 1.3E-12 | 1.7E-10 |
| GOTERM_CC_DIRECT | mitochondrial inner membrane | RT | 28 | 9.2E-12 | 2.7E-9 |
| KEGG_PATHWAY | Alzheimer's disease | RT | 18 | 3.8E-10 | 1.2E-8 |
| UP_KEYWORDS | Mitochondrion | RT | 40 | 1.2E-9 | 1.0E-7 |
| KEGG_PATHWAY | Non-alcoholic fatty liver disease (NAFLD) | RT | 15 | 4.8E-8 | 1.3E-6 |
| **Annotation Cluster 2** | **Enrichment Score: 6** | | **Count** | **P_Value** | **Benjamini** |
| GOTERM_MF_DIRECT | cytochrome-c oxidase activity | RT | 8 | 7.3E-8 | 2.4E-5 |
| GOTERM_BP_DIRECT | mitochondrial electron transport, cytochrome c to oxygen | RT | 7 | 1.2E-7 | 1.2E-4 |
| GOTERM_BP_DIRECT | hydrogen ion transmembrane transport | RT | 9 | 9.1E-7 | 4.5E-4 |
| KEGG_PATHWAY | Cardiac muscle contraction | RT | 8 | 1.3E-4 | 2.4E-3 |
| **Annotation Cluster 3** | **Enrichment Score: 5.09** | | **Count** | **P_Value** | **Benjamini** |
| INTERPRO | Tim10/DDP family zinc finger | RT | 6 | 1.4E-9 | 5.7E-7 |
| UP_SEQ_FEATURE | short sequence motif:Twin CX3C motif | RT | 6 | 1.4E-9 | 9.6E-7 |
| GOTERM_CC_DIRECT | mitochondrial intermembrane space protein transporter complex | RT | 5 | 1.2E-7 | 1.1E-5 |
| GOTERM_BP_DIRECT | chaperone-mediated protein transport | RT | 5 | 1.6E-6 | 5.4E-4 |
| UP_KEYWORDS | Translocation | RT | 7 | 4.8E-4 | 1.3E-2 |

| Category | Term | RT | Count | LT | PH | PT | % | P-Value | Fold Enrichment | Benjamini |
|---|---|---|---|---|---|---|---|---|---|---|
| KEGG_PATHWAY | Oxidative phosphorylation | RT | 23 | 105 | 133 | 6879 | 9.3 | 1.6E-17 | 11.3 | 2.2E-15 |
| UP_KEYWORDS | Acetylation | RT | 96 | 244 | 3424 | 20581 | 39.0 | 2.6E-17 | 2.4 | 7.0E-15 |
| KEGG_PATHWAY | Huntington's disease | RT | 25 | 105 | 192 | 6879 | 10.2 | 3.7E-16 | 8.5 | 2.4E-14 |
| KEGG_PATHWAY | Parkinson's disease | RT | 21 | 105 | 142 | 6879 | 8.5 | 1.5E-14 | 9.7 | 6.6E-13 |
| UP_KEYWORDS | Mitochondrion inner membrane | RT | 23 | 244 | 270 | 20581 | 9.3 | 1.3E-12 | 7.2 | 1.7E-10 |
| GOTERM_CC_DIRECT | mitochondrial inner membrane | RT | 28 | 228 | 441 | 18224 | 11.4 | 9.2E-12 | 5.1 | 2.7E-9 |
| KEGG_PATHWAY | Alzheimer's disease | RT | 18 | 105 | 168 | 6879 | 7.3 | 3.8E-10 | 7.0 | 1.2E-8 |
| UP_KEYWORDS | Mitochondrion | RT | 40 | 244 | 1119 | 20581 | 16.3 | 1.2E-9 | 3.0 | 1.0E-7 |
| INTERPRO | Tim10/DDP family zinc finger | RT | 6 | 222 | 6 | 18559 | 2.4 | 1.4E-9 | 83.6 | 5.7E-7 |
| UP_SEQ_FEATURE | short sequence motif:Twin CX3C motif | RT | 6 | 241 | 6 | 20063 | 2.4 | 1.4E-9 | 83.2 | 9.6E-7 |
| UP_KEYWORDS | Chaperone | RT | 16 | 244 | 201 | 20581 | 6.5 | 1.8E-8 | 6.7 | 1.2E-6 |
| GOTERM_CC_DIRECT | mitochondrial intermembrane space | RT | 11 | 228 | 74 | 18224 | 4.5 | 2.6E-8 | 11.9 | 3.9E-6 |
| KEGG_PATHWAY | Non-alcoholic fatty liver disease (NAFLD) | RT | 15 | 105 | 151 | 6879 | 6.1 | 4.8E-8 | 6.5 | 1.3E-6 |
| GOTERM_MF_DIRECT | cytochrome-c oxidase activity | RT | 8 | 214 | 30 | 16881 | 3.3 | 7.3E-8 | 21.0 | 2.4E-5 |
| GOTERM_CC_DIRECT | mitochondrial intermembrane space protein transporter complex | RT | 5 | 228 | 5 | 18224 | 2.0 | 1.2E-7 | 79.9 | 1.1E-5 |
| GOTERM_BP_DIRECT | mitochondrial electron transport, cytochrome c to oxygen | RT | 7 | 212 | 20 | 16792 | 2.8 | 1.2E-7 | 27.7 | 1.2E-4 |
| GOTERM_CC_DIRECT | mitochondrion | RT | 40 | 228 | 1331 | 18224 | 16.3 | 4.6E-7 | 2.4 | 3.4E-5 |
| UP_KEYWORDS | Electron transport | RT | 11 | 244 | 108 | 20581 | 4.5 | 6.2E-7 | 8.6 | 3.3E-5 |
| GOTERM_BP_DIRECT | hydrogen ion transmembrane transport | RT | 9 | 212 | 61 | 16792 | 3.7 | 9.1E-7 | 11.7 | 4.5E-4 |
| KEGG_PATHWAY | Metabolic pathways | RT | 40 | 105 | 1219 | 6879 | 16.3 | 1.2E-6 | 2.1 | 2.5E-5 |
| GOTERM_BP_DIRECT | chaperone-mediated protein transport | RT | 5 | 212 | 8 | 16792 | 2.0 | 1.6E-6 | 49.5 | 5.4E-4 |
| GOTERM_CC_DIRECT | mitochondrial respiratory chain complex I | RT | 8 | 228 | 49 | 18224 | 3.3 | 2.3E-6 | 13.0 | 1.4E-4 |
| UP_KEYWORDS | Respiratory chain | RT | 8 | 244 | 63 | 20581 | 3.3 | 9.2E-6 | 10.7 | 4.1E-4 |

## cSP3 (80+50 % ACN) / SP4-GB proteins (n=147, combined):

| Category | Term | RT | Count | P_Value | Benjamini |
|---|---|---|---|---|---|
| **Annotation Cluster 1** | **Enrichment Score: 3.26** | | **Count** | **P_Value** | **Benjamini** |
| INTERPRO | Intermediate filament protein, conserved site | RT | 6 | 1.1E-4 | 3.3E-2 |
| UP_SEQ_FEATURE | region of interest:Coil 2 | RT | 6 | 1.2E-4 | 2.4E-2 |
| UP_SEQ_FEATURE | region of interest:Linker 12 | RT | 6 | 1.2E-4 | 2.4E-2 |
| SMART | SM01391 | RT | 6 | 1.3E-4 | 9.0E-3 |
| UP_SEQ_FEATURE | region of interest:Coil 1A | RT | 6 | 1.8E-4 | 2.4E-2 |
| UP_SEQ_FEATURE | region of interest:Coil 1B | RT | 6 | 1.8E-4 | 2.4E-2 |
| UP_SEQ_FEATURE | region of interest:Linker 1 | RT | 6 | 1.8E-4 | 2.4E-2 |
| UP_SEQ_FEATURE | region of interest:Rod | RT | 6 | 1.9E-4 | 2.4E-2 |
| UP_KEYWORDS | Intermediate filament | RT | 6 | 2.1E-4 | 1.2E-2 |
| UP_SEQ_FEATURE | region of interest:Head | RT | 6 | 2.2E-4 | 2.4E-2 |
| UP_SEQ_FEATURE | region of interest:Tail | RT | 6 | 2.4E-4 | 2.4E-2 |
| INTERPRO | Intermediate filament protein | RT | 6 | 2.8E-4 | 4.2E-2 |

| Category | Term | RT | Count | LT | PH | PT | % | P-Value | Fold Enrichment | Benjamini |
|---|---|---|---|---|---|---|---|---|---|---|
| UP_KEYWORDS | Acetylation | RT | 46 | 145 | 3424 | 20581 | 31.3 | 1.1E-5 | 1.9 | 2.5E-3 |
| UP_KEYWORDS | Methylation | RT | 21 | 145 | 1001 | 20581 | 14.3 | 2.2E-5 | 3.0 | 2.6E-3 |
| UP_KEYWORDS | Phosphoprotein | RT | 82 | 145 | 8246 | 20581 | 55.8 | 6.0E-5 | 1.4 | 4.7E-3 |
| INTERPRO | Intermediate filament protein, conserved site | RT | 6 | 138 | 64 | 18559 | 4.1 | 1.1E-4 | 12.6 | 3.3E-2 |
| UP_SEQ_FEATURE | region of interest:Coil 2 | RT | 6 | 143 | 68 | 20063 | 4.1 | 1.2E-4 | 12.4 | 2.4E-2 |
| UP_SEQ_FEATURE | region of interest:Linker 12 | RT | 6 | 143 | 68 | 20063 | 4.1 | 1.2E-4 | 12.4 | 2.4E-2 |
| SMART | SM01391 | RT | 6 | 67 | 75 | 10057 | 4.1 | 1.3E-4 | 12.0 | 9.0E-3 |
| UP_SEQ_FEATURE | region of interest:Coil 1A | RT | 6 | 143 | 74 | 20063 | 4.1 | 1.8E-4 | 11.4 | 2.4E-2 |
| UP_SEQ_FEATURE | region of interest:Linker 1 | RT | 6 | 143 | 74 | 20063 | 4.1 | 1.8E-4 | 11.4 | 2.4E-2 |
| UP_SEQ_FEATURE | region of interest:Coil 1B | RT | 6 | 143 | 74 | 20063 | 4.1 | 1.8E-4 | 11.4 | 2.4E-2 |
| UP_SEQ_FEATURE | region of interest:Rod | RT | 6 | 143 | 75 | 20063 | 4.1 | 1.9E-4 | 11.2 | 2.4E-2 |
| UP_KEYWORDS | Intermediate filament | RT | 6 | 145 | 77 | 20581 | 4.1 | 2.1E-4 | 11.1 | 1.2E-2 |
| GOTERM_MF_DIRECT | structural constituent of cytoskeleton | RT | 7 | 131 | 110 | 16881 | 4.8 | 2.1E-4 | 8.2 | 4.9E-2 |
| UP_SEQ_FEATURE | region of interest:Head | RT | 6 | 143 | 77 | 20063 | 4.1 | 2.2E-4 | 10.9 | 2.4E-2 |
| UP_SEQ_FEATURE | region of interest:Tail | RT | 6 | 143 | 79 | 20063 | 4.1 | 2.4E-4 | 10.7 | 2.4E-2 |
| INTERPRO | Intermediate filament protein | RT | 6 | 138 | 78 | 18559 | 4.1 | 2.8E-4 | 10.3 | 4.2E-2 |
| UP_KEYWORDS | Cytoplasm | RT | 53 | 145 | 4816 | 20581 | 36.1 | 3.9E-4 | 1.6 | 1.8E-2 |

## cSP3 80% ACN / cSP3 50% ACN proteins (n=220)

| Category | Term | RT | Count | P_Value | Benjamini |
|---|---|---|---|---|---|
| **Annotation Cluster 1** | **Enrichment Score: 7.78** | | **Count** | **P_Value** | **Benjamini** |
| KEGG_PATHWAY | Huntington's disease | RT | 18 | 8.4E-12 | 1.0E-9 |
| KEGG_PATHWAY | Oxidative phosphorylation | RT | 15 | 7.2E-11 | 4.4E-9 |
| KEGG_PATHWAY | Alzheimer's disease | RT | 15 | 1.7E-9 | 6.5E-8 |
| KEGG_PATHWAY | Parkinson's disease | RT | 14 | 2.1E-9 | 6.5E-8 |
| GOTERM_CC_DIRECT | mitochondrial inner membrane | RT | 22 | 1.4E-8 | 3.9E-6 |
| KEGG_PATHWAY | Non-alcoholic fatty liver disease (NAFLD) | RT | 12 | 4.4E-7 | 1.1E-5 |
| KEGG_PATHWAY | Metabolic pathways | RT | 21 | 2.6E-2 | 3.5E-1 |
| **Annotation Cluster 2** | **Enrichment Score: 4.81** | | **Count** | **P_Value** | **Benjamini** |
| INTERPRO | Tim10/DDP family zinc finger | RT | 6 | 7.2E-10 | 2.5E-7 |
| UP_SEQ_FEATURE | short sequence motif:Twin CX3C motif | RT | 6 | 7.7E-10 | 7.3E-7 |
| GOTERM_BP_DIRECT | chaperone-mediated protein transport | RT | 6 | 7.9E-9 | 6.6E-6 |
| GOTERM_CC_DIRECT | mitochondrial intermembrane space protein transporter complex | RT | 5 | 6.6E-8 | 9.1E-6 |
| UP_KEYWORDS | Translocation | RT | 8 | 2.9E-5 | 1.4E-3 |
| GOTERM_BP_DIRECT | protein targeting to mitochondrion | RT | 6 | 3.1E-5 | 1.2E-2 |

| Category | Term | RT | Count | LT | PH | PT | % | P-Value | Fold Enrichment | Benjamini |
|---|---|---|---|---|---|---|---|---|---|---|
| UP_KEYWORDS | Acetylation | RT | 87 | 217 | 3424 | 20581 | 39.5 | 3.4E-16 | 2.4 | 7.9E-14 |
| KEGG_PATHWAY | Huntington's disease | RT | 18 | 74 | 192 | 6879 | 8.2 | 8.4E-12 | 8.7 | 1.0E-9 |
| KEGG_PATHWAY | Oxidative phosphorylation | RT | 15 | 74 | 133 | 6879 | 6.8 | 7.2E-11 | 10.5 | 4.4E-9 |
| INTERPRO | Tim10/DDP family zinc finger | RT | 6 | 196 | 6 | 18559 | 2.7 | 7.2E-10 | 94.7 | 2.5E-7 |
| UP_SEQ_FEATURE | short sequence motif:Twin CX3C motif | RT | 6 | 214 | 6 | 20063 | 2.7 | 7.7E-10 | 93.8 | 7.3E-7 |
| KEGG_PATHWAY | Alzheimer's disease | RT | 15 | 74 | 168 | 6879 | 6.8 | 1.7E-9 | 8.3 | 6.5E-8 |
| KEGG_PATHWAY | Parkinson's disease | RT | 14 | 74 | 142 | 6879 | 6.4 | 2.1E-9 | 9.2 | 6.5E-8 |
| GOTERM_BP_DIRECT | chaperone-mediated protein transport | RT | 6 | 184 | 8 | 16792 | 2.7 | 7.9E-9 | 68.4 | 6.6E-6 |
| GOTERM_CC_DIRECT | mitochondrial inner membrane | RT | 22 | 198 | 441 | 18224 | 10.0 | 1.4E-8 | 4.6 | 3.9E-6 |
| UP_KEYWORDS | Mitochondrion | RT | 35 | 217 | 1119 | 20581 | 15.9 | 2.2E-8 | 3.0 | 2.6E-6 |
| GOTERM_CC_DIRECT | mitochondrial intermembrane space protein transporter complex | RT | 5 | 198 | 5 | 18224 | 2.3 | 6.6E-8 | 92.0 | 9.1E-6 |
| GOTERM_CC_DIRECT | mitochondrial intermembrane space | RT | 10 | 198 | 74 | 18224 | 4.5 | 1.0E-7 | 12.4 | 9.3E-6 |
| UP_KEYWORDS | Mitochondrion inner membrane | RT | 16 | 217 | 270 | 20581 | 7.3 | 1.8E-7 | 5.6 | 1.3E-5 |
| UP_KEYWORDS | Chaperone | RT | 14 | 217 | 201 | 20581 | 6.4 | 2.2E-7 | 6.6 | 1.3E-5 |
| KEGG_PATHWAY | Non-alcoholic fatty liver disease (NAFLD) | RT | 12 | 74 | 151 | 6879 | 5.5 | 4.4E-7 | 7.4 | 1.1E-5 |
| GOTERM_CC_DIRECT | mitochondrion | RT | 35 | 198 | 1331 | 18224 | 15.9 | 2.2E-6 | 2.4 | 1.5E-4 |
| INTERPRO | Ubiquitin | RT | 8 | 196 | 59 | 18559 | 3.6 | 2.7E-6 | 12.8 | 4.7E-4 |
| GOTERM_MF_DIRECT | protein binding | RT | 127 | 185 | 8785 | 16881 | 57.7 | 3.9E-6 | 1.3 | 1.0E-3 |
| INTERPRO | Prefoldin | RT | 6 | 196 | 26 | 18559 | 2.7 | 6.7E-6 | 21.9 | 7.6E-4 |
| SMART | UBQ | RT | 6 | 71 | 43 | 10057 | 2.7 | 1.1E-5 | 19.8 | 4.5E-4 |
| SMART | SM01391 | RT | 7 | 71 | 75 | 10057 | 3.2 | 1.3E-5 | 13.2 | 4.5E-4 |
| GOTERM_MF_DIRECT | cytochrome-c oxidase activity | RT | 6 | 185 | 30 | 16881 | 2.7 | 1.7E-5 | 18.2 | 2.1E-3 |
| UP_SEQ_FEATURE | region of interest:Head | RT | 8 | 214 | 77 | 20063 | 3.6 | 1.8E-5 | 9.7 | 6.6E-3 |
| UP_SEQ_FEATURE | region of interest:Tail | RT | 8 | 214 | 79 | 20063 | 3.6 | 2.1E-5 | 9.5 | 6.6E-3 |
| INTERPRO | Ubiquilin | RT | 4 | 196 | 6 | 18559 | 1.8 | 2.2E-5 | 63.1 | 1.9E-3 |
| UP_KEYWORDS | Translocation | RT | 8 | 217 | 84 | 20581 | 3.6 | 2.9E-5 | 9.0 | 1.4E-3 |
| GOTERM_BP_DIRECT | protein targeting to mitochondrion | RT | 6 | 184 | 34 | 16792 | 2.7 | 3.1E-5 | 16.1 | 1.2E-2 |
| GOTERM_CC_DIRECT | prefoldin complex | RT | 4 | 198 | 7 | 18224 | 1.8 | 4.2E-5 | 52.6 | 2.3E-3 |
| GOTERM_BP_DIRECT | hydrogen ion transmembrane transport | RT | 7 | 184 | 61 | 16792 | 3.2 | 5.2E-5 | 10.5 | 1.2E-2 |
| INTERPRO | Intermediate filament protein, conserved site | RT | 7 | 196 | 64 | 18559 | 3.2 | 5.6E-5 | 10.4 | 3.8E-3 |
| GOTERM_BP_DIRECT | mitochondrial electron transport, cytochrome c to oxygen | RT | 5 | 184 | 20 | 16792 | 2.3 | 5.8E-5 | 22.8 | 1.2E-2 |

# Figure S8 – TMT iv.

## Functional annotation clustering:

## Functional annotation terms:

SP4-GB / S-Trap proteins (n=265):

| Annotation Cluster 1 | Enrichment Score: 6.55 | | Count | P_Value | Fold Change | Benjamini |
|---|---|---|---|---|---|---|
| UP_KEYWORDS | Ribonucleoprotein | RT | 21 | 1.4E-9 | 5.6E0 | 2.0E-7 |
| GOTERM_CC_DIRECT | cytosolic large ribosomal subunit | RT | 12 | 1.6E-9 | 1.3E1 | 5.0E-7 |
| GOTERM_MF_DIRECT | structural constituent of ribosome | RT | 18 | 1.1E-8 | 5.9E0 | 4.0E-6 |
| KEGG_PATHWAY | Ribosome | RT | 16 | 1.4E-8 | 6.5E0 | 2.2E-6 |
| UP_KEYWORDS | Ribosomal protein | RT | 16 | 1.5E-8 | 6.8E0 | 1.5E-6 |
| GOTERM_BP_DIRECT | SRP-dependent cotranslational protein targeting to membrane | RT | 12 | 7.9E-8 | 9.1E0 | 4.0E-5 |
| GOTERM_BP_DIRECT | translation | RT | 18 | 1.0E-7 | 5.1E0 | 4.0E-5 |
| GOTERM_BP_DIRECT | nuclear-transcribed mRNA catabolic process, nonsense-mediated decay | RT | 13 | 1.1E-7 | 7.8E0 | 4.0E-5 |
| GOTERM_BP_DIRECT | viral transcription | RT | 12 | 4.8E-7 | 7.6E0 | 1.1E-4 |
| GOTERM_BP_DIRECT | translational initiation | RT | 13 | 5.1E-7 | 6.8E0 | 1.1E-4 |
| GOTERM_CC_DIRECT | ribosome | RT | 13 | 2.6E-6 | 5.8E0 | 3.5E-4 |
| GOTERM_BP_DIRECT | rRNA processing | RT | 14 | 1.0E-5 | 4.7E0 | 1.9E-3 |
| GOTERM_BP_DIRECT | cytoplasmic translation | RT | 5 | 3.8E-4 | 1.4E1 | 5.3E-2 |
| GOTERM_MF_DIRECT | poly(A) RNA binding | RT | 29 | 1.7E-3 | 1.9E0 | 2.0E-1 |
| **Annotation Cluster 2** | **Enrichment Score: 5.17** | | **Count** | **P_Value** | **Fold Change** | **Benjamini** |
| UP_KEYWORDS | Mitochondrion inner membrane | RT | 17 | 3.9E-7 | 4.9E0 | 2.8E-5 |
| UP_KEYWORDS | Mitochondrion | RT | 34 | 5.8E-6 | 2.4E0 | 3.3E-4 |
| GOTERM_CC_DIRECT | mitochondrial inner membrane | RT | 20 | 8.7E-6 | 3.4E0 | 6.7E-4 |
| GOTERM_CC_DIRECT | mitochondrion | RT | 36 | 1.0E-4 | 2.0E0 | 6.3E-3 |

| Category | Term | RT | Count | LT | PH | PT | % | P-Value | Fold Enrichment | Benjamini |
|---|---|---|---|---|---|---|---|---|---|---|
| UP_KEYWORDS | Acetylation | RT | 95 | 262 | 3424 | 20581 | 35.8 | 2.3E-14 | 2.2 | 6.6E-12 |
| UP_KEYWORDS | Ribonucleoprotein | RT | 21 | 262 | 296 | 20581 | 7.9 | 1.4E-9 | 5.6 | 2.0E-7 |
| GOTERM_CC_DIRECT | cytosolic large ribosomal subunit | RT | 12 | 246 | 68 | 18224 | 4.5 | 1.6E-9 | 13.1 | 5.0E-7 |
| GOTERM_MF_DIRECT | structural constituent of ribosome | RT | 18 | 232 | 222 | 16881 | 6.8 | 1.1E-8 | 5.9 | 4.0E-6 |
| KEGG_PATHWAY | Ribosome | RT | 16 | 124 | 136 | 6879 | 6.0 | 1.4E-8 | 6.5 | 2.2E-6 |
| UP_KEYWORDS | Ribosomal protein | RT | 16 | 262 | 185 | 20581 | 6.0 | 1.5E-8 | 6.8 | 1.5E-6 |
| GOTERM_BP_DIRECT | SRP-dependent cotranslational protein targeting to membrane | RT | 12 | 236 | 94 | 16792 | 4.5 | 7.9E-8 | 9.1 | 4.0E-5 |
| GOTERM_BP_DIRECT | translation | RT | 18 | 236 | 253 | 16792 | 6.8 | 1.0E-7 | 5.1 | 4.0E-5 |
| GOTERM_BP_DIRECT | nuclear-transcribed mRNA catabolic process, nonsense-mediated decay | RT | 13 | 236 | 119 | 16792 | 4.9 | 1.1E-7 | 7.8 | 4.0E-5 |
| UP_KEYWORDS | Mitochondrion inner membrane | RT | 17 | 262 | 270 | 20581 | 6.4 | 3.9E-7 | 4.9 | 2.8E-5 |
| GOTERM_BP_DIRECT | viral transcription | RT | 12 | 236 | 112 | 16792 | 4.5 | 4.8E-7 | 7.6 | 1.1E-4 |
| GOTERM_BP_DIRECT | translational initiation | RT | 13 | 236 | 137 | 16792 | 4.9 | 5.1E-7 | 6.8 | 1.1E-4 |
| GOTERM_CC_DIRECT | ribosome | RT | 13 | 246 | 166 | 18224 | 4.9 | 2.6E-6 | 5.8 | 3.5E-4 |
| GOTERM_CC_DIRECT | nucleoplasm | RT | 66 | 246 | 2784 | 18224 | 24.9 | 3.4E-6 | 1.8 | 3.5E-4 |
| UP_KEYWORDS | Mitochondrion | RT | 34 | 262 | 1119 | 20581 | 12.8 | 5.8E-6 | 2.4 | 3.3E-4 |
| GOTERM_CC_DIRECT | mitochondrial inner membrane | RT | 20 | 246 | 441 | 18224 | 7.5 | 8.7E-6 | 3.4 | 6.7E-4 |
| GOTERM_BP_DIRECT | rRNA processing | RT | 14 | 236 | 214 | 16792 | 5.3 | 1.0E-5 | 4.7 | 1.9E-3 |
| GOTERM_MF_DIRECT | protein binding | RT | 152 | 232 | 8785 | 16881 | 57.4 | 2.5E-5 | 1.3 | 4.5E-3 |
| GOTERM_BP_DIRECT | mRNA splicing, via spliceosome | RT | 13 | 236 | 222 | 16792 | 4.9 | 7.1E-5 | 4.2 | 1.1E-2 |
| UP_KEYWORDS | Phosphoprotein | RT | 136 | 262 | 8246 | 20581 | 51.3 | 8.3E-5 | 1.3 | 4.0E-3 |

S-Trap / SP4-GB proteins (n=185):

| Annotation Cluster 1 | Enrichment Score: 7.96 | | Count | P_Value | Fold Change | Benjamini |
|---|---|---|---|---|---|---|
| UP_SEQ_FEATURE | region of interest:Tail | RT | 13 | 4.1E-12 | 1.9E1 | 2.4E-9 |
| INTERPRO | Intermediate filament protein, conserved site | RT | 12 | 1.3E-11 | 2.0E1 | 4.3E-9 |
| UP_SEQ_FEATURE | region of interest:Linker 12 | RT | 12 | 1.6E-11 | 2.0E1 | 4.7E-9 |
| SMART | SM01391 | RT | 12 | 1.7E-11 | 1.9E1 | 1.4E-9 |
| UP_SEQ_FEATURE | region of interest:Coil 1A | RT | 12 | 4.2E-11 | 1.8E1 | 4.8E-9 |
| UP_SEQ_FEATURE | region of interest:Coil 1B | RT | 12 | 4.2E-11 | 1.8E1 | 4.8E-9 |
| UP_SEQ_FEATURE | region of interest:Linker 1 | RT | 12 | 4.2E-11 | 1.8E1 | 4.8E-9 |
| UP_SEQ_FEATURE | region of interest:Rod | RT | 12 | 4.9E-11 | 1.8E1 | 4.8E-9 |
| UP_KEYWORDS | Intermediate filament | RT | 12 | 5.0E-11 | 1.8E1 | 1.2E-8 |
| UP_SEQ_FEATURE | region of interest:Head | RT | 12 | 6.6E-11 | 1.8E1 | 5.5E-9 |
| INTERPRO | Intermediate filament protein | RT | 12 | 1.2E-10 | 1.7E1 | 2.0E-8 |
| UP_SEQ_FEATURE | region of interest:Coil 2 | RT | 11 | 3.7E-10 | 1.8E1 | 2.7E-8 |
| GOTERM_CC_DIRECT | intermediate filament | RT | 12 | 8.1E-9 | 1.1E1 | 1.7E-6 |
| UP_KEYWORDS | Palmoplantar keratoderma | RT | 7 | 2.6E-7 | 2.6E1 | 3.2E-5 |
| UP_KEYWORDS | Keratin | RT | 11 | 7.7E-7 | 8.4E0 | 6.2E-5 |
| GOTERM_MF_DIRECT | structural molecule activity | RT | 13 | 3.4E-6 | 5.6E0 | 8.7E-4 |
| INTERPRO | Type II keratin | RT | 6 | 4.9E-6 | 2.3E1 | 3.3E-4 |
| GOTERM_BP_DIRECT | epidermis development | RT | 8 | 1.2E-5 | 1.0E1 | 8.0E-3 |
| UP_SEQ_FEATURE | site:Stutter | RT | 6 | 1.4E-5 | 1.9E1 | 9.4E-4 |
| UP_KEYWORDS | Ectodermal dysplasia | RT | 6 | 2.8E-5 | 1.7E1 | 1.7E-3 |
| GOTERM_CC_DIRECT | keratin filament | RT | 8 | 4.0E-5 | 8.6E0 | 2.9E-3 |
| GOTERM_MF_DIRECT | structural constituent of cytoskeleton | RT | 8 | 7.5E-5 | 7.8E0 | 9.4E-3 |
| INTERPRO | Keratin, type I | RT | 5 | 2.2E-4 | 1.7E1 | 1.3E-2 |
| **Annotation Cluster 2** | **Enrichment Score: 3.16** | | **Count** | **P_Value** | **Fold Change** | **Benjamini** |
| SMART | SM01394 | RT | 7 | 8.0E-8 | 3.0E1 | 3.4E-6 |
| INTERPRO | S100/Calbindin-D9k, conserved site | RT | 7 | 8.0E-8 | 3.1E1 | 9.1E-6 |
| INTERPRO | S100/CaBP-9k-type, calcium binding, subdomain | RT | 7 | 2.1E-7 | 2.6E1 | 1.8E-5 |

| Category | Term | RT | Count | LT | PH | PT | % | P-Value | Fold Enrichment | Benjamini |
|---|---|---|---|---|---|---|---|---|---|---|
| UP_SEQ_FEATURE | region of interest:Tail | RT | 13 | 176 | 79 | 20063 | 7.1 | 4.1E-12 | 18.8 | 2.4E-9 |
| INTERPRO | Intermediate filament protein, conserved site | RT | 12 | 170 | 64 | 18559 | 6.5 | 1.3E-11 | 20.5 | 4.3E-9 |
| UP_SEQ_FEATURE | region of interest:Linker 12 | RT | 12 | 176 | 68 | 20063 | 6.5 | 1.6E-11 | 20.1 | 4.7E-9 |
| SMART | SM01391 | RT | 12 | 83 | 75 | 10057 | 6.5 | 1.7E-11 | 19.4 | 1.4E-9 |
| UP_SEQ_FEATURE | region of interest:Linker 1 | RT | 12 | 176 | 74 | 20063 | 6.5 | 4.2E-11 | 18.5 | 4.8E-9 |
| UP_SEQ_FEATURE | region of interest:Coil 1B | RT | 12 | 176 | 74 | 20063 | 6.5 | 4.2E-11 | 18.5 | 4.8E-9 |
| UP_SEQ_FEATURE | region of interest:Coil 1A | RT | 12 | 176 | 74 | 20063 | 6.5 | 4.2E-11 | 18.5 | 4.8E-9 |
| UP_SEQ_FEATURE | region of interest:Rod | RT | 12 | 176 | 75 | 20063 | 6.5 | 4.9E-11 | 18.2 | 4.8E-9 |
| UP_KEYWORDS | Intermediate filament | RT | 12 | 176 | 77 | 20581 | 6.5 | 5.0E-11 | 18.2 | 1.2E-8 |
| UP_SEQ_FEATURE | region of interest:Head | RT | 12 | 176 | 77 | 20063 | 6.5 | 6.6E-11 | 17.8 | 5.5E-9 |
| INTERPRO | Intermediate filament protein | RT | 12 | 170 | 78 | 18559 | 6.5 | 1.2E-10 | 16.8 | 2.0E-8 |
| UP_SEQ_FEATURE | region of interest:Coil 2 | RT | 11 | 176 | 68 | 20063 | 6.0 | 3.7E-10 | 18.4 | 2.7E-8 |
| GOTERM_CC_DIRECT | intermediate filament | RT | 12 | 170 | 113 | 18224 | 6.5 | 8.1E-9 | 11.4 | 1.7E-6 |
| SMART | SM01394 | RT | 7 | 83 | 28 | 10057 | 3.8 | 8.0E-8 | 30.3 | 3.4E-6 |
| INTERPRO | S100/Calbindin-D9k, conserved site | RT | 7 | 170 | 25 | 18559 | 3.8 | 8.0E-8 | 30.6 | 9.1E-6 |
| GOTERM_CC_DIRECT | extracellular exosome | RT | 54 | 170 | 2811 | 18224 | 29.3 | 1.4E-7 | 2.1 | 1.5E-5 |
| INTERPRO | S100/CaBP-9k-type, calcium binding, subdomain | RT | 7 | 170 | 29 | 18559 | 3.8 | 2.1E-7 | 26.4 | 1.8E-5 |
| UP_KEYWORDS | Palmoplantar keratoderma | RT | 7 | 176 | 32 | 20581 | 3.8 | 2.6E-7 | 25.6 | 3.2E-5 |
| UP_KEYWORDS | Keratin | RT | 11 | 176 | 153 | 20581 | 6.0 | 7.7E-7 | 8.4 | 6.2E-5 |
| GOTERM_MF_DIRECT | structural molecule activity | RT | 13 | 158 | 247 | 16881 | 7.1 | 3.4E-6 | 5.6 | 8.7E-4 |

SP4-GB / Spin filter proteins (n=102):

| Annotation Cluster 1 | Enrichment Score: 3.45 | | Count | P_Value | Fold Change | Benjamini |
|---|---|---|---|---|---|---|
| UP_KEYWORDS | Endoplasmic reticulum | RT | 18 | 1.6E-5 | 3.4E0 | 1.1E-3 |
| GOTERM_CC_DIRECT | endoplasmic reticulum membrane | RT | 15 | 2.0E-4 | 3.2E0 | 3.7E-2 |
| GOTERM_CC_DIRECT | endoplasmic reticulum | RT | 11 | 1.4E-2 | 2.4E0 | 3.5E-1 |
| **Annotation Cluster 2** | **Enrichment Score: 3.12** | | **Count** | **P_Value** | **Fold Change** | **Benjamini** |
| UP_KEYWORDS | ER-Golgi transport | RT | 7 | 6.9E-6 | 1.5E1 | 6.8E-4 |
| UP_KEYWORDS | Golgi apparatus | RT | 13 | 6.2E-4 | 3.2E0 | 2.4E-2 |
| GOTERM_BP_DIRECT | ER to Golgi vesicle-mediated transport | RT | 6 | 2.0E-3 | 6.7E0 | 3.5E-1 |
| GOTERM_CC_DIRECT | Golgi membrane | RT | 8 | 4.0E-2 | 2.5E0 | 4.8E-1 |
| **Annotation Cluster 3** | **Enrichment Score: 2.94** | | **Count** | **P_Value** | **Fold Change** | **Benjamini** |
| UP_SEQ_FEATURE | topological domain:Mitochondrial matrix | RT | 4 | 3.3E-4 | 2.9E1 | 9.6E-2 |
| UP_KEYWORDS | Mitochondrion inner membrane | RT | 7 | 2.2E-3 | 5.2E0 | 6.0E-2 |
| UP_SEQ_FEATURE | topological domain:Mitochondrial intermembrane | RT | 4 | 2.2E-3 | 1.5E1 | 3.1E-1 |
| **Annotation Cluster 4** | **Enrichment Score: 2.67** | | **Count** | **P_Value** | **Fold Change** | **Benjamini** |
| GOTERM_BP_DIRECT | phosphatidylinositol dephosphorylation | RT | 4 | 3.1E-4 | 3.0E1 | 8.3E-2 |
| GOTERM_MF_DIRECT | inositol-1,4,5-trisphosphate 5-phosphatase activity | RT | 3 | 4.2E-4 | 9.3E1 | 6.3E-2 |
| SMART | IPPc | RT | 3 | 4.6E-4 | 8.9E1 | 2.1E-2 |

| Category | Term | RT | Count | LT | PH | PT | % | P-Value | Fold Enrichment | Benjamini |
|---|---|---|---|---|---|---|---|---|---|---|
| UP_KEYWORDS | Transport | RT | 28 | 102 | 1978 | 20581 | 27.5 | 6.5E-7 | 2.9 | 1.3E-4 |
| UP_KEYWORDS | ER-Golgi transport | RT | 7 | 102 | 94 | 20581 | 6.9 | 6.9E-6 | 15.0 | 6.8E-4 |
| UP_KEYWORDS | Endoplasmic reticulum | RT | 18 | 102 | 1067 | 20581 | 17.6 | 1.6E-5 | 3.4 | 1.1E-3 |
| UP_KEYWORDS | Protein transport | RT | 13 | 102 | 610 | 20581 | 12.7 | 4.4E-5 | 4.3 | 2.2E-3 |
| GOTERM_BP_DIRECT | protein transport | RT | 11 | 94 | 395 | 16792 | 10.8 | 6.6E-5 | 5.0 | 3.6E-2 |
| GOTERM_CC_DIRECT | endoplasmic reticulum membrane | RT | 15 | 99 | 862 | 18224 | 14.7 | 2.0E-4 | 3.2 | 3.7E-2 |
| GOTERM_BP_DIRECT | phosphatidylinositol dephosphorylation | RT | 4 | 94 | 24 | 16792 | 3.9 | 3.1E-4 | 29.8 | 8.3E-2 |
| UP_SEQ_FEATURE | topological domain:Mitochondrial matrix | RT | 4 | 102 | 27 | 20063 | 3.9 | 3.3E-4 | 29.1 | 9.6E-2 |
| GOTERM_MF_DIRECT | inositol-1,4,5-trisphosphate 5-phosphatase activity | RT | 3 | 91 | 6 | 16881 | 2.9 | 4.2E-4 | 92.8 | 6.3E-2 |
| SMART | IPPc | RT | 3 | 34 | 10 | 10057 | 2.9 | 4.6E-4 | 88.7 | 2.1E-2 |
| GOTERM_CC_DIRECT | membrane | RT | 25 | 99 | 2200 | 18224 | 24.5 | 4.9E-4 | 2.1 | 4.1E-2 |
| UP_KEYWORDS | Golgi apparatus | RT | 13 | 102 | 812 | 20581 | 12.7 | 6.2E-4 | 3.2 | 2.4E-2 |
| GOTERM_CC_DIRECT | mitochondrion | RT | 18 | 99 | 1331 | 18224 | 17.6 | 6.9E-4 | 2.5 | 4.1E-2 |

S-Trap / Spin filter proteins (n=102):

| Annotation Cluster 1 | Enrichment Score: 5.39 | | Count | P_Value | Fold Change | Benjamini |
|---|---|---|---|---|---|---|
| UP_KEYWORDS | Endoplasmic reticulum | RT | 23 | 1.1E-6 | 3.3E0 | 1.9E-4 |
| GOTERM_CC_DIRECT | endoplasmic reticulum membrane | RT | 21 | 2.7E-6 | 3.4E0 | 2.8E-4 |
| GOTERM_CC_DIRECT | endoplasmic reticulum | RT | 19 | 2.2E-5 | 3.2E0 | 1.1E-3 |
| **Annotation Cluster 2** | **Enrichment Score: 3.23** | | **Count** | **P_Value** | **Fold Change** | **Benjamini** |
| UP_KEYWORDS | ER-Golgi transport | RT | 8 | 2.4E-6 | 1.3E1 | 1.9E-4 |
| GOTERM_BP_DIRECT | ER to Golgi vesicle-mediated transport | RT | 7 | 1.2E-3 | 5.9E0 | 7.9E-1 |
| GOTERM_BP_DIRECT | COPII vesicle coating | RT | 3 | 7.4E-2 | 6.7E0 | 1.0E0 |
| **Annotation Cluster 3** | **Enrichment Score: 3.05** | | **Count** | **P_Value** | **Fold Change** | **Benjamini** |
| SMART | SM01391 | RT | 6 | 2.7E-5 | 1.6E1 | 1.6E-3 |
| INTERPRO | Intermediate filament protein, conserved site | RT | 6 | 7.9E-5 | 1.3E1 | 2.1E-2 |
| UP_SEQ_FEATURE | region of interest:Linker 12 | RT | 6 | 8.5E-5 | 1.3E1 | 7.6E-3 |
| UP_SEQ_FEATURE | region of interest:Coil 1B | RT | 6 | 1.3E-4 | 1.2E1 | 7.6E-3 |
| UP_SEQ_FEATURE | region of interest:Linker 1 | RT | 6 | 1.3E-4 | 1.2E1 | 7.6E-3 |
| UP_SEQ_FEATURE | region of interest:Coil 1A | RT | 6 | 1.3E-4 | 1.2E1 | 7.6E-3 |
| UP_SEQ_FEATURE | region of interest:Rod | RT | 6 | 1.4E-4 | 1.2E1 | 7.6E-3 |

| Category | Term | RT | Count | LT | PH | PT | % | P-Value | Fold Enrichment | Benjamini |
|---|---|---|---|---|---|---|---|---|---|---|
| UP_KEYWORDS | Endoplasmic reticulum | RT | 23 | 133 | 1067 | 20581 | 16.8 | 1.1E-6 | 3.3 | 1.9E-4 |
| UP_KEYWORDS | Transport | RT | 32 | 133 | 1978 | 20581 | 23.4 | 2.1E-6 | 2.5 | 1.9E-4 |
| UP_KEYWORDS | ER-Golgi transport | RT | 8 | 133 | 94 | 20581 | 5.8 | 2.4E-6 | 13.2 | 1.9E-4 |
| GOTERM_CC_DIRECT | membrane | RT | 36 | 130 | 2200 | 18224 | 26.3 | 2.5E-6 | 2.3 | 2.8E-4 |
| GOTERM_CC_DIRECT | endoplasmic reticulum membrane | RT | 21 | 130 | 862 | 18224 | 15.3 | 2.7E-6 | 3.4 | 2.8E-4 |
| GOTERM_CC_DIRECT | extracellular exosome | RT | 41 | 130 | 2811 | 18224 | 29.9 | 6.3E-6 | 2.0 | 4.3E-4 |
| UP_KEYWORDS | Mitochondrion inner membrane | RT | 11 | 133 | 270 | 20581 | 8.0 | 9.8E-6 | 6.3 | 5.6E-4 |
| UP_SEQ_FEATURE | topological domain:Mitochondrial intermembrane | RT | 6 | 133 | 51 | 20063 | 4.4 | 2.1E-5 | 17.7 | 5.5E-3 |
| GOTERM_CC_DIRECT | endoplasmic reticulum | RT | 19 | 130 | 828 | 18224 | 13.9 | 2.2E-5 | 3.2 | 1.1E-3 |
| SMART | SM01391 | RT | 6 | 49 | 75 | 10057 | 4.4 | 2.7E-5 | 16.4 | 1.6E-3 |
| UP_SEQ_FEATURE | topological domain:Mitochondrial matrix | RT | 5 | 133 | 27 | 20063 | 3.6 | 2.8E-5 | 27.9 | 5.5E-3 |
| GOTERM_CC_DIRECT | mitochondrion | RT | 24 | 130 | 1331 | 18224 | 17.5 | 5.5E-5 | 2.5 | 2.3E-3 |
| UP_KEYWORDS | Golgi apparatus | RT | 17 | 133 | 812 | 20581 | 12.4 | 6.5E-5 | 3.2 | 3.0E-3 |
| INTERPRO | Intermediate filament protein, conserved site | RT | 6 | 129 | 64 | 18559 | 4.4 | 7.9E-5 | 13.5 | 2.1E-2 |
| UP_SEQ_FEATURE | region of interest:Linker 12 | RT | 6 | 133 | 68 | 20063 | 4.4 | 8.5E-5 | 13.3 | 7.6E-3 |
| GOTERM_CC_DIRECT | endoplasmic reticulum-Golgi intermediate compartment | RT | 6 | 130 | 68 | 18224 | 4.4 | 1.2E-4 | 12.4 | 4.1E-3 |
| UP_SEQ_FEATURE | region of interest:Linker 1 | RT | 6 | 133 | 74 | 20063 | 4.4 | 1.3E-4 | 12.2 | 7.6E-3 |
| UP_SEQ_FEATURE | region of interest:Coil 1A | RT | 6 | 133 | 74 | 20063 | 4.4 | 1.3E-4 | 12.2 | 7.6E-3 |
| UP_SEQ_FEATURE | region of interest:Coil 1B | RT | 6 | 133 | 74 | 20063 | 4.4 | 1.3E-4 | 12.2 | 7.6E-3 |
| UP_KEYWORDS | Transmembrane helix | RT | 57 | 133 | 5634 | 20581 | 41.6 | 1.3E-4 | 1.6 | 4.2E-3 |

**Figure S8. DAVID-derived term enrichment and clustering for those proteins observed more significantly recovered by SP3 and SP4 by TMT quantification (Fig 2D).** The human Swissprot UniProt proteome was used as a background. See also: Fig 2F, a summary of a more concise, GO-SLIM analysis of the cellular compartment terms for these protein subsets.

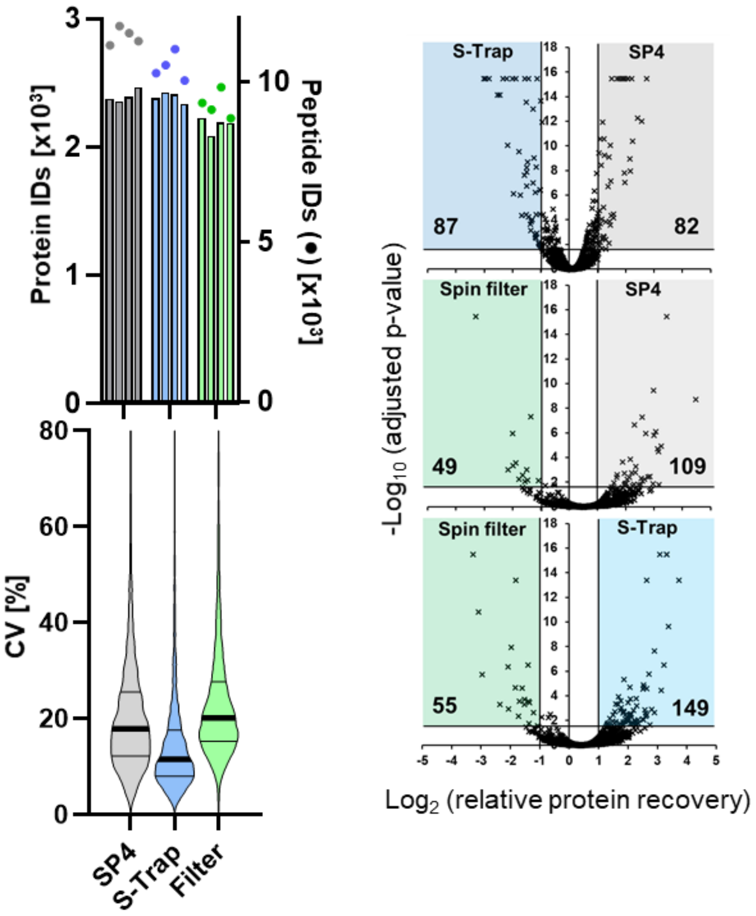


**Figure S9. A label-free comparison of proteomics preparations by SP4, S-Trap, and precipitate capture by 0.22 $\mu$m nylon spin filters.** Protein (bars) and peptide (dots) identifications, protein coefficients of variance distributions (violin plot, thick bar – median, thin bars - quartiles) and volcano plots describing significant differential recovery for 100 $\mu$g of HEK293 lysate prepared by SP4, S-Trap, and precipitate capture by 0.22 $\mu$m nylon spin filters. See also: Fig 2-iv, a higher-depth proteome characterisation by TMT.

## Supplementary Methods

**Materials.** Dulbecco's Modified Eagle Medium (DMEM), 100x pen-strep, 100x L-glutamine, 100x MEM non-essential amino acids (NEAA), and UltraPure Tris were purchased from Invitrogen; HyClone foetal bovine serum (FBS) from Fisher Scientific; cOmplete mini EDTA-free protease inhibitors from Roche; BCA and peptide quantitation assays, TMT 6-plex, and LC-MS grade ACN from Thermo Scientific; HEPES from Melford Laboratories; NP-40 from Biovision; NaCl and urea from VWR International; glycerol, NaOH and LC-grade ACN from Fisher; SpeedBeads magnetic carboxylate modified particles 45152105050250 and 65152105050250 from Cytiva (GE Healthcare); Protein LoBind tubes from Eppendorf; Trypsin (V5111) and Lys-C from Promega, Costar Spin-X 0.22 $\mu$m nylon centrifugal filters from Corning; and S-Trap mini columns from Protifi. All other reagents were purchased from Sigma-Aldrich. Additional reagents used for validation are described for each lab.

**Cell culture and lysis.** HEK293 cells were grown in DMEM supplemented with 10% fetal bovine serum with 1x pen-strep, L-glutamine, and NEAA, in a humidified incubator set at 37 °C and 5% $CO_2$. Cells were grown to 70–80% confluency and were washed twice with phosphate buffered saline. Cells were harvested by scraping, pelleted at 300*g* for 5 min and snap-frozen in liquid nitrogen.

Detergent-based lysis was performed resuspending snap-frozen cell pellets in 'SP3 lysis buffer' (50 mM HEPES pH 8.0, 1% SDS, 1% Triton X-100, 1% NP-40, 1% Tween 20, 1% sodium deoxycholate, 50 mM NaCl, 10 mM DTT, 5 mM EDTA, 1% (w/v) glycerol, 1× cOmplete protease inhibitor tablet, 40 mM 2-chloroacetamide (CAA)). Lysis was conducted by trituration with a 23-gauge needle, incubated at 95 °C for 5 min, cooled to RT for 10 min, sonicated on ice for 12× 5 s bursts with 5 s intervals and cleared at 16,000*g* for 10 min at 4 °C. Protein concentration was estimated by BCA assay, Pierce 660nm Protein Assay, or NanoDrop 2000 (Thermo Scientific) at 280 nm.

Urea-based lysis was performed in-flask with urea buffer (8 M Urea, 50 mM Tris-HCl pH 8.0, 75 mM NaCl, 1 mM EDTA, 1× cOmplete protease inhibitor) and lysate snap-frozen in liquid nitrogen. Defrosted lysate was triturated 20× on ice and cleared at 16,000*g* for 10 min at 4 °C. Protein concentration was estimated by BCA assay according to manufacturer's instructions. Protein was reduced using 5 mM DTT for 45 min at 25 °C and alkylated with 10 mM CAA for 45 min at 25 °C.

'SPEED' lysis was performed as described previously (1). Briefly, an aliquoted cell pellet was lysed in 100% TFA, neutralised in 2 M Tris, and reduced and alkylated with 10 mM DTT and 40 mM CAA. The lysate was diluted 1:1 with water and precipitated according the SP4 protocol below with 10:1 glass bead:protein ratio.

**Preparation of complex lysates.** Whole tissue homogenates of mouse heart and lungs were prepared from 3× washed organs on ice using a gentleMACS tissue dissociator (Miltenyi Biotec) in PBS to single-cell suspensions using the recommended setting for each organ and centrifuged at 300*g* for 3 min at 4 °C. Pellets were lysed in SP3 lysis buffer and handled as described above, using additional sonication and no trituration. FFPE cross sections were incubated twice in xylene for 2 min, washed sequentially with 90, 70, 50, 70, 90, and 100% ethanol, incubated in xylene for 5 min and air dried. Tissue was solubilised in SP3 lysis buffer using a Bioruptor sonicator (Diagenode) for 22.5 min (15 cycles: 1 min on, 30 s off) at the highest setting at 4 °C. Lysates were incubated for 1 h at 99 °C. The samples were returned to the Bioruptor and the 1 h incubation was repeated. Lysates were cleared at 16,000*g* for 5 min and the supernatant was quantitated, reduced and alkylated, as above, for SP3 and SP4 processing. For whole *Drosophila melanogaster* homogenates, adult $w^{1118}$ flies were transferred to a 1.5 mL microcentrifuge tube and immobilized by placing them at 4 °C for 5 min. The flies were then solubilised in ice-cold SP3 lysis buffer using a 1.5 mL tube-compatible pestle (Bel-Art, F65000-0006) and the lysate cleared twice at 16,000*g* for 10 min at 4 °C. Lysates were handled as above for SP3 and SP4.

**Bead preparation.** SpeedBeads magnetic carboxylate-modified particles (catalogue no. 45152105050250 and 65152105050250) were mixed 1:1, washed 3 times using Milli-Q® water, and resuspended at 50 mg/mL.

Silica beads/glass spheres (9–13 $\mu$m mean particle diameter, catalogue no. 440345) were suspended at an initial concentration of 100 mg/mL in Milli-Q water, washed with 100% ACN and 100 mM ammonium bicarbonate (ABC), and at least twice more with water. With each wash, the beads were pelleted by centrifugation at 16,000*g* for 1 min and the supernatant discarded. Of note: approximately 50% of the beads were buoyant and did not pellet, and were carefully removed over the course of these wash steps. Metal filings were noted to be a contaminant in the beads and can either be removed by magnet or acid wash, but did not impact any analyses. The beads were then resuspended in the initial suspension volume at 50 mg/mL (given ~50% were retained) in Milli-Q water. Alternatively, the beads were resuspended in 100% ACN at a concentration of at least 2.5× the protein concentration (detailed in the protocol) to allow simultaneous addition of beads and ACN, ensuring uniform bead suspension during aggregation and removing the need to add additional volumes.

**SP3/SP4 protein aggregation/precipitation.** Lysates were aliquoted into Protein LoBind tubes for each method and replicate. For SP4, 0.5 mL tubes were used (where volumes allowed) to give the densest pellet. Either 10:1 bead:protein ratio, or the equivalent volume of Milli-Q water (for the bead-free experiments, to maintain consistent concentrations), was added to lysates and gently vortex-mixed (< 500 rpm). Samples were handled such that liquid volume was minimised. 100% ACN was added (without pipette mixing) to a final concentration of 80% and tubes gently vortexed for 5 s. SP3 samples were incubated at 25 °C for 5 min at 800 rpm on a Thermomixer Comfort and placed on a magnetic rack for 2 min. SP4 samples were centrifuged for 5 min at 16,000*g*. Supernatants were removed carefully, using the tube hinge to orientate pellets. Three wash steps were performed with at least twice the total precipitation reaction volume of 80% ethanol, with buffer added slowly and avoiding disturbing the beads/pellet. Each wash used either a 2 min magnetic separation (SP3) or 2 min centrifugation at 16,000*g* (SP4).

For centrifugal SP3, the SP3 protocol was followed replacing magnetic isolation with 5 min and 2 min centrifugation steps at 16,000*g*.

**Proteolysis and peptide isolation.** After the final wash, remaining supernatant (bar < 5 $\mu$L) was carefully removed, the protein aggregates resuspended by gently vortexing the pellet in 100 mM ABC with 1:100 trypsin:protein ratio, and placed in a sonicator bath for 5 min. For the 500 and 5000 $\mu$g samples, 1:100 TrypZean (T3568, Sigma-Aldrich) was used in place of trypsin. Samples were incubated for 18 h at 37 °C at 1000 rpm on a Thermomixer Comfort. Peptide-containing supernatants were isolated by removal of magnetic beads (magrack, SP3) or beads and insoluble debris (16,000*g*, SP4) for 2 min.

**Peptide quantitation assay.** Peptide yields for optimization were determined using the Pierce Quantitative Fluorometric Peptide Assay (Thermo Scientific) according to manufacturer's instructions. For initial optimization, samples were prepared as above, varying acetonitrile concentration, bead:protein ratio, and centrifugation time while otherwise using 80% ACN, 5/2 min capture/wash centrifugation, and a 10:1 bead:protein ratio. Samples for each condition ($n$ = 4) were digested in 50 $\mu$L of 20 mM ABC, and 10 $\mu$L were analysed in triplicate. For evaluation of SP3 and SP4 protein input concentrations on peptide recovery, 50 $\mu$g of HEK293 protein was aliquoted ($n$ = 3), diluted from 5 to 0.63 $\mu$g/$\mu$L, and processed as described above.

**S-Trap, spin filter, and SP4 protein cleanup comparison.** To avoid experimental artefacts from buffer type and peripheral method differences, SP4 and centrifugal filters were adapted to follow the S-Trap procedure wherever possible. HEK293 lysate was prepared with 5% SDS, 50 mM triethylammonium bicarbonate (TEAB), sonicated as above, reduced with 5 mM TCEP for 15 min at 55 °C, and alkylated

with 20 mM CAA for 10 min. For S-Trap, the manufacturer's recommended protocol was followed: 100 µg was acidified, precipitated, and processed using S-Trap mini columns. For spin filtration, 20 µL (100 µg) of lysate in was applied to a pre-wetted nylon 0.22 µm spin filter and precipitated with 80 µL of ACN, precipitated captured and washed 3× with 80% ethanol at 6,000*g* for 1 min for all spins. For SP4-GB 80 µL of 12.5 µg/µL ACN–bead suspension was added to 20 µL (100 µg) of lysate and the described SP4 protocol followed. Digests were performed with 5 µg of trypsin and 2 µg of Lys-C in 125 µL of 50 mM TEAB for 2 h. Recommended S-Trap washes (80 µL of 50 mM TEAB, 0.2% FA, and 50% ACN) were used during all three methods, for the purposes of consistency. Though notably, this may have led to avoidable losses for SP4 from additional volumes and need for lyophilisation steps. Peptide solutions were lyophilised and reconstituted in 100 µL of 100 mM TEAB.

**TMT labelling.** For the 100 µg TMT experiments, 50 µL of 100 mM TEAB was used in place of ABC and both trypsin and Lys-C were added to a 1:100 enzyme:protein ratio. The resulting peptides were isolated by magnet or centrifugation and reaction vessels washed with 50 µL of 100 mM TEAB. 0.2 mg of TMT labeling reagent was added to each sample and incubated for 1 h at RT, and treated with 8 µL of 5% hydroxylamine for 15 min at RT. Labeled peptides were vacuum-concentrated, reconstituted, and pooled.

**Peptide pre-fractionation.** TMT-labeled peptides were reconstituted in 80 µL 3% (v/v) ACN + 0.1% (v/v) ammonium hydroxide and resolved using high-pH RP C18 chromatography (XBridge BEH 150 mm × 3 mm ID x 3.5 µm particle, Waters™, Milford, MA) at 0.3 mL/min with a Dionex UltiMate 3000 HPLC system (Thermo Scientific) at 30 °C. Mobile phases A (2% ACN + 0.1% ammonium hydroxide) and B (98% ACN + 0.1% ammonium hydroxide) were used for a gradient of: 0–20 min (3% B), 75 min (30% B), 105 min (85% B). 70 fractions were collected in a peak-dependent manner and individually lyophilized. Fractions at the extremes of the chromatogram were subjected to solid-phase extraction (SPE) and orthogonally concatenated, giving 62 fractions (TMT i) or 28 fractions (TMT ii–iv) for analysis.

**LC-MS analysis.** Label-free analyses of SP3, SP4-BF, and SP4-GB peptides were acquired using a Q-Exactive Plus Orbitrap MS (Thermo Scientific) coupled with a Dionex UltiMate 3000 nanoHPLC system (Thermo Scientific). Peptides were separated on a reversed-phase nanoLC column (150 mm × 0.075 mm; Reprosil-Pur C18AQ, Dr Maisch). For each analysis the equivalent of 100 ng peptides (as a proportion of protein input) were separated using a 120 min gradient of 5–35% ACN, 0.1% FA with a flow rate of ~300 nL/min.

Mass spectra were acquired with the following parameters for $MS^1$: resolution 70,000, scan range 350–1,800 m/z, automatic gain control (AGC) target $3{\times}10^6$, and maximum injection time 50 ms. $MS^2$ spectra for 2+ to 4+ charged species were acquired using: HCD fragmentation, top 10, resolution 17,500, AGC $5{\times}10^4$, maximum injection time 100 ms, isolation window 1.2 m/z, and normalized collision energy (NCE) of 27. The minimum AGC target was set at $2{\times}10^3$, which corresponds to a $2{\times}10^4$ intensity threshold.

TMT-labeled high-pH peptide fractions were analysed by Orbitrap Eclipse MS (Thermo Scientific) with on-line separation on a reversed-phase nanoLC column (450 mm × 0.075 mm ID) packed with ReprosilPur C18AQ (Dr Maisch, 3 µm particles) at 40 °C. A 60 min (TMT i) or 120 min (TMT ii–iv) gradient of 3–40% ACN, 0.1% FA at 300 nL/min was delivered via a Dionex UltiMate 3000 nanoHPLC system. Mass spectra were acquired in SPS $MS^3$ mode using a 3 s cycle time with the following settings: $MS^1$ — 120k resolution, max IT 50 ms, AGC target 400,000; $MS^2$ — IW 0.7 CID fragmentation, CE 35%, max IT 35 ms, turbo scan rate, AGC target 10,000; $MS^3$ — HCD fragmentation, CE 55%, 30k resolution, max IT 54 ms, AGC target 250,000.

**Data analysis.** LC-MS raw files were processed with Proteome Discoverer 2.5 using Sequest HT and Percolator, searching against UniProt Human Swissprot (UniProtKB 2021_01, canonical) and a PD

contaminant list (2015_5). Default settings were used, allowing 2 missed tryptic cleavages, with carbamidomethyl (C, fixed), oxidation (M, variable), acetyl/M-loss/M-loss+acetyl (protein N-term, variable), and, for the isobaric-labeled experiment, TMT 6-plex (K, peptide N-term, fixed). For 'complex' samples, the mouse (Swissprot, canonical) and *Drosophila melanogaster* (Swissprot and Trembl, canonical, 7227) proteomes were searched. For FFPE samples, methyl lysine was included as a variable modification. FTMS and ITMS spectra were searched with 0.02 and 0.5 Da fragment mass tolerances, respectively. Proteome Discoverer was used to determine protein and peptide identifications ($q < 0.01$), CV values, TMT quantitation and protein abundances. TMT ratios were determined without normalization (to assess technical effects), but corrected for batch-specific isotope impurities, with no imputation, minimum or missing values used. Minora feature detector was used for label-free quantitation. No normalization was applied to assess fully technical effects. Default setting were otherwise used. Proteome Discoverer was also used to assess differential protein recovery with *p*-values determined by multiple test-corrected *t*-test to determine the significance of observations of individual proteins across the replicates. One-way ANOVA and Tukey multiple comparisons test correction (GraphPad Prism 9.0) were used to determine significance between protein and peptide identification numbers (summarized in **Figure 1** and **Table S1**) with two-tailed Welch's *t*-test applied to paired analyses. $R^2$ values were determined as the squared Pearson product-moment correlation coefficient using Microsoft Excel. For the analysis of physicochemical property distributions, one-way ANOVA followed by Dunnett's multiple comparisons test, compared to the background TMT proteome, was performed to assess significant deviation from an expected distribution. Relative median protein recovery % was determined from the Proteome Discoverer and TMT-derived abundances calculated for each protein and isobaric label, with each percentage calculated relative to the highest reporter channel median protein abundance per 6-plex.

The MS proteomics data have been deposited to the ProteomeXchange Consortium (http://proteomecentral.proteomexchange.org) via the PRIDE partner repository (2) with the dataset identifier PXD032095 and, for validation work, PXD028736 and PXD028768. Proteomics data are summarised in **Table S1** and detailed in **Table S2-S20**.

Gene Ontology (GO) term enrichment analysis and functional annotation enrichment was performed with DAVID version 6.8. An addition analysis was performed with GO-SLIM. Terms were filtered to include those with Benjamini-adjusted significance ($p < 0.05$). Transmembrane proteins were defined by UniProt using the SUBCELLULAR LOCATION terms 'Single-pass type I membrane protein', 'Single-pass type II membrane protein', and 'Multi-pass membrane protein'. For protein solubility analysis, the UniProt Human Swissprot proteome was submitted to the CamSol Intrinsic tool for the calculation (at pH 7.0) of protein solubility and generic aggregation propensity, with a score generated for each protein sequence (3). Hydrophobicity (GRAVY score) was calculated by the PROMPT tool (4), and isoelectric points from ProteomePI (5).

## Supplementary Methods for SP4 validation work (Figure 3 A–D)

**Table S21**. Summary of the methodologies used by each validation lab

| | Lab 1 | | Lab 2 | Lab 3 |
|---|---|---|---|---|
| SP3 user | Yes | | No | Yes |
| Protein input ($\mu$g) | 1, 10, 250 | 250 | 25 | 50 |
| Sample type | Jurkat lysate | HEK293 | HEK293 | E14 murine ESC |
| Final protein conc. ($\mu$g/$\mu$L) | 0.1, 0.5, 1.25 | 1 | 2.5 | 0.5 |
| Replicates | $n$ = 2/3 | $n$ = 3 | $n$ = 3 | $n$ = 5 |
| Lysis buffer | RIPA | | 'SP3' | RIPA |
| Digestion method | Trypsin, O/N | Detailed above | Trypsin, O/N | Trypsin/Lys-C, 2 h, 70 °C |
| Other details | Acetone: overnight at −20 °C | | - | Rapid digestion buffer |
| Peptide injection (ng) | 100, 1000, 1000 | 1000 | 100 | 1000 |
| MS | Fusion Lumos | | QE+ | QE HF-X |
| Data processing | Pulsar (Biognosys) | | PD 2.1 | MaxQuant |

### LAB 1

### Lysate preparation (Experiment 1)

Jurkat immortalized human T cell lysate was prepared and diluted with RIPA lysis buffer (150 mM NaCl, 1.0% IGEPAL CA-630, 0.5% sodium deoxycholate, 0.1% SDS, 50 mM HEPES, pH 8.0, Protease inhibitor cocktail, (Calbiochem set III, 539134)) to 1.25 $\mu$g/$\mu$L. Three different masses of Jurkat protein lysate in RIPA lysis buffer were prepared: 250 $\mu$g (1.25 $\mu$g/$\mu$L) ,10 $\mu$g (0.5 $\mu$g/$\mu$L), and 1 $\mu$g (0.1 $\mu$g/$\mu$L). Each experiment was performed as discrete technical triplicates, e.g., with 3 separate aliquots of 250 $\mu$g of protein processed for each method.

† In the case of Fig 3A (1 $\mu$g, acetone overnight, and SP4-GB), only two experiments are present due to a technical failure during the injections of the entire 1 $\mu$g peptide sample, resulting in no material remaining for a repeat injection.

### Sample preparation (Experiment 1)

**Treatment.** Cell lysate was reduced with 5 mM DTT (30 min at 25 °C) and then treated with 5 mM iodoacetamide (30 min, 25 °C in the dark). Proteins were recovered by one of the four methods:

Acetone Precipitation. Proteins were precipitated by adding ice-cold acetone (4× sample volume, overnight, −20 °C). Protein pellets were obtained by centrifugation (18,000*g* for 10 min at 4 °C) and washed (2×) with the same volume of ice-cold 80% acetone/water (with sonication between washes). The final wash liquid was aspirated, and samples were air-dried for 20 min. Each sample was resuspended in 50 mM HEPES (250 $\mu$L for 250 $\mu$g and 20 $\mu$L for 10/1 $\mu$g). Samples were sonicated and vortexed to re-dissolve the pellet.

Seramag SP3 Beads. A stock of SP3 beads was prepared at 50 mg/mL by combining equivalent volumes of hydrophobic bead slurry and hydrophilic bead slurry. The resulting slurry was washed (3×) water, (3×) 50 mM HEPES. SP3 beads were added to cell lysate in bead:protein ratio of 10:1 (w/w) and distributed through gentle pipetting. The volume of the mixture was doubled with absolute ethanol and shaken (800 rpm for 10 min at RT). Tubes were placed on the magnetic separator and allowed to separate. The protein–bead aggregates were washed 3× while remaining on the magnetic rack with an equivalent total precipitating reaction volume of 70% ethanol/water (for 1 $\mu$g: 20 $\mu$L, 10 $\mu$g: 40 $\mu$L, and 250 $\mu$g: 400 $\mu$L) and then gently reconstituted by pipette with 50 mM HEPES to a final concentration of 250 $\mu$g (1.0 $\mu$g/$\mu$L), 10 $\mu$g (0.5 $\mu$g/$\mu$L), and 1 $\mu$g (0.1 $\mu$g/$\mu$L).

<u>ReSyn HILIC Beads.</u> A stock of MagReSyn HILIC beads (ReSyn Biosciences) was supplied at 50 mg/mL. The resulting slurry was washed with water (3×) and 50 mM HEPES (3×). ReSyn beads were added to cell lysate in bead:protein ratio of 10:1 (w/w) and distributed through gentle pipetting. The volume of the mixture was doubled with 200 mM ammonium formate pH 4.5, 30% ACN mixtures (binding buffer), and the tubes were shaken (800 rpm for 30 min). Tubes were placed on the magnetic separator and allowed to separate. The protein–bead aggregates were washed 3× while remaining on the magnetic rack with an equivalent total precipitating reaction volume of 95% ACN (for 1 $\mu$g: 20$\mu$L, 10 $\mu$g: 40 $\mu$L and 250 $\mu$g: 400 $\mu$L) and then gently reconstituted by pipette with 50 mM HEPES to a final concentration of 250 $\mu$g (1.0 $\mu$g/$\mu$L), 10 $\mu$g (0.5 $\mu$g/$\mu$L), and 1 $\mu$g (0.1 $\mu$g/$\mu$L).

<u>Glass Beads.</u> 100 mg of glass beads was distributed in 1 mL of Ultrapure water. This slurry was vortexed and centrifuged (16,000*g* for 2 min at 4 °C). The buoyant beads were gently aspirated to leave a glass bead pellet. This process was repeated with ACN (1×), 50 mM HEPES (1×) and Ultrapure water (2×). On the final wash, beads were resuspended in 1 mL of Ultrapure water, and a bead concentration of 50 mg/mL was assumed. Glass beads were added to cell lysate in bead/protein ratio of 10:1 (w/w) and distributed through gentle vortexing.  ACN was added to a final concentration of 80%. Upon addition of ACN, the mixture was again gently vortexed and then the tubes were centrifuged (16000*g* for 3 min at 4 °C (2×, with tubes spun in between)). The liquid was gently aspirated, and the beads were washed 3× with the equivalent volume of 80% ethanol/water (for 1 $\mu$g: 50 $\mu$L, 10 $\mu$g: 100 $\mu$L, and 250 $\mu$g: 1000 $\mu$L). Beads were reconstituted with additional sonication with 50 mM HEPES to a final concentration of 250 $\mu$g (1.0 $\mu$g/$\mu$L), 10 $\mu$g (0.5 $\mu$g/$\mu$L), and 1 $\mu$g (0.1 $\mu$g/$\mu$L).

**Digestion.** For the solely trypsin samples, digestion with trypsin (1:100 enzyme/protein; Promega) was carried out overnight at 37 °C.

**Recovery of Peptides from Beads**

<u>Seramag SP3 Beads / ReSyn Beads.</u> Tubes were placed on the magnetic separator and the peptide mixture was carefully pipetted off and dispensed into a fresh microcentrifuge tube.

<u>Glass Beads.</u> Tubes were centrifuged (16,000*g* for 3 min at 4 °C (2×, with tubes spun in between)) and the peptide mixture was carefully pipetted off and dispensed into a fresh microcentrifuge tube.

## <u>Lysate Preparation (Experiment 2)</u>

HEK293T lysate was prepared and diluted with RIPA lysis buffer to 1.25 $\mu$g/$\mu$L. 250 $\mu$g samples were prepared at 1 $\mu$g/$\mu$L and each experiment was performed at least in biological triplicate.

## <u>Sample Preparation (Experiment 2)</u>

**Treatment.** Cell lysate was reduced with 5 mM DTT (30 min at 25 °C) and then treated with 5 mM iodoacetamide (30 min at 25 °C in the dark). Proteins were then recovered by one of the two methods:

<u>Acetone Precipitation.</u> An analogous procedure to Experiment 1 was used up to point of re-dissolving the pellet. Each pellet was resuspended in the following volumes and buffers: 250 $\mu$L of 50 mM HEPES for trypsin only and 125 $\mu$L of 50 mM HEPES with 1 M guanidinium hydrochloride for Lys-C/trypsin. Samples were sonicated and vortexed periodically to re-dissolve the pellet.

<u>Glass Beads.</u> An analogous procedure to Experiment 1 was used up to point of reconstituting the beads. Beads were re-distributed *via* sonication with the following volumes and buffers: 250 $\mu$L of 50 mM HEPES for trypsin only and 125 $\mu$L of 50 mM HEPES with 1 M guanidinium hydrochloride for Lys-C/trypsin. Tubes were centrifuged (16,000*g* for 3 min at 4 °C (2×, with tubes spun in between)) and the peptide mixture was carefully pipetted off and dispensed into a fresh microcentrifuge tube.

**Digestion.** For the solely trypsin samples, digestion with trypsin (1:100 enzyme/protein; Promega) was carried out overnight at 37 °C.

For the Lys-C/trypsin samples, digestion with Lys-C (1:100 enzyme:protein; Wako) was carried out for 4 h at 37 °C, followed by 1:2 dilution with 50 mM HEPES and a secondary digestion with trypsin (1:100 enzyme:protein; Promega) performed overnight at 37 °C.

**Data Acquisition (Both Experiments)**

Assuming 100% recovery, 1 $\mu$g of each peptide mixture was added to 200 $\mu$L of 0.1% formic acid on a prepared Evotip (Evosep Biosystems) and run on an Evosep One LC connected to the Orbitrap Fusion MS instrument using 44 min LC-MS/MS gradient in DDA mode as described: the transfer capillary set to 300 °C and 2.2 kV applied to the nanospray needle (Evosep Biosystems). $MS^1$ data was acquired in the Orbitrap Fusion with a resolution of 60k, a max injection time of 20 ms, and an AGC target of $1\times10^6$, in positive ion mode, with profile spectra, over the mass range 375–1200 m/z. A charge state inclusion of precursors with 2–6+ charges was applied with the MIPS mode (Peptide) active, a dynamic exclusion of 15 s, intensity threshold of $5\times10^4$, and isolation carried out in the quadrupole with a width of 1.4 Da. For fragmentation, HCD energy of 32% was applied and $MS^2$ were acquired in the Orbitrap with 15k resolution, max injection time of 22 ms and an AGC target of $1\times10^6$ in centroid mode.

**Data Analysis (Both Experiments)**

For sample-specific spectral library generation, data was acquired from samples from each condition in data-dependent acquisition (DDA) mode. The data were searched against the human Uniprot database using the Pulsar search engine (Biognosys AG). The following modifications were included in the search: Carbamidomethyl (C) (Fixed) and Oxidation (M)/Acetyl (Protein N-term) (Variable). A maximum of 2 missed cleavages for trypsin were allowed. The identifications were filtered to satisfy FDR of 1% on peptide and protein level. Protein Group, Peptide and Precursor numbers were reported based on the library generated by the search.

**LAB 2**

Same as in main methods, with 3× 25 $\mu$g (10 $\mu$L of 2.5 $\mu$g/$\mu$L) preparations of HEK293 lysate for SP3, SP4-BF and SP4-GB.

**LAB 3**

**Comparison of SP3 and SP4 sample processing methods.** E14 murine embryonic stem cells were lysed in RIPA buffer (150 mM NaCl, 1.0% IGEPAL CA-630, 0.5% sodium deoxycholate, 0.1% SDS, 50 mM Tris, pH 8.0) by pipetting and sonication. The lysates were clarified by centrifugation (20,000*g* for 10 min at 4°C) and protein concentrations were determined by BCA assay. Aliquots ($n$ = 5 per experimental condition) corresponding to 50 $\mu$g of total protein were removed and diluted (1:1) with 20 mM HEPES, pH 8.5 buffer. Reduction with 5 mM TCEP final concentration was carried out at 37 °C for 45 min and alkylation with 20 mM 2-chloroacetamide (30 min at 25 °C). SP3 and SP4 protocols were carried out as described in the main methods. Following the respective processing methods, rapid digestion buffer (150 $\mu$L per sample, Promega VA 1061) was added followed by 5 $\mu$g Lys-C/trypsin mixture (Promega VA1061). Protein digestion was carried out at 70 °C with shaking (800 rpm) for 2 h. Samples were removed from the incubator and cooled on ice. Acidification was achieved by addition of

10% TFA (final concentration: 0.25%) and glass or magnetic beads were removed by centrifugation (20,000*g* for 5 min at 25 °C). Supernatants were transferred to sample vials and analysed by LC-MS/MS.

**Liquid chromatography-tandem mass spectrometry (LC-MS/MS) analysis.** Sample aliquots corresponding to 1 μg total digest were injected on a U3000 RSLC nano-liquid chromatography system onto a trapping column (Thermo Acclaim Pepmap 100, 0.1 mm × 20 mm, 164564) at a flow rate of 8 μL/min with loading buffer (2% ACN, 0.1% TFA). Following valve switch peptides were eluted onto an analytical column (Thermo EasySpray column, 0.075 mm × 500 mm, ES803A) by applying a linear multi-step gradient (buffer A: 5% DMSO, 0.1% formic acid; buffer B: 75% ACN, 5% DMSO, 0.1% formic acid) at a flow rate of 250 nL/min and a column temperature of 40 °C: 1% B [0–5 min], 22% B [75 min], 42% B [95 min], 87% B [95.1 min]. The elution gradient was followed by column wash and equilibration steps.

The Q-Exactive HF-X mass spectrometer was operated in positive ionisation mode at a spray voltage of 1.6 kV. DDA was carried out with a top 30 method, automatic gain control targets of $3\times10^6$ ($MS^1$) and $5\times10^4$ ($MS^2$) ions and maximum accumulation times of 25 ms ($MS^1$) and 50 ms ($MS^2$), respectively. Dynamic exclusion of fragmented precursors was enabled for 50 s.

**Data processing and analysis.** Raw data files were processed with MaxQuant version 1.6.10.43 and database searches carried out against a Swissprot *Mus musculus* database (version 2020.11.11, 17,056 entries). Settings included trypsin digestion with up to two missed cleavages, and a false discovery rate (FDR) of 1% for peptide spectrum matches and protein identifications. Protein N-terminal acetylation, methionine oxidation and peptide N-terminal glutamine to pyroglutamate conversion were enabled as variable modifications and cysteine carbamidomethylation as a fixed modification. The 'match between runs' option was enabled within experimental conditions (SP3 or SP4 digests) with match and alignment time windows of 0.7 and 20 min, respectively.

**Supplementary References**


1. Doellinger J, Schneider A, Hoeller M, Lasch P. Sample Preparation by Easy Extraction and Digestion (SPEED) - A Universal, Rapid, and Detergent-free Protocol for Proteomics Based on Acid Extraction. Mol Cell Proteomics. 2020;19(1):209-22.
2. Perez-Riverol Y, Csordas A, Bai J, Bernal-Llinares M, Hewapathirana S, Kundu DJ, et al. The PRIDE database and related tools and resources in 2019: improving support for quantification data. Nucleic Acids Res. 2019;47(D1):D442-D50.
3. Sormanni P, Aprile FA, Vendruscolo M. The CamSol method of rational design of protein mutants with enhanced solubility. J Mol Biol. 2015;427(2):478-90.
4. Schmidt T, Frishman D. PROMPT: a protein mapping and comparison tool. BMC Bioinformatics. 2006;7:331.
5. Kozlowski LP. Proteome-pI: proteome isoelectric point database. Nucleic Acids Res. 2017;45(D1):D1112-D6.

# SP4 (Solvent precipitation SP3) protocol

## Glass bead preparation (optional):

- 9–13 μm glass spheres/beads
  (e.g., https://www.sigmaaldrich.com/catalog/product/aldrich/440345)
  Glass beads broadly improved recovery, digestion efficiency and reproducibility, but are not required
- Suspend 100 mg in 1 mL of Ultrapure water, vortex until suspended fully, and pellet at > 500*g* for 1 min.
  Of note: approximately 50% of the beads are buoyant, and will not pellet, and should be removed over the course of these wash steps. Additionally, small amounts of metal in the beads can be removed by magnet or acid wash but had no effect on the performance of the beads. Larger scale preps are possible but may require additional washes due to buoyant beads.
- Resuspend, vortex and wash with ≥ 1 mL of: 100% acetonitrile (ACN) (1×), 100 mM ABC* (1×), and Ultrapure water (≥ 2×) ensuring no unpelleted beads remain. * or equivalent digestion buffer.

Then either:

- **A.** Resuspend beads in 0.9 mL of Ultrapure water to 50 mg/mL.
  given ~50% of beads are retained

***or***

- **B.** Resuspend beads in 0.9 mL acetonitrile to 50 mg/mL (recommended).
  - Avoids protein dilution from beads in water.
  - Ensures uniform bead dispersion
  - Dilute beads to at least 2.5× [protein] (so bead:protein is 10:1 from 4 volumes of bead–ACN suspension)

This will be sufficient to prepare 50 mg of protein—excess can be stored at 4 °C.
(with 0.2% sodium azide, if in water for an extended period)

## Lysate/protein solution prep recommendations

- SP4 is broadly compatible with the majority of lysis buffers as for SP3 or acetone precipitation
  Tested with:
  - 5% total detergent ‘SP3 lysis buffer’
    - (50 mM HEPES pH 8, 1% SDS, 1% Triton X-100, 1% IGEPAL CA-630, 1% Tween 20, 1% sodium deoxycholate, 50 mM NaCl, 5 mM EDTA, 1% (v/v) glycerol, and 1× protease inhibitors)
  - 8 M urea (diluted to 2 M prior to ACN addition)
  - TFA/Tris diluted 1:1 with water as described for the ‘SPEED’ method
- For best results with SP4, protein concentration should be as high as possible (0.25–5 μg/μL).
  - For lower concentrations or where highest possible recovery is required, longer precipitation reactions, carboxylate-modified beads, pre-chilled ACN, and centrifugation at 4 °C may help yields.
- DNA shearing (e.g., by sonication), protease inhibitors, & lysate clearance are recommended.

## SP4 protocol recommendations

- The use of the smallest possible tube will help create a denser pellet, e.g., 500 μL tube for samples of less than 50 μL.
- Liquids should be kept low in the tube, with losses/contamination possible from tube walls/lid.
- Set vortex to < 500 rpm for very gentle mixing.
- Pipette ACN directly into the sample to ensure rapid mixing, but do not touch the ACN–sample mix with the tip.
- Use the tube hinge to orientate the location of the pellet (fixed angle rotors).
  - Initially orientate the tube hinge inwards during the pellet precipitation and turn 180° after 2.5 min will give a denser pellet and less risk of loss from fragile wall adhesion.
- During wash removals, avoid touching the tube walls with the tip as precipitation may occur on them, pipette slowly and avoid agitating the pellet.
- If adding beads, ensure they maintain a uniform suspension in water/ACN by pipetting up and down at least once between additions.
- Organic solvent for aggregation/washes appears interchangeable between ethanol, ACN, IPA, & acetone (Ref. 2).

## SP4 Protocol

1. Aliquot reduced/alkylated protein mixture/lysate into a fresh LoBind-type microcentrifuge tube.
    - Volumes and conditions are given for the example of 10 $\mu$g protein in 10 $\mu$L of 1 $\mu$g/$\mu$L lysate.
2. Options (choose one):

**2a. Bead-free**

- Add 4 volumes of ACN.
    - E.g., 40 $\mu$L for 10 $\mu$L sample

**2b. Glass beads (in water)**

- Add 50 $\mu$g/$\mu$L beads (water-suspended) at 10:1 beads:protein and vortex.
    - E.g., 100 $\mu$g (2 $\mu$L) beads
- Add 4 volumes of ACN
    - E.g., 48 $\mu$L to 12 $\mu$L sample:bead mix

**2c. Glass beads (in ACN) (recommended)**

- Adjust beads to 2.5× protein concentration.
    - E.g., 2.5 $\mu$g/$\mu$L for 1 $\mu$g/$\mu$L sample
- Add 4 volumes of this ACN:bead suspension.
    - E.g., 40 $\mu$L for 10 $\mu$L sample

3. Ensure complete mixing (without pipette mixing, e.g., by consistent ACN addition, or < 500 rpm vortex for 5 s).
4. Centrifuge for 5 min at 500–16,000*g*.
5. Remove supernatant by pipetting slowly and remove a consistent volume of 90–95%. Avoid disturbing beads/pellet.

E.g., for a 50 $\mu$L total precipitation reaction remove 45 $\mu$L

6. Wash with 80% ethanol, volume ≥ 1.5× total precipitation volume (or at least 180 $\mu$L)
    - Pipette gently down the side opposite the hinge/pellet to avoid disturbance, do not vortex/resuspend.
7. Centrifuge for 2 min at 16,000*g*.
8. Remove 90–95% of wash.

E.g., leaving ~5–10 $\mu$L during washes

9. Repeat wash steps for a total of 3 washes.
10. Remove >= 95% of final wash.
    - For larger volumes a final 2 min spin will help with removal of excess wash.

E.g., leaving < 5 $\mu$L after final wash aspiration

11. Add preferred digestion buffer, e.g., 20–100 mM ABC or TEAB (pipette mixing will cause losses)
12. Add preferred digestion enzyme, e.g., trypsin/Lys-C at a 1:10 to 1:100 enzyme:protein ratio.
    - A digestion buffer/enzyme master mix will reduce variability and simplify pipetting—keep on ice.
    - Use a volume equivalent to ~0.5–2× the total precipitation volume.

      E.g., 25–100 $\mu$L for 50 $\mu$L precipitation reaction
    - In-bath sonication (5–10 min) can help to disrupt the pellet and increase surface area.
    - Larger bead-free pellets may require additional agitation to resuspend but keep sample low in tube.
    - 18 h digestion consistently worked without pellet resuspension for < 25 $\mu$g protein.
13. Incubate in a Thermomixer at 1000 rpm at desired conditions, e.g., for 18 h at 37 °C.
    - Beads were compatible with 2 h @ 47 °C using 1:10 trypsin or 2 h @ 70 °C (rapid digestion buffer), ensuring resuspension

## Peptide collection

- Centrifuge the peptide mixture at 500–16,000*g* for 2 min & collect peptide supernatant.
- For maximum recovery, rinse pellet/tube in an equal volume of digestion buffer added above.
    - A final centrifugation step may be required to ensure no beads are carried over.
- Peptides solution at this stage is clean enough to be:
    - Acidified (e.g., by 0.1–1% formic acid or trifluoroacetic acid) for direct LC-MS injection.
    - Dried by vacuum concentration to provide near-pure peptides.

**Protocol References**

1. Hughes CS, Moggridge S, Muller T, Sorensen PH, Morin GB, Krijgsveld J. Single-pot, solid-phase-enhanced sample preparation for proteomics experiments. Nat Protoc. 2019;14(1):68-85.
2. Moggridge S, Sorensen PH, Morin GB, Hughes CS. Extending the Compatibility of the SP3 Paramagnetic Bead Processing Approach for Proteomics. J Proteome Res. 2018;17(4):1730-40.
3. Dagley LF, Infusini G, Larsen RH, Sandow JJ, Webb AI. Universal Solid-Phase Protein Preparation (USP(3)) for Bottom-up and Top-down Proteomics. J Proteome Res. 2019;18(7):2915-24.
4. Batth TS, Tollenaere MAX, Ruther P, Gonzalez-Franquesa A, Prabhakar BS, Bekker-Jensen S, et al. Protein Aggregation Capture on Microparticles Enables Multipurpose Proteomics Sample Preparation. Mol Cell Proteomics. 2019;18(5):1027-35.
5. Sielaff M, Kuharev J, Bohn T, Hahlbrock J, Bopp T, Tenzer S, et al. Evaluation of FASP, SP3, and iST Protocols for Proteomic Sample Preparation in the Low Microgram Range. J Proteome Res. 2017;16(11):4060-72.
6. Holger lab (MRC unit) SOP.
